# Supplementary material for: Protocol for a randomised controlled trial investigating an intervention to boost decentering in response to distressing mental experiences during adolescence: the decentering in adolescence study (DECADES)
Source: BMJ Open. 2022 Mar 30;12(3):e056864. doi: 10.1136/bmjopen-2021-056864 (PMC8968529; doi:10.1136/bmjopen-2021-056864)
Supplement: Supplementary data [file bmjopen-2021-056864supp001.pdf]

Daily_Diaries_Intervention

Start of Block: Intro

Q69 Please Enter Your ID Number

Remember, this is the last three letters of your phone number, then the last three characters of your postcode, then the last letter of your surname.

____

Q68 Thanks for your participation in our study so far. We have some questions about your day today that we'd like you to answer.

End of Block: Intro

Start of Block: Block 1

Q50 Thinking back over the day, please rate to what extent you have felt this mood (**happy**) today.

	1 - not at all (1)	2 (2)	3 (3)	4 (4)	5 (5)	6 (6)	7 - very much (7)
1 (1)	○	○	○	○	○	○	○

Q51 How much time in the day do you feel you have spent in this mood (**happy**).

	1 - very little (1)	2 (2)	3 (3)	4 (4)	5 (5)	6 (6)	7 - most of the day (7)
1 (1)	○	○	○	○	○	○	○

Bennett MP, *et al. BMJ Open* 2022; 12:e056864. doi: 10.1136/bmjopen-2021-056864

Page Break

Q52 Thinking back over the day, please rate to what extent you have felt this mood (**lively**) today.

	1 - not at all (1)	2 (2)	3 (3)	4 (4)	5 (5)	6 (6)	7 - very much (7)
1 (1)	○	○	○	○	○	○	○

Q53 How much time in the day do you feel you have spent in this mood (**lively**).

	1 - very little (1)	2 (2)	3 (3)	4 (4)	5 (5)	6 (6)	7 - most of the day (7)
1 (1)	○	○	○	○	○	○	○

Page Break

Bennett MP, *et al. BMJ Open* 2022; 12:e056864. doi: 10.1136/bmjopen-2021-056864

Q54 Thinking back over the day, please rate to what extent you have felt this mood (**content**) today.

	1 - not at all (1)	2 (2)	3 (3)	4 (4)	5 (5)	6 (6)	7 - very much (7)
1 (1)	○	○	○	○	○	○	○

Q55 How much time in the day do you feel you have spent in this mood (**content**).

	1 - very little (1)	2 (2)	3 (3)	4 (4)	5 (5)	6 (6)	7 - most of the day (7)
1 (1)	○	○	○	○	○	○	○

Page Break

Bennett MP, *et al. BMJ Open* 2022; 12:e056864. doi: 10.1136/bmjopen-2021-056864

Q56 Thinking back over the day, please rate to what extent you have felt this mood (**satisfied**) today.

	1 - not at all (1)	2 (2)	3 (3)	4 (4)	5 (5)	6 (6)	7 - very much (7)
1 (1)	○	○	○	○	○	○	○

Q57 How much time in the day do you feel you have spent in this mood (**satisfied**).

	1 - very little (1)	2 (2)	3 (3)	4 (4)	5 (5)	6 (6)	7 - most of the day (7)
1 (1)	○	○	○	○	○	○	○

Page Break

Bennett MP, *et al. BMJ Open* 2022; 12:e056864. doi: 10.1136/bmjopen-2021-056864

Q58 Thinking back over the day, please rate to what extent you have felt this mood (**depressed**) today.

	1 - not at all (1)	2 (2)	3 (3)	4 (4)	5 (5)	6 (6)	7 - very much (7)
1 (1)	○	○	○	○	○	○	○

Q59 How much time in the day do you feel you have spent in this mood (**depressed**).

	1 - very little (1)	2 (2)	3 (3)	4 (4)	5 (5)	6 (6)	7 - most of the day (7)
1 (1)	○	○	○	○	○	○	○

Page Break

Bennett MP, *et al. BMJ Open* 2022; 12:e056864. doi: 10.1136/bmjopen-2021-056864

Q60 Thinking back over the day, please rate to what extent you have felt this mood (**bored**) today.

	1 - not at all (1)	2 (2)	3 (3)	4 (4)	5 (5)	6 (6)	7 - very much (7)
1 (1)	○	○	○	○	○	○	○

Q61 How much time in the day do you feel you have spent in this mood (**bored**).

	1 - very little (1)	2 (2)	3 (3)	4 (4)	5 (5)	6 (6)	7 - most of the day (7)
1 (1)	○	○	○	○	○	○	○

Page Break

Bennett MP, *et al. BMJ Open* 2022; 12:e056864. doi: 10.1136/bmjopen-2021-056864

Q62 Thinking back over the day, please rate to what extent you have felt this mood (**anxious**) today.

	1 - not at all (1)	2 (2)	3 (3)	4 (4)	5 (5)	6 (6)	7 - very much (7)
1 (1)	o	o	o	o	o	o	o

Q63 How much time in the day do you feel you have spent in this mood (**anxious**).

	1 - very little (1)	2 (2)	3 (3)	4 (4)	5 (5)	6 (6)	7 - most of the day (7)
1 (1)	o	o	o	o	o	o	o

Page Break

Bennett MP, *et al. BMJ Open* 2022; 12:e056864. doi: 10.1136/bmjopen-2021-056864

Q64 Thinking back over the day, please rate to what extent you have felt this mood (**irritable**) today.

	1 - not at all (1)	2 (2)	3 (3)	4 (4)	5 (5)	6 (6)	7 - very much (7)
1 (1)	○	○	○	○	○	○	○

Q65 How much time in the day do you feel you have spent in this mood (**irritable**).

	1 - very little (1)	2 (2)	3 (3)	4 (4)	5 (5)	6 (6)	7 - most of the day (7)
1 (1)	○	○	○	○	○	○	○

Page Break

Bennett MP, *et al. BMJ Open* 2022; 12:e056864. doi: 10.1136/bmjopen-2021-056864

Q66 Thinking back over the day, please rate to what extent you have felt this mood (**tense**) today.

	1 - not at all (1)	2 (2)	3 (3)	4 (4)	5 (5)	6 (6)	7 - very much (7)
1 (1)	○	○	○	○	○	○	○

Q67 How much time in the day do you feel you have spent in this mood (**tense**).

	1 - very little (1)	2 (2)	3 (3)	4 (4)	5 (5)	6 (6)	7 - most of the day (7)
1 (1)	○	○	○	○	○	○	○

End of Block: Block 1

Start of Block: Block 2

Q41 Now, we just have some final questions...

Q42 Did you complete any of the exercises from this programme today?

○ Yes (1)

○ No (2)

Display This Question:

If Did you complete any of the exercises from this programme today? = Yes

Q43 How difficult did you find these exercises?

- ◯ 1 - Not at all difficult (2)
- ◯ 2 (3)
- ◯ 3 (4)
- ◯ 4 - Somewhat difficult (5)
- ◯ 5 (6)
- ◯ 6 (7)
- ◯ 7 - Extremely Difficult (8)

Display This Question:

If Did you complete any of the exercises from this programme today? = Yes

Q44
Did you find the skills you've been learning to be relevant at any stage during the day?

- ◯ 1 - Not At All Relevant (1)
- ◯ 2 (2)
- ◯ 3 (3)
- ◯ 4 - Somewhat (4)
- ◯ 5 (5)
- ◯ 6 (6)
- ◯ 7 - Extremely (7)

Bennett MP, *et al. BMJ Open* 2022; 12:e056864. doi: 10.1136/bmjopen-2021-056864

Display This Question:

If Did you complete any of the exercises from this programme today? = Yes

Q45 During the day, were you able to apply the skills you've learned during the exercises?

- 1 - Not At All (1)
- 2 (2)
- 3 (3)
- 4 - Somewhat (4)
- 5 (5)
- 6 (6)
- 7 - Absolutely (7)

Display This Question:

If Did you complete any of the exercises from this programme today? = Yes

Q49
During the day, how effortful was it to apply the skills you have learned ?

- 1- Not at all (1)
- 2 (2)
- 3 (3)
- 4 - Somewhat (4)
- 5 (5)
- 6 (6)
- 7 - Extremely effortful (7)

End of Block: Block 2

Bennett MP, *et al. BMJ Open* 2022; 12:e056864. doi: 10.1136/bmjopen-2021-056864
