# Supplementary material for: Protocol for a randomised controlled trial investigating an intervention to boost decentering in response to distressing mental experiences during adolescence: the decentering in adolescence study (DECADES)
Source: BMJ Open. 2022 Mar 30;12(3):e056864. doi: 10.1136/bmjopen-2021-056864 (PMC8968529; doi:10.1136/bmjopen-2021-056864)
Supplement: Supplementary data [file bmjopen-2021-056864supp002.pdf]

Figure S1 – Affective Cognitive Control Task

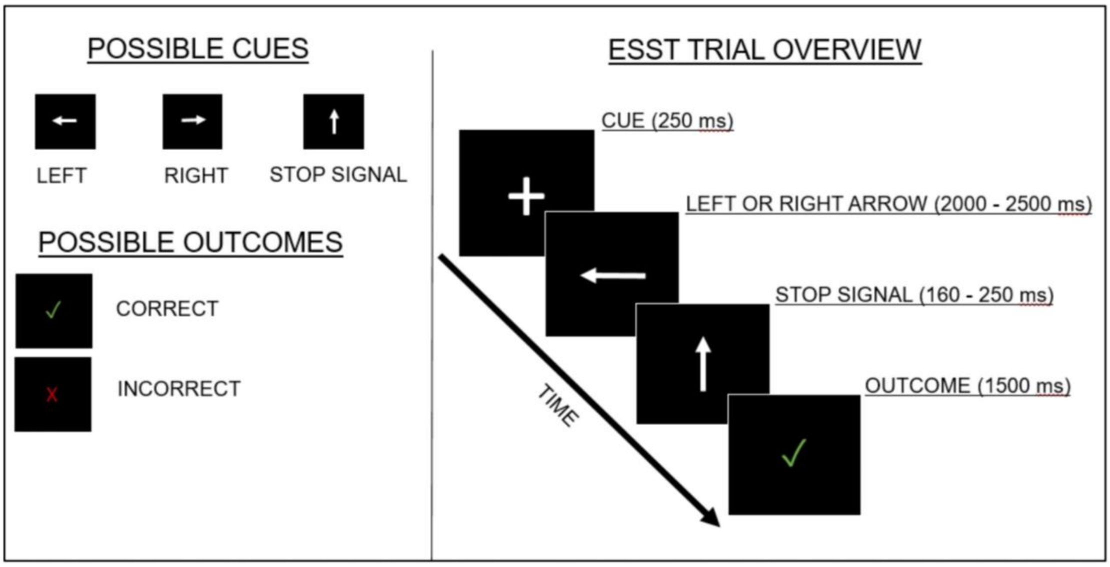

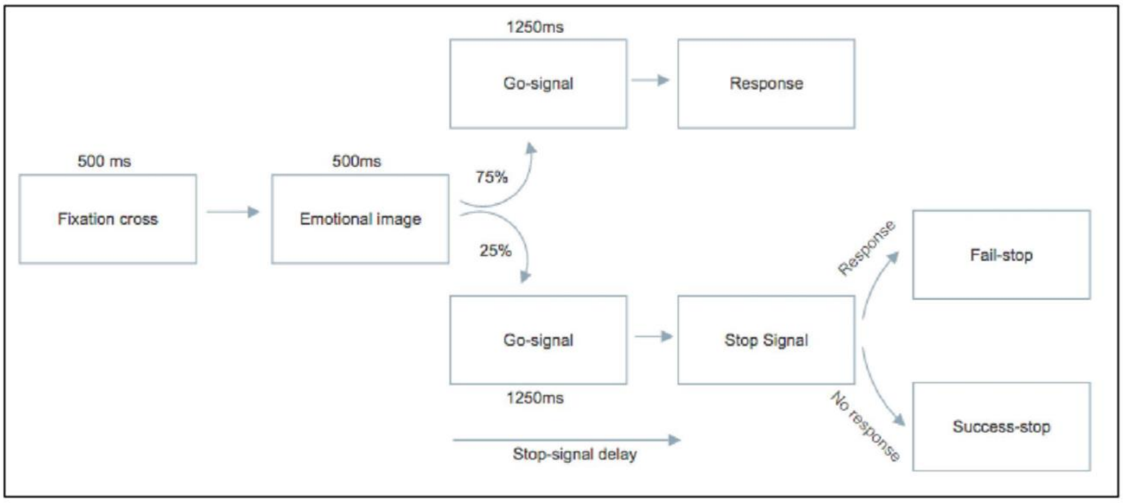

Across multiple trials, participants respond to a go-signal (e.g. press-left to a left arrow) unless it is followed by a stop-signal (an upwards arrow); these stop-signals follow 20% of go-signals. In the emotional stop-signal task (eSST) go-signals are preceded by a neutral or negative image from the IAPS (IAPS; Lang et al., 1992). We will measure accuracy and neurophysiological activities during response inhibition.
