# Supplementary material for: Protocol for a randomised controlled trial investigating an intervention to boost decentering in response to distressing mental experiences during adolescence: the decentering in adolescence study (DECADES)
Source: BMJ Open. 2022 Mar 30;12(3):e056864. doi: 10.1136/bmjopen-2021-056864 (PMC8968529; doi:10.1136/bmjopen-2021-056864)
Supplement: Supplementary data [file bmjopen-2021-056864supp003.pdf]

Figure S2 – Self Referential Processing Task

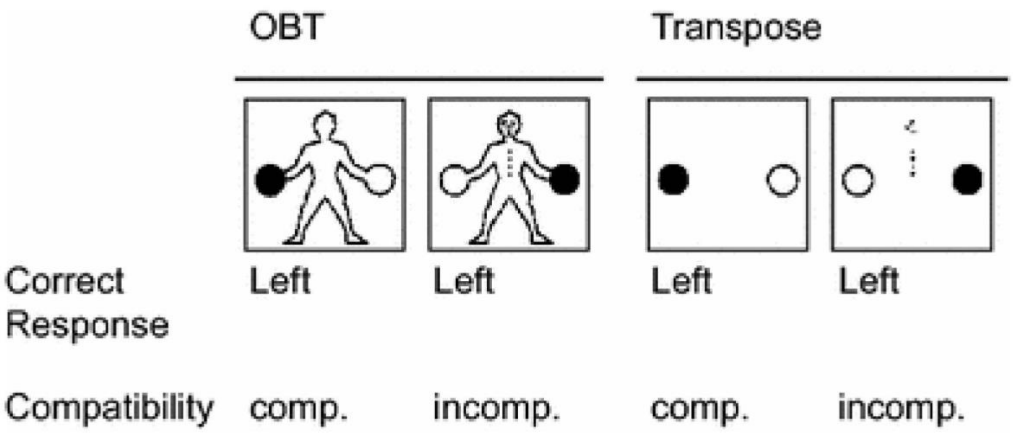


For example, the Own Body Task requires speeded spatial judgments made from the perspective of a schematic figure. The stimulus is a figure holding a black ball in one hand and a white ball in the other. The black ball was either in the figure's left or right hand and the figure could be facing either toward or away from the participant, but always in an upright orientation. Participants were instructed to imagine themselves in the body position of the figure, in order to judge whether the black ball was being held by the figure's left or right hand by pressing the corresponding response keys. We will measure how quickly participants are able to judge which hand the black ball is in, and whether they can correctly judge this.
