# Supplementary material for: Protocol for a randomised controlled trial investigating an intervention to boost decentering in response to distressing mental experiences during adolescence: the decentering in adolescence study (DECADES)
Source: BMJ Open. 2022 Mar 30;12(3):e056864. doi: 10.1136/bmjopen-2021-056864 (PMC8968529; doi:10.1136/bmjopen-2021-056864)
Supplement: Supplementary data [file bmjopen-2021-056864supp004.pdf]

Appendix A

Tim Dalgleish
Principal Investigator
MRC Cognition and Brain Sciences Unit
University of Cambridge
15 Chaucer Road,
Cambridge, CB2 7EF
Phone: 01223 766166
myriad@mrc-cbu.cam.ac.uk

**Participant Consent Form**

**Study title: Investigating the neuro-cognitive dynamics of decentring training and its impact on emotional reactivity: A Longitudinal and multimodal assessment.**

We are interested in how new therapeutic techniques like mindfulness and decentring affect your thoughts and feelings.

This study has been reviewed by the Cambridge Psychology Research Ethics Committee [PRE.2019.036]

**Please initial at the end of each line and then sign below to indicate that you agree to take part in the study:**

• Read and understood the information sheet for this study

...............

• Had the opportunity to consider the information and ask questions via the email address or phone number given at the top of this sheet

...............

• Had your questions answered satisfactorily

...............

• Understood that this project will require you to have an app installed on your smartphone for the duration of the project

...............

• Understood that your participation is voluntary and that he or she can withdraw at any time without giving a reason

...............

• Understood that your data and personal details will be stored on secure servers. At the end of the study, the data will be transferred and held at The MRC Cognition and Brain Sciences Unit in a secure database and will be accessed by the academic community

………………

• The MRC Cognition and Brain Sciences Unit will take all reasonable steps to protect your privacy. Your data will be coded and will not be associated with your name or information that could reveal your identity. This anonymised data will be made freely available to the wider academic community ……………

• Understood that all information will be treated as strictly confidential and handled in accordance with the provisions of the Data Protection Act 2018

……………

• Understood that in all instances where the results are disseminated they will remain anonymous

……………

## PLEASE USE BLOCK CAPITAL LETTERS WHEN FILLING IN THIS FORM

Your name……………………………………………………………………………………………………………………………………………………………

Your school/college…..………………………………………………………………………………………………………………………………………………..................

Gender ………………………. Year group……………………… Date of birth……………………………………..

Other information. Does you have any condition that may affect your performance on the tasks? If so please state: …………………………………………………………….

Would you like to be contacted in the future about participating in other studies (please circle)?
YES NO

Your email………………………………………………………………………………………………………………………………………………………

**Please sign below to indicate that you agree to take part in the study:**

Your signature: ……...................................................... Date: ................................
