# Supplementary material for: Protocol for a randomised controlled trial investigating an intervention to boost decentering in response to distressing mental experiences during adolescence: the decentering in adolescence study (DECADES)
Source: BMJ Open. 2022 Mar 30;12(3):e056864. doi: 10.1136/bmjopen-2021-056864 (PMC8968529; doi:10.1136/bmjopen-2021-056864)
Supplement: Author's manuscript [file bmjopen-2021-056864.draft_revisions.pdf]

BMJ Open

BMJ Open is committed to open peer review. As part of this commitment we make the peer review history of every article we publish publicly available.

When an article is published we post the peer reviewers' comments and the authors' responses online. We also post the versions of the paper that were used during peer review. These are the versions that the peer review comments apply to.

The versions of the paper that follow are the versions that were submitted during the peer review process. They are not the versions of record or the final published versions. They should not be cited or distributed as the published version of this manuscript.

BMJ Open is an open access journal and the full, final, typeset and author-corrected version of record of the manuscript is available on our site with no access controls, subscription charges or pay-per-view fees (http://bmjopen.bmj.com).

If you have any questions on BMJ Open's open peer review process please email
info.bmjopen@bmj.com

BMJ Open

Protocol for a randomized controlled trial investigating an intervention to boost decentering in response to distressing mental experiences during adolescence: The Decentering in Adolescence Study (DECADES)

Journal:	*BMJ Open*
Manuscript ID	bmjopen-2021-056864
Article Type:	Protocol
Date Submitted by the Author:	27-Aug-2021
Complete List of Authors:	Knight, Rachel; MRC Cognition and Brain Sciences Unit,<br>Bennett, Marc; MRC Cognition and Brain Sciences Unit<br>Dunning, Darren; University of Cambridge,<br>Archer-Boyd, Alan; MRC Cognition and Brain Sciences Unit<br>Clegg, Hannah; MRC Cognition and Brain Sciences Unit<br>Blakemore, Sarah-Jayne; University of Cambridge,<br>Lenaert, Bert; Maastricht University, Faculty of Healh, Medicine and Life Sciences<br>Dalmaijer, Edwin; MRC Cognition and Brain Sciences Unit<br>Ford, Tamsin; University of Cambridge, Psychiatry<br>Kuyken, Willem; University of Oxford, Psychiatry<br>Williams, J Mark; University of Oxford, Psychiatry<br>Watson, Peter; University of Cambridge, MRC Cognition and Brain Sciences Unit<br>So, Tierney; University of Cambridge<br>Wright, Gem; MRC Cognition and Brain Sciences Unit<br>Vainre, Maris; Cambridge University, MRC Cognition and Brain Sciences Unit<br>Dalgleish, Tim; Medical Research Council Cognition and Brain Sciences Unit,
Keywords:	Child & adolescent psychiatry < PSYCHIATRY, Anxiety disorders < PSYCHIATRY, Depression & mood disorders < PSYCHIATRY, Clinical trials < THERAPEUTICS, PREVENTIVE MEDICINE

SCHOLARONE™
Manuscripts

Protocol for a randomized controlled trial investigating an intervention to boost decentering in response to distressing mental experiences during adolescence: The Decentering in Adolescence Study (DECADES)

Bennett, M.P.[1*], Knight, R. [1*], Dunning, D.[1], Archer-Boyd., A.[1], Blakemore, S-J., Dalmaijer, E.[1], Ford. T.[2], Williams, J. M. G.[3], Clegg, H., [1] Kuyken, W.[3], So., T.[1], Wright, G[1], Lenaert, B.[4,5], Vainre, M. [1], Watson, P.,[1] MYRIAD team, & Dalgleish, T. [1,6]

1- Medical Research Council- Cognition and Brain Sciences Unit, University of Cambridge, UK.

2- Department of Psychiatry, University of Cambridge, UK.

3- Department of Psychiatry, University of Oxford, UK.

4- Faculty of Health, Medicine and Life Sciences, Maastricht University, Maastricht, NL.

5- Limburg Brain Injury Centre, Limburg, NL.

6- Cambridgeshire and Peterborough NHS Foundation Trust

*co-first authors

Trial number: ISRCTN14329613

Version: 4.3

Correspondence for this article should be addressed to Miss Rachel Knight or Dr. Marc Bennett, Medical Research Council- Cognition and Brain Sciences Unit, 15 Chaucer

For peer review only

Road, Cambridge, CB2 7EF. E-mail: Rachel.knight@mrc-cbu.cam.ac.uk, marc.bennett@mrc-cbu.cam.ac.uk

Data Statement

The datasets generated during and/or analysed during the current study will be stored in a publically available repository. Following the publication of the first manuscript, a fully anonymized dataset will be made available online on open-access databases whose servers are located within the UK or Europe. Data processing scripts and programming codes will also be made available online.

Conflict of Interest Statement

The authors declare no competing interests.

Funding

This project is funded by a Wellcome Strategic Award (Wellcome Trust, ref 104908/Z/14/z; awarded to TD, SJB, FT, WK, MW). The contribution of MB was partially supported by a Wellcome Trust Active Ingredients in Mental Heatlh Commission. RK is funded by an Economic and Social Research Council Doctoral Fellowship.

Keywords

Decentering, adolescence, psychological distress, intervention, ecological momentary assessment

For peer review only

The **MYRIAD** Team comprises Saz Ahmed, PhD, of University College London, Susan Ball, MSc, of University of Exeter, Nicola Dalrymple, MSc, of University of Oxford, Katie Fletcher, HSD, of University of Oxford, Lucy Foulkes, PhD, of University College London, Poushali Ganguli, MSc, of Kings College London, Cait Griffin, MSc, Kirsty Griffiths, MSc, of University of Cambridge, Konstantina Komninidou, BEd, of University of Oxford, Suzannah Laws, BSc, of University of Oxford, Jovita Leung, MSc, of University College London, Jenna Parker, MSc, of University of East Anglia, Blanca Piera Pi-Sunyer, MSc, of University College London, J. Ashok Sakhardande, BSc Hons, Jem Shackleford, MA, MSc, Kate Tudor, PhD, of University of Oxford, and Brian Wainman, BEng, of Plymouth University. These individuals have worked across the MYRIAD strategic award “Promoting Mental Health and Building Resilience in Adolescence: Investigating Mindfulness and Attentional Control”; they are acknowledged as group authors in this article for their substantial contributions to the project development, in accordance with the MYRIAD Dissemination Protocol

For peer review only

Abstract

Introduction. Decentering describes the ability to voluntarily adopt an objective self-perspective from which to notice internal, typically distressing, stressors (e.g. difficult thoughts, memories, and feelings). The reinforcement of this skill may be an active ingredient through which different psychological interventions accrue reductions in anxiety and/or depression. However, it is unclear if decentering can be selectively trained at a young age and if this might reduce psychological distress. The aim of the current trial is to address this research gap. **Methods and analysis.** Adolescents, recruited from schools in the UK and the EU (n = 57 per group, age range = 16-19 years), will be randomised to complete five-weeks of decentering training, or an active control group that will take part in a combination of light physical exercise and cognitive training. The co-primary training outcomes include a self-reported decentering inventory (i.e. the Experiences Questionnaire) and the momentary use of decentering in response to psychological stressors, using experience sampling. The secondary mental health outcomes will include self-reported inventories of depression and anxiety symptoms, as well as psychological wellbeing. Initial statistical analysis will use between-group analysis of covariance to estimate the effect of training condition on self-rated inventories, adjusted for baseline scores. Additionally, experience sampling data will be examined using hierarchical linear models. **Ethics and dissemination.** This study was approved by the Cambridge Psychology Research Ethics Committee, University of Cambridge (PRE.2019.109). Findings will be disseminated through typical academic routes including poster/paper presentations at (intern)-national conferences, academic institutes and through publication in peer-reviewed journals.

Trial registration number: ISRCTN14329613

For peer review only

Article Summary

Strengths and limitations of this study

Strengths.

- We aim to strengthen an active and pan-therapeutic skill that features across different psychological interventions, and may improve well-being across the spread of adolescent mental health, from suffering through to flourishing.

- The intervention will be highly accessible by capitalising on extant social media and streaming services.

- Ecological momentary assessment will facilitate a temporally sensitive investigation into the effects of decentering training in everyday life.

Limitations.

- A lack of face-to-face interaction between experimenters and participants may undermine adherence.

- The study may be demanding as it requires daily, albeit brief, engagement over a several weeks.

For peer review only

Introduction

Background

Anxiety and depression are a considerable public health challenge. Since these difficulties often begin before the age of 24, responding to this challenge requires a special focus on adolescence[1]. It has been suggested that an effective way to promote early mental health is through universal approaches[2-4]; these are interventions that can be offered across a broad community of young people, irrespective of symptom severity. The goal of such universal approaches is to not only reduce distress in young people experiencing symptoms, but also to prevent future anxiety and depression onset in those who may be asymptomatic. However, their effectiveness depends on how well an intervention focuses on psychological experiences and mechanisms that are relevant across the entire spectrum of mental health, from risk to resilience, and through to flourishing. One strategy is therefore to target widely relevant therapeutic skills that can help manage distressing inner experiences (e.g. unpleasant feelings thoughts memories), which can otherwise trigger short-term distress and long-term risk of anxiety and depression onset. Psychological decentering, or decentering for short, may be one such skill.

Psychological decentering is a key concept within psychological therapy and science[5]. It is characterised as an adaptive self-observation style wherein one can attend to, not just the mental content of distressing inner experiences, but also its underlying cognitive nature[6,7]. Thus, decentering relates to how a person interacts with the difficult inner events prompted by experiences in daily life – it involves the ability to generate an objective self-perspective wherein one can notice mental experiences as imperfect models of the real-world rather than precise reflections. For example, a person might notice “I am thinking I am depressed right now” instead of only noticing

For peer review only

and then believing the thought "I am depressed"[8]. Decentering resultantly restricts the disproportionate influence psychological stressors may have on affect, behaviour and sense-of-self. Indeed, brief therapeutic exercises that stimulate a decentred self-perspective have been shown to reduce negative emotional reactivity towards stressors like unpleasant memories and negative self-relevant statements[9-13]. Evidence also indicates self-reported decentering traits in adolescence are associated with fewer overall symptoms of anxiety and depression[14-17].

Decentering is a malleable skill that can be refined through psychological intervention[7]. Medium to large changes in self-reported decentering are reported in adults experiencing anxiety and depression following a range of psychological interventions. This includes cognitive behavioural therapy (CBT)[18,19], acceptance based approaches[20-22] and mindfulness training[23-25]. In fact, emerging evidence suggests that intervention-related increases in decentering are a common pathway through which different psychological interventions deliver reductions in anxiety and depression[21,26,27]. This has encouraged some investigations into whether or not decentering can be trained selectively so to improve mental health. For example, Travers-Hill et al. trained adults with a diagnosis of recurrent depression currently in remission but experiencing residual depression symptoms to practice self-distancing techniques in response to difficult autobiographical memories[28]. This resulted in a significant increase in self-reported measures of decentering relative to an active comparison group who learned strategies to manage maladaptive avoidance tendencies. Additionally, those who learned to apply this decentering-related technique reported less distress towards negative memories at post-intervention even in the absence of instructions to self-distance.

For peer review only

Therapeutic exercises that target decentering skills are already nested within different psychological interventions[7]. Emerging evidence also indicates that these components can be delivered in isolation so to selectively boost decentering and reduce risk of relapse in formerly depressed adults[28]. However, a remaining question is ‘can decentering skills be selectively reinforced during adolescents, and if so, through what therapeutic techniques?’ This is the primary research question guiding the current study. It is also unclear if boosting adolescent decentering skills might impact on emotion and mental health. The current study will therefore evaluate, as a secondary question, how decentering training impacts anxiety, depression and emotional unfolding in adolescents. This information will inform future research trials and ultimately help in the investigation of effective universal approaches in youth mental health. A straightforward way to address these questions might be to examine the impact of decentering training on trait-level measure of decentering and symptom severity at critical time points, e.g. pre and post-intervention design. However, this approach lacks the sensitivity required to detect subtle changes in decentering in response to momentary psychological stressors and their impact on emotion. A design that also includes ecological momentary assessment or experience sampling methods (ESM) could therefore facilitate a more ecological investigation of the use of decentering in everyday life as well as its impact on transient psychological stressors and emotional states.

Current study objectives

The primary aim of the current study is to investigate if decentering can be improved during adolescence via an intensive decentering training programme. This will be investigated (1a) at a trait level by examining changes in self-reported experience of decentering (via a standard decentering inventory) and (1b) at a

For peer review only

situational level by examining self-reported use of decentering skills in response to momentary psychological stressors (via ESM assessments). Here, repeated measures of participants' negative emotional reactivity towards momentary inner experiences (e.g. feelings, thoughts and memories) will be recorded over a five day baseline period and across the duration of the intervention. A secondary aim is to explore potential mental health outcomes associated with reinforced decentering skills. This will be investigated (2a) at the symptom level by examining relative change in self-rated anxiety, depression and psychological well-being and (2b) at the situational level by recording emotional reactivity towards momentary psychological stressors using ESM. A final exploratory aim is to investigate some of the cognitive correlates of decentering and training. Thus, this study will answer the following research questions: (1a) Is decentering training during adolescence associated with increased decentering reports relative to an active control condition? (1b) Is decentering training during adolescence associated with increased decentering in response to negative mental events relative to an active control condition? (2a) What is the impact of decentering training on youth mental health relative to an active control condition? (2b) What is the impact of decentering training on emotional reactivity towards momentary negative mental events relative to an active control? (3) What are the cognitive correlates of decentering training during adolescence?

This study will address these research questions in a cohort of adolescents who are at increased risk of depression. This is a first step towards our long-term goal to develop a universal intervention that targets psychological experiences and mechanisms that are broadly relevant in young people. Adolescents experiencing elevated symptoms of depression will be recruited from both the UK (e.g. sixth form colleges) and EU (e.g. secondary level education high schools) and will be randomly

For peer review only

assigned to a five week decentering training programme or an active control. Both programmes will be delivered remotely via extant social media platforms; this means they can be delivered at low-cost, are easily accessible and are flexible regarding other commitments of our participants. The decentering intervention comprises 10-15 minute of audio exercises to be made available each weekday through music/podcast streaming services, e.g. *Spotify*, *iTunes*, *Deezer*, *Podcast Addict.* Participants will practice a specific decentering technique each week, which has been adapted from either the literature or an extant psychological therapy. Participants will also practice brief mindfulness-based grounding exercises each week to encourage the monitoring of inner experiences like emotional thoughts, memories and feelings. The overall goal of this programme is therefore to teach adolescents different ways to generate an objective self-perspective from which they can interact with negative psychological events encountered in daily life. Rather than rehearsing decentering techniques, the active control condition comprises 10-15 minutes of gamified cognitive tasks for completion on a personal smartphone. In addition, participants in this condition will be given short physical movement routines to complete in lieu of mindfulness-based grounding.

Methods

Study design

The design is a randomised controlled feasibility trial (comparing a psychological decentering training to an active control programme) with school aged adolescents (aged 16-19 years). Self-rated inventories of training and mental health outcomes will be assessed at three time points: within one week prior to the start of programme (baseline); within the third week of training (mid-intervention); and one week after the programme has finished (post-intervention).

For peer review only

Participants and inclusion/exclusion criteria

Two groups of older adolescents (n = 48 per group; age range = 16–19 years) will be recruited from the UK and Ireland. Eligible participants must consent to completing (1) a five day ESM baseline assessment, (2) five weeks of the assigned training programme and (3) assessment measures at baseline, mid-intervention and post-intervention. In exchange for their time, participants will be compensated with shopping vouchers worth (1) £50 (for completing ESM measures), (2) £100 pounds (for completing five weeks of training) and (3) £50 (as a bonus for completing all assessments). Eligible participants must also report a score of 27 or above on the Centre for Epidemiological Studies-Depression Scale (CES-D). Our recent research in a similar sample indicates that this score equates to 1 SD above the mean (M = 19.73, SD = 7.62; age range = 11.20–18.20 years; *N* = 468). Participants must also have access to a laptop/desktop computer and a personal smartphone device.

Participants will be excluded if they: (1) currently take part in a regular yoga and/or mindfulness class/workshop, (2) have participated in prior formal meditation training or a mindfulness-based stress reduction course (3)are currently experiencing chronic illness (e.g. epilepsy, chronic pain, cancer), (4) lack fluency in English (5) have a recent diagnosis of, and are currently receiving medical/psychological treatment for, a mental health condition including (but not limited to) anxiety disorder, major depressive disorder or a traumatic stress disorder and (6) have a diagnosis of a neurodevelopmental condition such as Autism Spectrum Disorder or Attention Deficit/Hyperactivity Disorder. Both the decentering programme and active control programme require adolescents to be without severe hearing difficulties since both conditions require listening to audiotapes. As the control condition involves physical movement routines it may not be appropriate for specific individuals, such as

For peer review only

individuals with limited mobility. Any volunteer who meets the exclusion criteria will be accommodated as best as possible (e.g. assignment to the most appropriate condition in the case of a physical disability) and their data will not be included in the analysis.

Recruitment

Participants will be recruited from the general community through a combination of existing collaborations with schools in the UK and Ireland. We will also recruit UK and Irish volunteers via online research platforms (e.g. Prolific; www.prolific.co) as well as extant panels of adolescent research volunteers via the MRC-Cognition and Brain Sciences Unit (University of Cambridge). Finally, and if necessary, we will also conduct a targeted recruitment campaign to recruit young people and schools using posters, pamphlets and online adverts on social media.

Sample size

The primary training outcome is self-rated decentering and this will be assessed using both a popular inventory of decentering (Experiences Questionnaire; EQ[8]) and ESM items. There is no consensus on how to best conduct a statistical power analysis for ESM data. Power and sample calculations were therefore based on the effect of training condition on EQ scores. A power analysis calculated in G*Power indicated that a total sample size of 90 is powered at 80% to observe a significant effect of training condition (decentering vs. control) on post-intervention decentering with a medium effect size of f = 0.3 (alpha = .05) after adjusting for baseline EQ-score. A medium effect size is plausible given that previous research reports a medium–large effect of extant psychological intervention on EQ scores. An attrition rate of around 20% is anticipated based on our previous research. Therefore, and with 57 participants in each group, the current study is adequately powered to observe a medium to large effect of decentering training on our candidate training outcome measures and to

For peer review only - http://bmjopen.bmj.com/site/about/guidelines.xhtml

determine the nature of this interaction. Regarding our secondary mental health outcomes (i.e. anxiety, depression and psychological wellbeing), a sample size of 57 per group is consistent with our previous research to evaluate the impact of novel psychological interventions. That is, such group sizes can provide a reasonable range of point estimates of the effect on mental health outcome measures that are sufficient to guide later research; for example, fifty-four participants per group is powered at 80% to observe a significant ($p < .05$) main effect of group (decentering versus active control) on baseline adjusted mental health outcomes with an effect size $f = 0.26$.

Intervention

Decentering training. A five week psychological decentering training programme was developed by MB, RK and TD, based on our previous protocol (Travers-Hill et al., 2017). This involves audio-recorded scripts and an accompanying work book that guides participants through four types of decentering technique; this structure is partly based on a recent taxonomy of self-distancing (a construct closely related to decentering)[29]. The decentering techniques include: (Week 1) *spatial distancing* wherein individuals are taught to re-imagine negative memories from a physically distant perspective (e.g. 'replay the memory but as if you're a fly on the wall'); (Week 2) *verbal distancing/cognitive defusion* wherein individuals are taught to re-phrase negative self-relevant statements in a way that challenges its literal value and influence over affective behavior (e.g. replacing first person pronouns with one's name); (Week 3) *temporal distancing* wherein individuals are taught to re-consider specific worries from a temporally distant future (e.g. 'how would this seem in 5 years?'); and (Week 4) *objective distancing* wherein individuals are taught to adopt a third-person perspective towards negative memories. Week 5 is a revision week. These techniques were selected since they are directly targeted at how adolescents

For peer review only

relate to and observe day-to-day psychological stressors. Specifically, the goal is to teach adolescents concrete ways to generate an objective (or distanced) self-perspective in response to everyday feelings, thoughts and memories that are unpleasant. We assume this training will develop participants' decentering ability above their baseline levels.

Each week will involve 5 audio-recorded exercises (10-15 minutes) that will be made available using common streaming services. One exercise will be posted each weekday, Monday to Friday. The first two exercises (Monday and Tuesday) are brief mindfulness grounding exercises designed to promote open monitoring of psychological experiences. The next three exercises (Wednesday to Friday) are decentering training exercises as described above. Adherence to the programme will be encouraged by directly contacting the participants in week 1 and week 3 to discuss their experiences so far and allow troubleshooting. Participant engagement will be monitored by reviewing the number of completed workbook exercise at the end of the five weeks. Participants will also complete daily diaries. These will include five questions about the intervention and its application, such as “Did you complete any of the programme exercises today?” or “During the day, were you able to apply the skills you've learned from the exercises?” (see Supplemental Materials). A participant's engagement in the trial will be discontinued if they elect to withdraw their participation or if they experience serious physical/mental health difficulties that necessitate medical or psychological intervention.

Physical and cognitive exercise. A five week active control programme was developed (MB, RK, and TD). This contains two elements that roughly match the decentering training for time and cognitive engagement. First, guided physical movement routines will be completed in lieu of mindfulness grounding exercises

For peer review only - http://bmjopen.bmj.com/site/about/guidelines.xhtml

(Monday-Tuesday). These movements are intended to emulate the physiological nature of grounding exercises but without an internal focus. Participants will watch short videos in which one member of the research team (RK) illustrates a basic series of body stretches. Accompanying audio provides additional direction and this was recorded using the same voices from the decentering training programme. Each video comprises 15 stretches, with each stretch held for 30 seconds and a 10 second break between stretches. The physical movements were selected for their ease and accessibility. Care was also taken to select movements that are safe and cater to a range of physical abilities.

Second, gamified versions of standard cognitive tasks will be completed in lieu of the decentering training (Wednesday-Friday). These games are intended to emulate the cognitive effort associated with decentering exercises. Each participant will have a link that allows them to select one of three games, which can be completed on a personal smart phone (for game outlines, see https://osf.io/aw6c5). Games include: (1) a Multi-Target Visual Search Task wherein participants search and respond to specific targets within a broader stimulus array; (2) a Go/NoGo Task wherein a speeded response is made in response to a 'go' signal but inhibited in response to a 'no go' signal: and (3) a Digit Recall Task wherein participants must recall a sequence on a number pad (i.e. a digit-span task). Adherence to the programme will be encouraged by contacting the participants in week 1 and week 3 to discuss their experiences. Engagement will be monitored by reviewing task completion rates and performance measures (response times and accuracy) at the end of the five weeks.

Outcomes

There are two types of outcome measures in this study (see Figure 1 for Timeline). These include measures of (1) decentering and (2) mental health. We will

For peer review only

also examine if there are any far-transfer effects of decentering training by including measures of (3) cognitive performance. Figure 1 provides a schematic overview of the assessment timeline.

Decentering outcome measures.

The primary training outcome measure is self-rated decentering as measured using the experiences questionnaire (EQ). A secondary decentering outcome measure is (2) the self-rated use of psychological decentering in response to psychological stressors as measured using ESM. These measures have been included to address research questions 1a and 1b, respectively.

Experiences questionnaire. This widely-used self-report assessment features 11 items that explore an individual's tendency to psychologically decenter from difficult subjective experiences in day-to-day life (e.g. 'I can observe unpleasant feelings without being drawn into them')[8]. Items are answered using a 5-point Likert scale from 1 = 'Never' to 5 = 'All the time'). Scores on the EQ are positively associated with other decentering-related constructs like experiential avoidance (a tendency to attempt to evade difficult thoughts and feelings)[30-34] and cognitive reappraisal (an ability to reconceptualise situations to modify emotional impact)[27,35]. Finally, EQ scores improve over the course of behavioural and cognitive therapies; the magnitude of such change is associated with key outcomes like symptom severity and quality-of-life[23,26,27,36,37]. The EQ will be administered at baseline, mid-intervention and post-intervention. We expect mean EQ scores will increase in the decentering training group relative to the active control condition.

ESM decentering items. Two ESM items have been developed to estimate the momentary use of decentering in response to psychological stressors like difficult feelings, memories or thoughts. These are "*Since the last beep, I was able to distance*

For peer review only

myself from unpleasant feelings" and "*Since the last beep, I was able to distance myself from unpleasant things on my mind*". Items are answered using a 7-point Likert scale ranging from 1 = 'Not at all' to 7 = 'Very much'). ESM items will be delivered four times daily across a five day ESM baseline period and the five weeks of training (see Figure 1). All ESM items are described in Table 1 (see overleaf). We expect momentary reports of decentering to increase over time in the decentering training group relative to the active control condition.

Secondary mental health outcomes.

Our secondary mental health outcomes are (1) self-rated anxiety, depression and psychological well-being and (2) self-rated emotional reactivity following a negative mental experience (e.g. unpleasant thought, feeling or memory) as measured using ESM. These measures have been included to address research questions 2a and 2b.

Mental health symptoms. This study will explore the potential impact of decentering training on mental health outcomes at baseline, mid-intervention and post-intervention. Previous findings suggest that decentering is negatively associated with anxiety and depression [14-16] as well as feelings of anger in adolescents[38,39]. We will therefore include measures of each of these difficulties. Anxiety symptoms will be assessed using the Revised Child Anxiety and Depression Scale-Short Version (RCADS-15)[40]. The RCADS-SV is a 15 item scale measuring the reported frequency of various symptoms of anxiety and low mood. Depression symptoms will be assessed using the Centre for Epidemiology Depression Scale (CES-D)[41]. The CES-D is a 20 item inventory measuring depressive symptoms experienced in the past week.

Table 1. Experience Sampling Items

Item	Question	Rating	Outcome	Conditiional
1	Right now, I feel…	-5 = Very Negative, 5 = Very Positive	Affect	Always Shown
2	I Feel:		Feelings/Mood	Always Shown
i	*Happy*	1 = Not at all, 7 = Very Much	Feelings/Mood	Always Shown
ii	*Relaxed*	1 = Not at all, 7 = Very Much	Feelings/Mood	Always Shown
iii	*Satisfied*	1 = Not at all, 7 = Very Much	Feelings/Mood	Always Shown
iv	*Enthusiastic*	1 = Not at all, 7 = Very Much	Feelings/Mood	Always Shown
v	*Nervous*	1 = Not at all, 7 = Very Much	Feelings/Mood	Always Shown
vi	*Sad*	1 = Not at all, 7 = Very Much	Feelings/Mood	Always Shown
vii	*Irritated*	1 = Not at all, 7 = Very Much	Feelings/Mood	Always Shown
viii	*Stressed*	1 = Not at all, 7 = Very Much	Feelings/Mood	Always Shown
3	Since the last beep, I felt sucked in by negative feelings?	1 = Not at all, 7 = Very Much	ER | Immersion negative feelings	Always Shown
4	Since the last beep, I was able to distance myself from negative feelings?	1 = Not at all, 7 = Very Much	ER | Distance negative feelings	Always Shown
5	Since the last beep, I tried to distract myself from negative feelings?	1 = Not at all, 7 = Very Much	ER | Distraction	Always Shown
6	Since the last beep, I noticed an unpleasant thought or memory?	1 = Not at all, 7 = Very Much	Thoughts/memories	Always Shown
7	Since the last beep, I was upset by an unpleasant thought or memory?	1 = Not at all, 7 = Very Much	Thoughts/memories	Always Shown
8	The unpleasant thing on my mind was about …	Myself, Others, A combination of these, None of these	Content- self/other	Contingent | (if Q6 != 1)
9	The unpleasant thing on my mind was about …	Social things, Professional things, A combination of these, None of these	Content- social/non-social	Contingent | (if Q6 != 1)
10	The unpleasant thing on my mind was about …	The past, The present, The future, A combination, None of these	Content- past/future	Contingent | (if Q6 != 1)
12	Since the last beep, I felt sucked in by unpleasnt things on my mind.	1 = Not at all, 7 = Very Much	ER | Immersion in thoughts/memories	Always Shown
13	Since the last beep, I able to distance myself from unpleasant things on my mind.	1 = Not at all, 7 = Very Much	ER | Distance from thoughts/memories	Always Shown
14	Since the last beep, I tried to think differently about things so to feel better.	1 = Not at all, 7 = Very Much	ER | Re-appraisal	Always Shown
15	Since the last beep, I tried to distract myself from unpleasent things in my mind.	1 = Not at all, 7 = Very Much	ER | Distract	Always Shown
16	Since the last beep, I feel I benefited from the daily programme.	1 = Not at all, 7 = Very Much	Usefulness	Always Shown
Branch 1	Right now, I am …	Indoors, Outdoors	Branch	Contingent | (if Q6 == 1)
Branch 2	Right now, I am …	Alone, With others	Branch	Contingent | (if Q6 == 1)
Branch 5	Since the last beep, I felt social.	1 = Not at all, 7 = Very Much	Branch	Contingent | (if Q6 == 1)

Anger will be measured using the State-Trait Anger Expression Inventory-2 Child and Adolescent (STAXI-2)[42], which is a 35 item self-report measuring anger expression and control in adolescents.

Two measures will also be included to estimate the impact of decentering training on psychosocial strengths/difficulties and general wellbeing. The Strengths and Difficulties Questionnaire (SDQ) is a screening tool with 5 subscales (Emotional Symptoms, Conduct Problems, Hyperactivity/Inattention, Peer Relationships, and Prosocial Behaviour) as well as an impact supplement to estimate psycho-social functioning. This tool was included to capture behavioural symptoms such as conduct difficulties and hyperactivity. Psychological wellbeing will be measured using the Warwick-Edinburgh Mental Wellbeing Scale (WEMWBS; Tennant et. al, 2007). The WEMWBS is a 14 item measure of positive mental health including positive affect, functioning and inter-personal relationships. Measures will be administered at baseline, mid-intervention and post-intervention.

ESM emotional reactivity. This study will examine if the lagged effect of the decentering from difficult thoughts, feelings or memories (time = n) on later affect (time = $n + 1$) changes across training. One ESM items will capture general affect: 'Right now I feel…', answered on a 11 point Likert scale ranging from -5 = Very negative to +5 =Very positive. Two separate ESM items will capture the self-initiated use of decentering skills in response to negative mental experiences (Table **1**. These are: 'Since the last message, I was able to distance myself from [a] a negative feeling *or* [b] unpleasant things on my mind'. Additional ESM items will check for the occurrence of negative mental experiences. These include checks of (a) negative feelings by asking '*I feel Nervous/Sad/Irritated/Stressed*' and (b) unpleasant cognitive events by

For peer review only - http://bmjopen.bmj.com/site/about/guidelines.xhtml

asking '*Since the last beep, I was upset by a thought or memory*'. This will be scored on a 7 point Likert scale from 1 = Not at all to 7 = Very Much.

Additional self-rated assessments

Participants will complete several additional self-rated assessments for the purpose of exploratory analysis. Participants will also complete a brief end of day questionnaire that will include a measure of mood, assessing presence and duration of different mood states. Nine moods will be investigated– happy, lively, content, satisfied, depressed, bored, anxious, irritable and tense. These items will allow for exploratory analysis of how individuals' moods may relate to the two experimental conditions. The end of day questionnaire will also include items investigating whether participants have completed any exercises from their programme (decentering or control), how difficult these exercises were to complete, and whether these skills were relevant to their everyday life. This section is included both as a measure of adherence to the programme, and as a broad measure of participant feedback.

Our team is currently developing an adolescent friendly self-rated decentering inventory. A provisional version of this scale will be included at pre- and post-intervention as part of its on-going validation. We are also investigating the relationship between decentering and other emotion regulation skills. We will therefore include: the Cognitive Emotion Regulation Questionnaire (CERQ)[43], which is a 36 item questionnaire cataloguing a range of emotion regulation strategies; the Difficulties in Emotion Regulation Scale (DERS)[44], which is a 36-item questionnaire investigating problems with emotion regulation, including 6 subscales: non-acceptance of emotional responses; difficulty in engaged in goal-directed behaviour; impulse control difficulties; lack of emotional awareness; limited access to emotion regulation strategies; and lack of emotional clarity: and the Child and Adolescent Mindfulness Measure (CAMM),

For peer review only

which is a 10 item measure exploring trait mindfulness in children and adolescents. Participants will complete the Coronavirus Anxiety Scale[45] scales at baseline and post-intervention. This may help us address any unexpected impact of the COVID-19 pandemic on the intervention. To control for impact of patient expectation on intervention efficacy, a single item patient expectation scale will also be administered at baseline.

Far transfer effects.

This study will include performance-based tasks to explore potential far-transfer effects of decentering training on cognition (research question 3). These include (1) affective cognitive control and (2) self-perspective processing. These measures will be administered at baseline and post-intervention.

Affective cognitive control. This study will explore if decentering training influences the ability to maintain cognitive performance within emotional contexts (i.e. affective cognitive control). This can be estimated via the Emotional Stop Signal Task (ESST; Figure S1)[46]. On each trial, a neutral or negative valence image is presented before a 'go-signal' that requires a speeded button-press (75% of trials). Participants must quickly make the appropriate button-press unless the 'go-signal' is followed by a 'stop-signal (25%). Participants must withhold their response on these trials. The period between the go and stop-signal (stop signal delay; SSD) varies over the task. Following a successful stop trial, the task is made more difficult by increasing the SSD by 25 ms. Following an unsuccessful stop trial, the task is made easier by decreasing the SSD by 25 m. This tracking algorithm typically causes a failure to stop on ~50% of trials. Sustained attention in emotional and non-emotional contexts will be estimated by calculating (1) the reaction time (RT) mean on go-trials and (2) the inter-trial variability coefficient (i.e. the RT mean divided by the RT standard deviation). Inhibitory

control in emotional and non-emotional contexts will estimated by calculating the *stop signal reaction time (i*.e. mean RT – mean SSD).

Self-referential processing. This study will explore if decentering training influences the ability to flexibly shift one's self-perspective. This can be indexed using the own-body transformation task (OBT; Figure S2)[47]. On each trial, a schematic human figure is presented that face either with their front to the participant or their back to the participant. This figure also holds a black glove in one hand (e.g. left). Participants are instructed to imagine themselves in the body position of the figure and then judge whether the glove is held by the figures left of right hand. Performance is compared to a within-subject control task wherein participants judge the position of the glove without altering their self-representation. The outcome variables are mean RT and the percentage error rate. These are found to increase as a function of the self-perspective manipulation; that is, they are higher for front-facing characters.

Methods and Procedures

Assignment of interventions

Allocation. Following baseline assessment, participants will be stratified according to sex and depression severity (using the CES-D). They will then be randomly assigned to either the decentering training programme or the active control condition. This will be managed by the trial statistician (PW) using a minimization procedure. Participant allocation will be then shared with the research coordinator responsible for providing appropriate access links for the online intervention (MB and RK).

Blinding. Research coordinators (MB and RK) will be blinded during assessment, data pre-processing and statistical analysis. Due to the nature of the study, it is not possible to remain blinded during participant allocation. This is because

For peer review only

each participant needs to be given a secure link to the online training programme, either decentering training or the active control. All outcome assessments will be completed online; participants will access the same link to complete online assessments, regardless of group. Thus, there are no in-person data collectors with an awareness of participants' allocation. Initial data will be pre-processed (e.g. calculation of summary scores like means, percentage accuracy, sums etc.). Pre-processing will be completed using pre-existing scripts coded in R and MATLAB (MB). At this point, the dataset will be shared with the trial statistician (PW) who will conduct the statistical analysis and who will be blind to participant allocation. Once the analysis is complete, the trial coordinators will un-blind the dataset by indicating which participants were allocated to which group.

Consent and data collection procedures

Recruited volunteers will complete an eligibility check during an initial phone call. Volunteers who are eligible will be invited to participate in the study. Written informed consent will be sought after an initial briefing that explains the data collection protocol. This briefing will also include a tutorial on downloading and using the experience sampling app (PsyMate2; www.psymate.eu). Participants will also have the opportunity to ask any questions. Once consent is provided, participants will be given an anonymous trial identification code and added to an encrypted list for allocation. Participants will also receive a URL link to complete baseline measures. Figure 1 (see overleaf) illustrates the full schedule for outcome measures. The two-week ESM baseline assessment will also begin. At the end of this period, participants will receive a tutorial on accessing the intervention materials.

Over five weeks, participants in both interventions will access and rehearse exercises (Monday-Friday). Decentering training exercises are based on audio files

For peer review only

to be delivered via their preferred music/post-streaming services (e.g. Spotify/Podcast Addict).

For peer review only

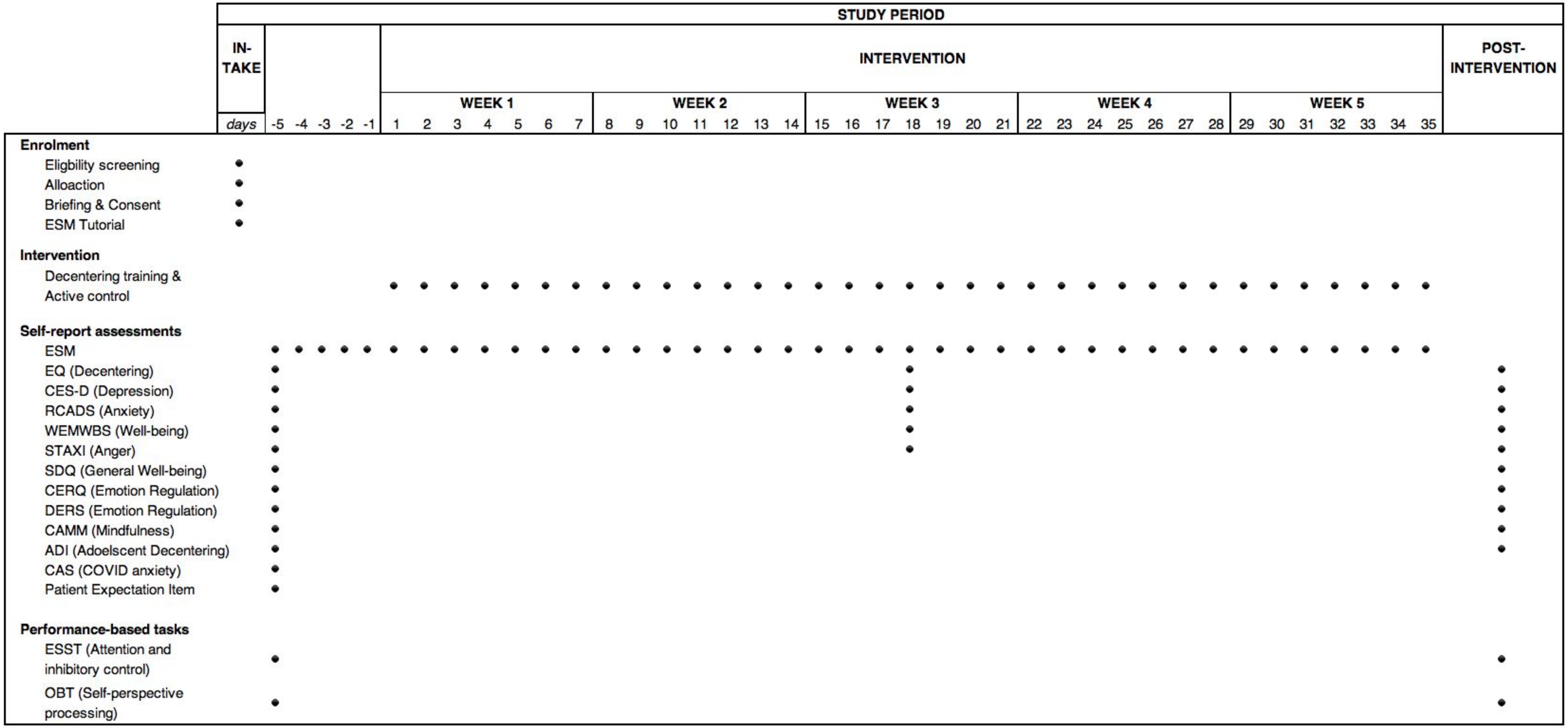

Figure 1: A schematic overview of the participant assessment time line. The aim is to administer self-report assessments and performance-based tasks within two-days of the above guide times.

The active control exercises are based on YouTube videos posted on a Private Channel. Each day, the PsyMate2 App will prompt participants to complete their daily exercise as well as complete any required ESM items. These will be sent four times per day. A brief survey will also be sent through the PsyMate2 App each evening to ask whether participants completed their daily exercises, and if so, to ask about their experiences as well as questions on their mood states (Supplemental Materials). At the end of Week 5, participants will be asked to complete the online assessment battery. Participants will then complete a debriefing session. During the debriefing session they will be given the opportunity to provide feedback on their experience of the study, and to ask any questions they may have. They will also be given a list of relevant mental health resources should they feel the need to access them.

Participant Retention & Follow Up. Retention will be promoted in a number of ways. Firstly, participants will not be paid until receipt of their completed post-intervention assessment battery. Secondly, participants will be made aware that they can receive a bonus payment of £50 if they complete all of their assessments and daily diaries. Thirdly, telephone contact will be made with participants during week 1 and 3 to allow any troubleshooting and ensure that participants are filling in diaries and experience sampling appropriately. Telephone contact will also be made with a participant should they fail to complete mid-intervention assessments (Week 3).

Data management and statistical analysis

Data confidentiality and management

All participants will be given an anonymous trial identification code to use when submitting their completed assessments. A file linking the participant's name to their identification code will be saved in an encrypted and password protected file and stored on a secure, protected server within the MRC-Cognition and Brain Sciences

For peer review only - http://bmjopen.bmj.com/site/about/guidelines.xhtml

Unit. This file will be deleted once the final round of data collection is complete. Outcome data will be gathered remotely using online platforms (e.g. Qualtrics; www.qualtrics.com) and an ESM data collection app. During periods of data collection, these data will be transferred to in-house secure servers within the MRC-Cognition and Brain Sciences Unit, University of Cambridge. The source data will be then deleted. Access to these data will be restricted to primary research team members (MB, RK, TD). Data management will be overseen by these team members. There will be no formal Data Management Committee (DMC) because it is a small scale trial.

Statistical analysis

Self-rated and performance-based measures

The statistical analysis will be conducted by the trial statistician (PW) who will be blind to training condition. The analysis will be conducted on an intention-to-treat basis and a similar initial analysis is planned for (i) the primary training outcome (i.e. self-rated EQ scores) and (ii) secondary mental health outcomes (i.e. CES-D, RCADS, STAXI-2, SDQ and WEMWBS). Analysis of covariance (ANCOVA) will be calculated to estimate the effect of training condition on outcome measures after adjusting for baseline performance and grouping stratification variables. This model will be calculated for our primary end-point, i.e. post-intervention (i.e. post-intervention outcome adjusted for baseline score). This model will additionally be calculated for our

For peer review only - http://bmjopen.bmj.com/site/about/guidelines.xhtml

mid-intervention time point (i.e. mid-intervention outcome adjusted for baseline score) and post-intervention. There are no planned interim-analysis. If a subset of items of a measure (≤ 20% within-measure missing data), a total score will be calculated using the mean score across the non-missing items; an approach we have reported elsewhere (www.osf.io/d6y9q). Otherwise, the score for that measure will be noted as missing. We will assume that data will be missing at random and address this using standard imputation approaches in R (R Core Team, 2013).

ESM analysis

Only participants who completed at least 33% of the ESM questionnaires will be included in the analyses, in line with ESM guidelines[48]. Statistical analyses will be performed using hierarchical linear models. These models are especially suited to deal with dependency inherent in the data due their hierarchical structure: ESM observations (level 1) nested within individuals (level 2). Potential confounders will be included in the model as covariates. First, a linear growth model will be estimated to assess the effect of condition (decentering training vs. active control) on decentering skills over time (i.e., the duration of the intervention). Second, time-lagged analyses will be performed to evaluate if higher self-rated decentering skills when confronted with a momentary psychological stressor (time = n) predict significantly lower negative affect ratings at a later time point (time = n + 1). We will also control for momentary negative affect ratings (at time = n). Random intercepts and random slopes models will be estimated. In hierarchical linear models, the fixed effects reflect the overall

For peer review only - http://bmjopen.bmj.com/site/about/guidelines.xhtml

association between a predictor and the outcome of interest, whereas the random effects reflect individual differences in this association.

Ethics and dissemination

Ethical approval and protocol amendments.

This study was approved by the Cambridge Psychology Research Ethics Committee, University of Cambridge (PRE.2019.109). Approval for any protocol amendments will be sought by this committee. This includes methodological and/or trial management amendments. The online registration of the trial will also be updated in this situation and any amendments will be outlined in the trial manuscripts. This committee will also be contacted should we encounter any unexpected ethical concerns.

Dissemination

The findings of this study will be disseminated through typical academic routes including poster/paper presentations at (inter)-national conferences, academic institutes and through publication in peer-reviewed journals. Given that some research questions are less exploratory than others, two key manuscripts are planned for the trial data. The first will describe the impact of training on self-rated decentering and mental health outcomes. The second will describe the exploratory research around the impact on cognitive performance measures. Findings will also be disseminated to the broader public through seminars and workshops with relevant stakeholders as well as online blogs and podcasts. These manuscripts will be completed and submitted for review irrespective of the nature of the observed effects. Following the publication of the first manuscript, a fully anonymized dataset will be made available online on open-access databases whose servers are located within the UK or Europe. Data processing scripts and programming codes will also be made available online.

For peer review only

Patient and Public Involvement

A youth advisory panel was formed from May to December 2020. This panel advised on the accessibility of self-rated measures of decentering. They also provided qualitative feedback on decentering as an emotional regulation stratagem and the potential for decentering training. This took place across several informal focus groups (see, Bennett et al., 2021 for more information). During study development, patients and the public were not involved in the choice of outcome measures, or recruitment for the study.

Participants of the study will be given opportunity to give feedback in person or anonymously at debrief, so as to improve the protocol for future trials.

Discussion and Future Research

Decentering is a helpful strategy to notice and interact with unpleasant inner experiences. Evidence suggests that the ability to use this strategy is continuously distributed in the population, with those at the higher end of the continuum reporting less anxiety and depression. Decentering also appears to be a malleable skill that is reinforced across a range of different psychological interventions. Selectively training this skill during adolescence might therefore delimit the negative impact of everyday psychological stressors. This has the potential to improve emotional wellbeing in the short term and mitigate anxiety and depression symptoms in the long-term. The current trial is an initial investigation to establish whether decentering skills can be selectively trained during adolescence and to characterise its impact on mental health outcomes. These findings may not only reveal some of the best ways to teach this skill but may provide estimates of effect sizes that can inform future research.

For peer review only

References

1 Kessler, R. C. *et al.* Age of onset of mental disorders: a review of recent literature. *Current opinion in psychiatry* 20, 359 (2007).

2 Wilde, S. *et al.* Mindfulness training in UK secondary schools: a multiple case study approach to identification of cornerstones of implementation. *Mindfulness* 10, 376-389 (2019).

3 Vostanis, P., Humphrey, N., Fitzgerald, N., Deighton, J. & Wolpert, M. How do schools promote emotional well-being among their pupils? Findings from a national scoping survey of mental health provision in E nglish schools. *Child and Adolescent Mental Health* 18, 151-157 (2013).

4 Weare, K. & Nind, M. Mental health promotion and problem prevention in schools: what does the evidence say? *Health promotion international* 26, i29-i69 (2011).

5 Teasdale, J. D. *et al.* Metacognitive awareness and prevention of relapse in depression: empirical evidence. *Journal of consulting and clinical psychology* 70, 275 (2002).

6 Bernstein, A. *et al.* Decentering and related constructs: A critical review and metacognitive processes model. *Perspectives on Psychological Science* 10, 599-617 (2015).

7 Bennett, M. P. *et al.* Decentering as a core component in the psychological treatment and prevention of youth anxiety and depression: A narrative review and insight report. *Translational Psychiatry* (2021).

8 Fresco, D. M. *et al.* Initial psychometric properties of the experiences questionnaire: validation of a self-report measure of decentering. *Behavior therapy* 38, 234-246 (2007).

For peer review only

9 Ayduk, Ö. & Kross, E. From a distance: implications of spontaneous self-distancing for adaptive self-reflection. *Journal of personality and social psychology* 98, 809 (2010).

10 White, R. E., Kross, E. & Duckworth, A. L. Spontaneous self-distancing and adaptive self-reflection across adolescence. *Child development* 86, 1272-1281 (2015).

11 Masuda, A., Hayes, S. C., Sackett, C. F. & Twohig, M. P. Cognitive defusion and self-relevant negative thoughts: Examining the impact of a ninety year old technique. *Behaviour research and therapy* 42, 477-485 (2004).

12 Masuda, A. *et al.* A parametric study of cognitive defusion and the believability and discomfort of negative self-relevant thoughts. *Behavior modification* 33, 250-262 (2009).

13 Nook, E. C., Vidal Bustamante, C. M., Cho, H. Y. & Somerville, L. H. Use of linguistic distancing and cognitive reappraisal strategies during emotion regulation in children, adolescents, and young adults. *Emotion* 20, 525 (2020).

14 Greco, L. A., Lambert, W. & Baer, R. A. Psychological inflexibility in childhood and adolescence: development and evaluation of the Avoidance and Fusion Questionnaire for Youth. *Psychological assessment* 20, 93 (2008).

15 Livheim, F. *et al.* Psychometric properties of the Avoidance and Fusion Questionnaire for Youth: A psychological measure of psychological inflexibility in youth. *Journal of Contextual Behavioral Science* 5, 103-110 (2016).

16 Muris, P. *et al.* Inflexible youngsters: Psychological and Psychopathological Correlates of the Avoidance and Fusion Questionnaire for youths in nonclinical Dutch adolescents. *Mindfulness* 8, 1381-1392 (2017).

For peer review only

17 Valdivia-Salas, S., Martín-Albo, J., Zaldivar, P., Lombas, A. S. & Jiménez, T. I. Spanish validation of the Avoidance and Fusion Questionnaire for Youth (AFQ-Y). *Assessment* 24, 919-931 (2017).

18 Teasdale, J. D., Segal, Z. & Williams, J. M. G. How does cognitive therapy prevent depressive relapse and why should attentional control (mindfulness) training help? *Behaviour Research and therapy* 33, 25-39 (1995).

19 Zettle, R. D., Rains, J. C. & Hayes, S. C. Processes of change in acceptance and commitment therapy and cognitive therapy for depression: A mediation reanalysis of Zettle and Rains. *Behavior modification* 35, 265-283 (2011).

20 Eustis, E. H., Hayes-Skelton, S. A., Orsillo, S. M. & Roemer, L. Surviving and thriving during stress: a randomized clinical trial comparing a brief web-based therapist-assisted acceptance-based behavioral intervention versus waitlist control for college students. *Behavior therapy* 49, 889-903 (2018).

21 Arch, J. J., Wolitzky-Taylor, K. B., Eifert, G. H. & Craske, M. G. Longitudinal treatment mediation of traditional cognitive behavioral therapy and acceptance and commitment therapy for anxiety disorders. *Behaviour research and therapy* 50, 469-478 (2012).

22 Twohig, M. P. *et al.* A randomized clinical trial of acceptance and commitment therapy versus progressive relaxation training for obsessive-compulsive disorder. *Journal of consulting and clinical psychology* 78, 705 (2010).

23 Bieling, P. J. *et al.* Treatment-specific changes in decentering following mindfulness-based cognitive therapy versus antidepressant medication or placebo for prevention of depressive relapse. *Journal of consulting and clinical psychology* 80, 365 (2012).

For peer review only

24 Josefsson, T., Lindwall, M. & Broberg, A. G. The effects of a short-term mindfulness based intervention on self-reported mindfulness, decentering, executive attention, psychological health, and coping style: examining unique mindfulness effects and mediators. *Mindfulness* 5, 18-35 (2014).

25 Orzech, K. M., Shapiro, S. L., Brown, K. W. & McKay, M. Intensive mindfulness training-related changes in cognitive and emotional experience. *The Journal of Positive Psychology* 4, 212-222 (2009).

26 Hayes-Skelton, S. A., Calloway, A., Roemer, L. & Orsillo, S. M. Decentering as a potential common mechanism across two therapies for generalized anxiety disorder. *Journal of consulting and clinical psychology* 83, 395 (2015).

27 Hayes-Skelton, S. A. & Graham, J. R. Decentering as a common link among mindfulness, cognitive reappraisal, and social anxiety. *Behavioural and cognitive psychotherapy* 41, 317 (2013).

28 Travers-Hill, E., Dunn, B. D., Hoppitt, L., Hitchcock, C. & Dalgleish, T. Beneficial effects of training in self-distancing and perspective broadening for people with a history of recurrent depression. *Behaviour research and therapy* 95, 19-28 (2017).

29 Powers, J. P. & LaBar, K. S. Regulating emotion through distancing: A taxonomy, neurocognitive model, and supporting meta-analysis. *Neuroscience & Biobehavioral Reviews* 96, 155-173 (2019).

30 Gregório, S., Pinto-Gouveia, J., Duarte, C. & Simões, L. Expanding research on decentering as measured by the Portuguese version of the experiences questionnaire. *The Spanish journal of psychology* 18, E23 (2015).

31 Naragon-Gainey, K. & DeMarree, K. G. Structure and validity of measures of decentering and defusion. *Psychological Assessment* 29, 935 (2017).

For peer review only

32 Lucena-Santos, P., Carvalho, S., Pinto-Gouveia, J., Gillanders, D. & Oliveira, M. S. Cognitive Fusion Questionnaire: Exploring measurement invariance across three groups of Brazilian women and the role of cognitive fusion as a mediator in the relationship between rumination and depression. *Journal of Contextual Behavioral Science* 6, 53-62 (2017).

33 Soler, J. *et al.* Assessing decentering: validation, psychometric properties, and clinical usefulness of the experiences questionnaire in a Spanish sample. *Behavior therapy* 45, 863-871 (2014).

34 Forman, E. M. *et al.* The Drexel defusion scale: A new measure of experiential distancing. *Journal of Contextual Behavioral Science* 1, 55-65 (2012).

35 Kobayashi, R., Shigematsu, J., Miyatani, M. & Nakao, T. Cognitive reappraisal facilitates decentering: A longitudinal cross-lagged analysis study. *Frontiers in psychology* 11, 103 (2020).

36 Hayes-Skelton, S. A. & Marando-Blanck, S. Examining the interrelation among change processes: Decentering and anticipatory processing across cognitive behavioral therapy for social anxiety disorder. *Behavior therapy* 50, 1075-1086 (2019).

37 Hayes-Skelton, S. A. & Lee, C. S. Changes in decentering across cognitive behavioral group therapy for social anxiety disorder. *Behavior therapy* 49, 809-822 (2018).

38 Mischkowski, D., Kross, E. & Bushman, B. J. Flies on the wall are less aggressive: Self-distancing "in the heat of the moment" reduces aggressive thoughts, angry feelings and aggressive behavior. *Journal of Experimental Social Psychology* 48, 1187-1191 (2012).

For peer review only

39 Kross, E., Duckworth, A., Ayduk, O., Tsukayama, E. & Mischel, W. The effect of self-distancing on adaptive versus maladaptive self-reflection in children. *Emotion* 11, 1032 (2011).

40 Ebesutani, C. *et al.* The Revised Child Anxiety and Depression Scale-Short Version: Scale reduction via exploratory bifactor modeling of the broad anxiety factor. *Psychological assessment* 24, 833 (2012).

41 Radloff, L. S. The CES-D scale: A self-report depression scale for research in the general population. *Applied psychological measurement* 1, 385-401 (1977).

42 Brunner, T. M. & Spielberger, C. D. *STAXI-2 C/A: State-Trait Anger Expression Inventory-2, Child and Adolescent: Professional Manual*. (Psychological Assessment Resources, 2009).

43 Garnefski, N. & Kraaij, V. The cognitive emotion regulation questionnaire. *European Journal of Psychological Assessment* 23, 141-149 (2007).

44 Gratz, K. L. & Roemer, L. Multidimensional assessment of emotion regulation and dysregulation: Development, factor structure, and initial validation of the difficulties in emotion regulation scale. *Journal of psychopathology and behavioral assessment* 26, 41-54 (2004).

45 Silva, W. A. D., de Sampaio Brito, T. R. & Pereira, C. R. COVID-19 anxiety scale (CAS): Development and psychometric properties. *Current Psychology*, 1-10 (2020).

46 Verbruggen, F. & De Houwer, J. Do emotional stimuli interfere with response inhibition? Evidence from the stop signal paradigm. *Cognition and emotion* 21, 391-403 (2007).

For peer review only

47 Blanke, O. *et al.* Linking out-of-body experience and self processing to mental own-body imagery at the temporoparietal junction. *Journal of Neuroscience* 25, 550-557 (2005).

48 Delespaul, P. A. Assessing schizophrenia in daily life: The experience sampling method. (1995).

For peer review only

Author Statement

- MB & RK – Substantial contributions to the conception, design of work, data acquisition, data analysis and interpretation, and manuscript preparation.
- TD, TF, WK, SJB, MW, GW – Substantial contributions to the conception and design of work.
- AAB, TS, DD, ED, PW, MV, BL, HC – Substantial contributions to data acquisition & analysis.

For peer review only

Appendix A

MRC Cognition and Brain Sciences Unit

UNIVERSITY OF CAMBRIDGE

Tim Dalgleish
Principal Investigator
MRC Cognition and Brain Sciences Unit
University of Cambridge
15 Chaucer Road,
Cambridge, CB2 7EF
Phone: 01223 766166
myriad@mrc-cbu.cam.ac.uk

Participant Consent Form

Study title: Investigating the neuro-cognitive dynamics of decentring training and its impact on emotional reactivity: A Longitudinal and multimodal assessment.

We are interested in how new therapeutic techniques like mindfulness and decentring affect your thoughts and feelings.

This study has been reviewed by the Cambridge Psychology Research Ethics Committee [PRE.2019.036]

Please initial at the end of each line and then sign below to indicate that you agree to take part in the study:

• Read and understood the information sheet for this study

……………

• Had the opportunity to consider the information and ask questions via the email address or

phone number given at the top of this sheet

……………

• Had your questions answered satisfactorily

……………

• Understood that this project will require you to have an app installed on your smartphone for the

duration of the project

……………

• Understood that your participation is voluntary and that he or she can withdraw at any time

without giving a reason

……………

For peer review only

• Understood that your data and personal details will be stored on secure servers. At the end of the study, the data will be transferred and held at The MRC Cognition and Brain Sciences Unit in a secure database and will be accessed by the academic community

………………

• The MRC Cognition and Brain Sciences Unit will take all reasonable steps to protect your privacy. Your data will be coded and will not be associated with your name or information that could reveal your identity. This anonymised data will be made freely available to the wider academic community ……………

• Understood that all information will be treated as strictly confidential and handled in accordance with the provisions of the Data Protection Act 2018

……………

• Understood that in all instances where the results are disseminated they will remain anonymous

……………

PLEASE USE BLOCK CAPITAL LETTERS WHEN FILLING IN THIS FORM

Your name……

Your school/college…..………

Gender ………………………. Year group………………………… Date of birth………………………………………..

Other information. Does you have any condition that may affect your performance on the tasks? If so please state: ……………………………………………………………….

Would you like to be contacted in the future about participating in other studies (please circle)?
YES NO

Your email………

Please sign below to indicate that you agree to take part in the study:

Your signature: ……... Date: ....

For peer review only

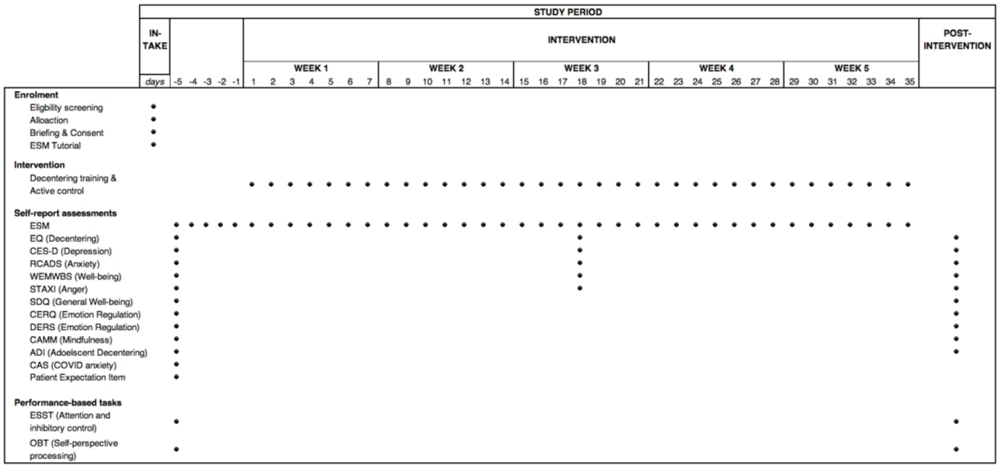

Figure 1 - A schematic overview of the participant assessment time line. The aim is to administer self-report assessments and performance-based tasks within two-days of the above guide times.

1108x516mm (38 x 38 DPI)

Table 1. Experience Sampling Items

Item	Question	Rating	Outcome	Conditiional
1	Right now, I feel…	-5 = Very Negative, 5 = Very Positive	Affect	Always Shown
2	I Feel:		Feelings/Mood	Always Shown
i	*Happy*	1 = Not at all, 7 = Very Much	Feelings/Mood	Always Shown
ii	*Relaxed*	1 = Not at all, 7 = Very Much	Feelings/Mood	Always Shown
iii	*Satisfied*	1 = Not at all, 7 = Very Much	Feelings/Mood	Always Shown
iv	*Enthusiastic*	1 = Not at all, 7 = Very Much	Feelings/Mood	Always Shown
v	*Nervous*	1 = Not at all, 7 = Very Much	Feelings/Mood	Always Shown
vi	*Sad*	1 = Not at all, 7 = Very Much	Feelings/Mood	Always Shown
vii	*Irritated*	1 = Not at all, 7 = Very Much	Feelings/Mood	Always Shown
viii	*Stressed*	1 = Not at all, 7 = Very Much	Feelings/Mood	Always Shown
3	Since the last beep, I felt sucked in by negative feelings?	1 = Not at all, 7 = Very Much	ER | Immersion negative feelings	Always Shown
4	Since the last beep, I was able to distance myself from negative feelings?	1 = Not at all, 7 = Very Much	ER | Distance negative feelings	Always Shown
5	Since the last beep, I tried to distract myself from negative feelings?	1 = Not at all, 7 = Very Much	ER | Distraction	Always Shown
6	Since the last beep, I noticed an unpleasant thought or memory?	1 = Not at all, 7 = Very Much	Thoughts/memories	Always Shown
7	Since the last beep, I was upset by an unpleasant thought or memory?	1 = Not at all, 7 = Very Much	Thoughts/memories	Always Shown
8	The unpleasant thing on my mind was about …	Myself, Others, A combination of these, None of these	Content- self/other	Contingent | (if Q6 != 1)
9	The unpleasant thing on my mind was about …	Social things, Professional things, A combination of these, None of these	Content- social/non-social	Contingent | (if Q6 != 1)
10	The unpleasant thing on my mind was about …	The past, The present, The future, A combination, None of these	Content- past/future	Contingent | (if Q6 != 1)
12	Since the last beep, I felt sucked in by unpleasnt things on my mind.	1 = Not at all, 7 = Very Much	ER | Immersion in thoughts/memories	Always Shown
13	Since the last beep, I able to distance myself from unpleasant things on my mind.	1 = Not at all, 7 = Very Much	ER | Distance from thoughts/memories	Always Shown
14	Since the last beep, I tried to think differently about things so to feel better.	1 = Not at all, 7 = Very Much	ER | Re-appraisal	Always Shown
15	Since the last beep, I tried to distract myself from unpleasent things in my mind.	1 = Not at all, 7 = Very Much	ER | Distract	Always Shown
16	Since the last beep, I feel I benefited from the daily programme.	1 = Not at all, 7 = Very Much	Usefulness	Always Shown
Branch 1	Right now, I am …	Indoors, Outdoors	Branch	Contingent | (if Q6 == 1)
Branch 2	Right now, I am …	Alone, With others	Branch	Contingent | (if Q6 == 1)
Branch 5	Since the last beep, I felt social.	1 = Not at all, 7 = Very Much	Branch	Contingent | (if Q6 == 1)

Table 1 - Experience Sampling Items

1028x501mm (38 x 38 DPI)

Reporting checklist for protocol of a clinical trial.

Based on the SPIRIT guidelines.

Instructions to authors

Complete this checklist by entering the page numbers from your manuscript where readers will find each of the items listed below.

Your article may not currently address all the items on the checklist. Please modify your text to include the missing information. If you are certain that an item does not apply, please write "n/a" and provide a short explanation.

Upload your completed checklist as an extra file when you submit to a journal.

In your methods section, say that you used the SPIRITreporting guidelines, and cite them as:

Chan A-W, Tetzlaff JM, Gøtzsche PC, Altman DG, Mann H, Berlin J, Dickersin K, Hróbjartsson A, Schulz KF, Parulekar WR, Krleža-Jerić K, Laupacis A, Moher D. SPIRIT 2013 Explanation and Elaboration: Guidance for protocols of clinical trials. BMJ. 2013;346:e7586

		Reporting Item	Page Number
**Administrative information**			
Title	#1	Descriptive title identifying the study design, population, interventions, and, if applicable, trial acronym	1
Trial registration	#2a	Trial identifier and registry name. If not yet registered, name of intended registry	1
Trial registration: data set	#2b	All items from the World Health Organization Trial Registration Data Set	1
Protocol version	#3	Date and version identifier	1
Funding	#4	Sources and types of financial, material, and other support	1
Roles and responsibilities: contributorship	#5a	Names, affiliations, and roles of protocol contributors	32

For peer review only

Roles and responsibilities: sponsor contact information	#5b	Name and contact information for the trial sponsor	1
Roles and responsibilities: sponsor and funder	#5c	Role of study sponsor and funders, if any, in study design; collection, management, analysis, and interpretation of data; writing of the report; and the decision to submit the report for publication, including whether they will have ultimate authority over any of these activities	N/A - Study Sponsor and Funders had no role
Roles and responsibilities: committees	#5d	Composition, roles, and responsibilities of the coordinating centre, steering committee, endpoint adjudication committee, data management team, and other individuals or groups overseeing the trial, if applicable (see Item 21a for data monitoring committee)	N/A - No Data Monitoring Committee was used
**Introduction**			
Background and rationale	#6a	Description of research question and justification for undertaking the trial, including summary of relevant studies (published and unpublished) examining benefits and harms for each intervention	1
Background and rationale: choice of comparators	#6b	Explanation for choice of comparators	12
Objectives	#7	Specific objectives or hypotheses	6
Trial design	#8	Description of trial design including type of trial (eg, parallel group, crossover, factorial, single group), allocation ratio, and framework (eg, superiority, equivalence, non-inferiority, exploratory)	7
**Methods: Participants, interventions, and outcomes**			

For peer review only

Study setting	#9	Description of study settings (eg, community clinic, academic hospital) and list of countries where data will be collected. Reference to where list of study sites can be obtained	8
Eligibility criteria	#10	Inclusion and exclusion criteria for participants. If applicable, eligibility criteria for study centres and individuals who will perform the interventions (eg, surgeons, psychotherapists)	8
Interventions: description	#11a	Interventions for each group with sufficient detail to allow replication, including how and when they will be administered	10
Interventions: modifications	#11b	Criteria for discontinuing or modifying allocated interventions for a given trial participant (eg, drug dose change in response to harms, participant request, or improving / worsening disease)	11
Interventions: adherance	#11c	Strategies to improve adherence to intervention protocols, and any procedures for monitoring adherence (eg, drug tablet return; laboratory tests)	11
Interventions: concomitant care	#11d	Relevant concomitant care and interventions that are permitted or prohibited during the trial	8
Outcomes	#12	Primary, secondary, and other outcomes, including the specific measurement variable (eg, systolic blood pressure), analysis metric (eg, change from baseline, final value, time to event), method of aggregation (eg, median, proportion), and time point for each outcome. Explanation of the clinical relevance of chosen efficacy and harm outcomes is strongly recommended	13
Participant timeline	#13	Time schedule of enrolment, interventions (including any run-ins and washouts), assessments, and visits for participants. A schematic diagram is highly recommended (see Figure)	18
Sample size	#14	Estimated number of participants needed to achieve study objectives and how it was	9

For peer review only

		determined, including clinical and statistical assumptions supporting any sample size calculations	
Recruitment	#15	Strategies for achieving adequate participant enrolment to reach target sample size	9
**Methods: Assignment of interventions (for controlled trials)**			
Allocation: sequence generation	#16a	Method of generating the allocation sequence (eg, computer-generated random numbers), and list of any factors for stratification. To reduce predictability of a random sequence, details of any planned restriction (eg, blocking) should be provided in a separate document that is unavailable to those who enrol participants or assign interventions	18
Allocation concealment mechanism	#16b	Mechanism of implementing the allocation sequence (eg, central telephone; sequentially numbered, opaque, sealed envelopes), describing any steps to conceal the sequence until interventions are assigned	18
Allocation: implementation	#16c	Who will generate the allocation sequence, who will enrol participants, and who will assign participants to interventions	18
Blinding (masking)	#17a	Who will be blinded after assignment to interventions (eg, trial participants, care providers, outcome assessors, data analysts), and how	19
Blinding (masking): emergency unblinding	#17b	If blinded, circumstances under which unblinding is permissible, and procedure for revealing a participant's allocated intervention during the trial	19
**Methods: Data collection, management, and analysis**			

For peer review only

Data collection plan	#18a	Plans for assessment and collection of outcome, baseline, and other trial data, including any related processes to promote data quality (eg, duplicate measurements, training of assessors) and a description of study instruments (eg, questionnaires, laboratory tests) along with their reliability and validity, if known. Reference to where data collection forms can be found, if not in the protocol	13
Data collection plan: retention	#18b	Plans to promote participant retention and complete follow-up, including list of any outcome data to be collected for participants who discontinue or deviate from intervention protocols	20
Data management	#19	Plans for data entry, coding, security, and storage, including any related processes to promote data quality (eg, double data entry; range checks for data values). Reference to where details of data management procedures can be found, if not in the protocol	21
Statistics: outcomes	#20a	Statistical methods for analysing primary and secondary outcomes. Reference to where other details of the statistical analysis plan can be found, if not in the protocol	21
Statistics: additional analyses	#20b	Methods for any additional analyses (eg, subgroup and adjusted analyses)	21
Statistics: analysis population and missing data	#20c	Definition of analysis population relating to protocol non-adherence (eg, as randomised analysis), and any statistical methods to handle missing data (eg, multiple imputation)	21
**Methods: Monitoring**			
Data monitoring: formal committee	#21a	Composition of data monitoring committee (DMC); summary of its role and reporting structure; statement of whether it is independent from the sponsor and competing interests; and reference to	21

For peer review only

		where further details about its charter can be found, if not in the protocol. Alternatively, an explanation of why a DMC is not needed	
Data monitoring: interim analysis	#21b	Description of any interim analyses and stopping guidelines, including who will have access to these interim results and make the final decision to terminate the trial	21
Harms	#22	Plans for collecting, assessing, reporting, and managing solicited and spontaneously reported adverse events and other unintended effects of trial interventions or trial conduct	22
Auditing	#23	Frequency and procedures for auditing trial conduct, if any, and whether the process will be independent from investigators and the sponsor	N/A - Small scale trial so data will not be audited
**Ethics and dissemination**			
Research ethics approval	#24	Plans for seeking research ethics committee / institutional review board (REC / IRB) approval	22
Protocol amendments	#25	Plans for communicating important protocol modifications (eg, changes to eligibility criteria, outcomes, analyses) to relevant parties (eg, investigators, REC / IRBs, trial participants, trial registries, journals, regulators)	22
Consent or assent	#26a	Who will obtain informed consent or assent from potential trial participants or authorised surrogates, and how (see Item 32)	19
Consent or assent: ancillary studies	#26b	Additional consent provisions for collection and use of participant data and biological specimens in ancillary studies, if applicable	33
Confidentiality	#27	How personal information about potential and enrolled participants will be collected, shared, and maintained in order to protect confidentiality before, during, and after the trial	20

For peer review only

Declaration of interests	#28	Financial and other competing interests for principal investigators for the overall trial and each study site	1
Data access	#29	Statement of who will have access to the final trial dataset, and disclosure of contractual agreements that limit such access for investigators	1
Ancillary and post trial care	#30	Provisions, if any, for ancillary and post-trial care, and for compensation to those who suffer harm from trial participation	20
Dissemination policy: trial results	#31a	Plans for investigators and sponsor to communicate trial results to participants, healthcare professionals, the public, and other relevant groups (eg, via publication, reporting in results databases, or other data sharing arrangements), including any publication restrictions	23
Dissemination policy: authorship	#31b	Authorship eligibility guidelines and any intended use of professional writers	23
Dissemination policy: reproducible research	#31c	Plans, if any, for granting public access to the full protocol, participant-level dataset, and statistical code	23
**Appendices**			
Informed consent materials	#32	Model consent form and other related documentation given to participants and authorised surrogates	33
Biological specimens	#33	Plans for collection, laboratory evaluation, and storage of biological specimens for genetic or molecular analysis in the current trial and for future use in ancillary studies, if applicable	N/A - None collected

Notes:

- 5c: N/A - Study Sponsor and Funders had no role
- 5d: N/A - No Data Monitoring Committee was used
- 23: N/A - Small scale trial so data will not be audited

For peer review only

- 33: N/A - None collected The SPIRIT Explanation and Elaboration paper is distributed under the terms of the Creative Commons Attribution License CC-BY-NC. This checklist was completed on 08. July 2021 using https://www.goodreports.org/, a tool made by the EQUATOR Network in collaboration with Penelope.ai

For peer review only

BMJ Open

Protocol for a randomized controlled trial investigating an intervention to boost decentering in response to distressing mental experiences during adolescence: The Decentering in Adolescence Study (DECADES)

Journal:	*BMJ Open*
Manuscript ID	bmjopen-2021-056864.R1
Article Type:	Protocol
Date Submitted by the Author:	21-Jan-2022
Complete List of Authors:	Bennett, Marc; MRC Cognition and Brain Sciences Unit<br>Knight, Rachel; MRC Cognition and Brain Sciences Unit,<br>Dunning, Darren; University of Cambridge,<br>Archer-Boyd, Alan; MRC Cognition and Brain Sciences Unit<br>Blakemore, Sarah-Jayne; University of Cambridge,<br>Dalmaijer, Edwin; MRC Cognition and Brain Sciences Unit<br>Ford, Tamsin; University of Cambridge, Psychiatry<br>Williams, J Mark; University of Oxford, Psychiatry<br>Clegg, Hannah; MRC Cognition and Brain Sciences Unit<br>Kuyken, Willem; University of Oxford, Psychiatry<br>So, Tierney; University of Cambridge<br>Wright, Gem; MRC Cognition and Brain Sciences Unit<br>Lenaert, Bert; Maastricht University, Faculty of Healh, Medicine and Life Sciences<br>Vainre, Maris; Cambridge University, MRC Cognition and Brain Sciences Unit<br>Watson, Peter; University of Cambridge, MRC Cognition and Brain Sciences Unit<br>Dalgleish, Tim; Medical Research Council Cognition and Brain Sciences Unit,
**Primary Subject Heading**:	Mental health
Secondary Subject Heading:	Evidence based practice
Keywords:	Child & adolescent psychiatry < PSYCHIATRY, Anxiety disorders < PSYCHIATRY, Depression & mood disorders < PSYCHIATRY, Clinical trials < THERAPEUTICS, PREVENTIVE MEDICINE

SCHOLARONE™
Manuscripts

Protocol for a randomized controlled trial investigating an intervention to boost decentering in response to distressing mental experiences during adolescence: The Decentering in Adolescence Study (DECADES)

Bennett, M.P.[1]*, Knight, R. [1]*, Dunning, D.[1], Archer-Boyd, A.[1], Blakemore, S-J., Dalmaijer, E.[1], Ford, T.[2], Williams, J. M. G.[3], Clegg, H.[1], Kuyken, W.[3], So, T.[1], Wright, G.[1], Lenaert, B.[4,5,6], Vainre, M. [1], Watson, P.,[1] MYRIAD team, & Dalgleish, T. [1,7]

1- Medical Research Council- Cognition and Brain Sciences Unit, University of Cambridge, UK.

2- Department of Psychiatry, University of Cambridge, UK.

3- Department of Psychiatry, University of Oxford, UK.

4- Faculty of Health, Medicine and Life Sciences, Maastricht University, Maastricht, NL.

5- Limburg Brain Injury Centre, Limburg, NL.

6- Faculty of Psychology, Open University, Heerlen, the Netherlands

7- Cambridgeshire and Peterborough NHS Foundation Trust

*co-first authors

Trial number: ISRCTN14329613

Version: 4.3

Correspondence for this article should be addressed to Miss Rachel Knight or Dr. Marc Bennett, Medical Research Council- Cognition and Brain Sciences Unit, 15 Chaucer

For peer review only

Road, Cambridge, CB2 7EF. E-mail: Rachel.knight@mrc-cbu.cam.ac.uk, marc.bennett@mrc-cbu.cam.ac.uk

Data Statement

The datasets generated during and/or analysed during the current study will be stored in a publically available repository. Following the publication of the first manuscript, a fully anonymized dataset will be made available online on open-access databases whose servers are located within the UK or Europe. Data processing scripts and programming codes will also be made available online.

Conflict of Interest Statement

The authors declare no competing interests.

Funding

This project is funded by a Wellcome Strategic Award (Wellcome Trust, ref 104908/Z/14/z; awarded to TD, SJB, FT, WK, MW). The contribution of MB was partially supported by a Wellcome Trust Active Ingredients in Mental Heatlh Commission. RK is funded by an Economic and Social Research Council Doctoral Fellowship (ref SUAI/067).

Keywords

Decentering, adolescence, psychological distress, intervention, ecological momentary assessment

For peer review only

The **MYRIAD** Team comprises Saz Ahmed, PhD, of University College London, Susan Ball, MSc, of University of Exeter, Nicola Dalrymple, MSc, of University of Oxford, Katie Fletcher, HSD, of University of Oxford, Lucy Foulkes, PhD, of University College London, Poushali Ganguli, MSc, of Kings College London, Cait Griffin, MSc, Kirsty Griffiths, MSc, of University of Cambridge, Konstantina Komninidou, BEd, of University of Oxford, Suzannah Laws, BSc, of University of Oxford, Jovita Leung, MSc, of University College London, Jenna Parker, MSc, of University of East Anglia, Blanca Piera Pi-Sunyer, MSc, of University College London, J. Ashok Sakhardande, BSc Hons, Jem Shackleford, MA, MSc, Kate Tudor, PhD, of University of Oxford, and Brian Wainman, BEng, of Plymouth University. These individuals have worked across the MYRIAD strategic award "Promoting Mental Health and Building Resilience in Adolescence: Investigating Mindfulness and Attentional Control"; they are acknowledged as group authors in this article for their substantial contributions to the project development, in accordance with the MYRIAD Dissemination Protocol

For peer review only

Abstract

Introduction. Decentering describes the ability to voluntarily adopt an objective self-perspective from which to notice internal, typically distressing, stressors (e.g. difficult thoughts, memories, and feelings). The reinforcement of this skill may be an active ingredient through which different psychological interventions accrue reductions in anxiety and/or depression. However, it is unclear if decentering can be selectively trained at a young age and if this might reduce psychological distress. The aim of the current trial is to address this research gap. **Methods and analysis.** Adolescents, recruited from schools in the UK and the EU (n = 57 per group, age range = 16-19 years), will be randomised to complete five-weeks of decentering training, or an active control group that will take part in a combination of light physical exercise and cognitive training. The co-primary training outcomes include a self-reported decentering inventory (i.e. the Experiences Questionnaire) and the momentary use of decentering in response to psychological stressors, using experience sampling. The secondary mental health outcomes will include self-reported inventories of depression and anxiety symptoms, as well as psychological wellbeing. Initial statistical analysis will use between-group analysis of covariance to estimate the effect of training condition on self-rated inventories, adjusted for baseline scores. Additionally, experience sampling data will be examined using hierarchical linear models. **Ethics and dissemination.** This study was approved by the Cambridge Psychology Research Ethics Committee, University of Cambridge (PRE.2019.109). Findings will be disseminated through typical academic routes including poster/paper presentations at (inter)-national conferences, academic institutes and through publication in peer-reviewed journals.

Trial registration number: ISRCTN14329613

For peer review only - http://bmjopen.bmj.com/site/about/guidelines.xhtml

Article Summary

Strengths and limitations of this study

Strengths.

- We aim to strengthen an active and pan-therapeutic skill that features across different psychological interventions, and may improve well-being in adolescents at high risk of mental health difficulty.

- The intervention will be highly accessible by capitalising on extant social media and streaming services.

- Ecological momentary assessment will facilitate a temporally sensitive investigation into the effects of decentering training in everyday life.

Limitations.

- A lack of face-to-face interaction between experimenters and participants may undermine adherence.

- The study may be demanding as it requires daily, albeit brief, engagement over a several weeks.

For peer review only

Introduction

Background

Anxiety and depression are a considerable public health challenge. Since these difficulties often begin before the age of 24, responding to this challenge requires a special focus on adolescence[1]. It has been suggested that an effective way to promote early mental health is through universal approaches[2-4]; these are interventions that can be offered across a broad community of young people, irrespective of symptom severity. The goal of such universal approaches is to not only reduce distress in young people experiencing symptoms, but also to prevent future anxiety and depression onset in those who may be asymptomatic. However, their effectiveness depends on how well an intervention focuses on psychological experiences and mechanisms that are relevant across the entire spectrum of mental health, from risk to resilience, and through to flourishing. One strategy is therefore to target widely relevant therapeutic skills that can help manage distressing inner experiences (e.g. unpleasant feelings, thoughts, memories), which can otherwise trigger short-term distress and long-term risk of anxiety and depression onset. Psychological decentering, or decentering for short, may be one such skill.

Psychological decentering is a key concept within psychological therapy and science[5]. It is characterised as an adaptive self-observation style wherein one can attend to, not just the mental content of distressing inner experiences, but also its underlying cognitive nature[6,7]. Thus, decentering relates to how a person interacts with the difficult inner events prompted by experiences in daily life – it involves the ability to generate an objective self-perspective wherein one can notice mental experiences as imperfect models of the real-world rather than precise reflections. For example, a person might notice “I am thinking I am depressed right now” instead of only noticing

For peer review only

and then believing the thought "I am depressed"[8]. Decentering resultantly restricts the disproportionate influence psychological stressors may have on affect, behaviour and sense-of-self. Indeed, brief therapeutic exercises that stimulate a decentred self-perspective have been shown to reduce negative emotional reactivity towards stressors like unpleasant memories and negative self-relevant statements[9-13]. Evidence also indicates self-reported decentering traits in adolescence are associated with fewer overall symptoms of anxiety and depression[14-17].

Decentering is a malleable skill that can be refined through psychological intervention[7]. Medium to large changes in self-reported decentering are reported in adults experiencing anxiety and depression following a range of psychological interventions. This includes cognitive behavioural therapy (CBT)[18,19], acceptance based approaches[20-22] and mindfulness training[23-25]. In fact, emerging evidence suggests that intervention-related increases in decentering are a common pathway through which different psychological interventions deliver reductions in anxiety and depression[21,26,27]. This has encouraged some investigations into whether or not decentering can be trained selectively so to improve mental health. For example, Travers-Hill et al. trained adults with a diagnosis of recurrent depression currently in remission but experiencing residual depression symptoms to practice self-distancing techniques in response to difficult autobiographical memories[28]. This resulted in a significant increase in self-reported measures of decentering relative to an active comparison group who learned strategies to manage maladaptive avoidance tendencies. Additionally, those who learned to apply this decentering-related technique reported less distress towards negative memories at post-intervention even in the absence of instructions to self-distance.

For peer review only

Therapeutic exercises that target decentering skills are already nested within different psychological interventions[7]. Emerging evidence also indicates that these components can be delivered in isolation so to selectively boost decentering and reduce risk of relapse in formerly depressed adults[28]. However, a remaining question is 'can decentering skills be selectively reinforced during adolescents, and if so, through what therapeutic techniques?' This is the primary research question guiding the current study. It is also unclear if boosting adolescent decentering skills might impact on emotion and mental health. The current study will therefore evaluate, as a secondary question, how decentering training impacts anxiety, depression and emotional reactivity in adolescents. This information will inform future research trials and ultimately help in the investigation of effective universal approaches in youth mental health. A straightforward way to address these questions might be to examine the impact of decentering training on trait-level measure of decentering and symptom severity at critical time points, e.g. pre and post-intervention design. However, this approach lacks the sensitivity required to detect subtle changes in decentering in response to momentary psychological stressors and their impact on emotion. A design that also includes ecological momentary assessment or experience sampling methods (ESM) could therefore facilitate a more ecological investigation of the use of decentering in everyday life as well as its impact on transient psychological stressors and emotional states.

Current study objectives

The primary aim of the current study is to investigate if decentering can be improved during adolescence via an intensive decentering training programme. This will be investigated (1a) at a trait level by examining changes in self-reported experience of decentering (via a standard decentering inventory) and (1b) at a

For peer review only

situational level by examining self-reported use of decentering skills in response to momentary psychological stressors (via ESM assessments). Here, repeated measures of participants' negative emotional reactivity towards momentary inner experiences (e.g. feelings, thoughts and memories) will be recorded over a five day baseline period and across the duration of the intervention. A secondary aim is to explore potential mental health outcomes associated with reinforced decentering skills. This will be investigated (2a) at the symptom level by examining relative change in self-rated anxiety, depression and psychological well-being and (2b) at the situational level by recording emotional reactivity towards momentary psychological stressors using ESM. A final exploratory aim is to investigate some of the cognitive correlates of decentering and training. Thus, this study will answer the following research questions: (1a) Is decentering training during adolescence associated with increased decentering reports relative to an active control condition? (1b) Is decentering training during adolescence associated with increased decentering in response to negative mental events relative to an active control condition? (2a) What is the impact of decentering training on youth mental health relative to an active control condition? (2b) What is the impact of decentering training on emotional reactivity towards momentary negative mental events relative to an active control? (3) What are the cognitive correlates of decentering training during adolescence?

This study will address these research questions in a cohort of adolescents who are at increased risk of depression. This is a first step towards our long-term goal to develop a universal intervention that targets psychological experiences and mechanisms that are broadly relevant in young people. Adolescents experiencing elevated symptoms of depression will be recruited from both the UK (e.g. sixth form colleges) and EU (e.g. secondary level education high schools) and will be randomly

For peer review only

assigned to a five week decentering training programme or an active control. Both programmes will be delivered remotely via extant social media platforms; this means they can be delivered at low-cost, are easily accessible and are flexible regarding other commitments of our participants. The decentering intervention comprises 10-15 minute of audio exercises to be made available each weekday through music/podcast streaming services, e.g. *Spotify*, *iTunes*, *Deezer*, *Podcast Addict.* Participants will practice a specific decentering technique each week, which has been adapted from either the literature or an extant psychological therapy. Participants will also practice brief mindfulness-based grounding exercises each week to encourage the monitoring of inner experiences like emotional thoughts, memories and feelings. The overall goal of this programme is therefore to teach adolescents different ways to generate an objective self-perspective from which they can interact with negative psychological events encountered in daily life. Rather than rehearsing decentering techniques, the active control condition comprises 10-15 minutes of gamified cognitive tasks for completion on a personal smartphone. In addition, participants in this condition will be given short physical movement routines to complete in lieu of mindfulness-based grounding.

Methods

Study design

The design is a randomised controlled feasibility trial (comparing a psychological decentering training to an active control programme) with school aged adolescents (aged 16-19 years). The study will run from December 2021 until July 2022, split into several cohorts. Self-rated inventories of training and mental health outcomes will be assessed at three time points: within one week prior to the start of

For peer review only

programme (baseline); within the third week of training (mid-intervention); and one week after the programme has finished (post-intervention).

Participants and inclusion/exclusion criteria

Two groups of older adolescents (n = 57 per group; age range = 16–19 years) will be recruited from the UK and Ireland. Eligible participants must consent to completing (1) a five day ESM baseline assessment, (2) five weeks of the assigned training programme and (3) assessment measures at baseline, mid-intervention and post-intervention. In exchange for their time, participants will be compensated with shopping vouchers worth (1) £50 (for completing ESM measures), (2) £100 pounds (for completing five weeks of training) and (3) £50 (as a bonus for completing all assessments). Eligible participants must also report a score of 16 or above on the Centre for Epidemiological Studies-Depression Scale (CES-D), based on standard CES-D cut-offs. Participants must also have access to a laptop/desktop computer and a personal smartphone device.

Participants will be excluded if they: (1) currently take part in a regular (once or more per week) yoga and/or mindfulness class/workshop, (2) have participated in prior formal meditation training or a mindfulness-based stress reduction course (3) are currently experiencing chronic illness (e.g. epilepsy, chronic pain, cancer), (4) lack fluency in English (5) have a recent diagnosis of, and are currently receiving medical/psychological treatment for, a mental health condition including (but not limited to) anxiety disorder, major depressive disorder or a traumatic stress disorder and (6) have a diagnosis of a neurodevelopmental condition such as Autism Spectrum Disorder or Attention Deficit/Hyperactivity Disorder. Both the decentering programme and active control programme require adolescents to be without severe hearing difficulties since both conditions require listening to audiotapes. As the control

For peer review only

condition involves physical movement routines it may not be appropriate for specific individuals, such as individuals with limited mobility. Any volunteer who meets the exclusion criteria will be accommodated as best as possible (e.g. assignment to the most appropriate condition in the case of a physical disability) and their data will not be included in the analysis.

Recruitment

Participants will be recruited from the general community through a combination of existing collaborations with schools in the UK and Ireland. We will also recruit UK and Irish volunteers via online research platforms (e.g. Prolific; www.prolific.co) as well as extant panels of adolescent research volunteers via the MRC-Cognition and Brain Sciences Unit (University of Cambridge). Finally, and if necessary, we will also conduct a targeted recruitment campaign to recruit young people and schools using posters, pamphlets and online adverts on social media.

Sample size

The primary training outcome is self-rated decentering and this will be assessed using both a popular inventory of decentering (Experiences Questionnaire; EQ[8]) and ESM items. There is no consensus on how to best conduct a statistical power analysis for ESM data. Power and sample calculations were therefore based on the effect of training condition on EQ scores. A power analysis calculated in G*Power indicated that a total sample size of 90 is powered at 80% to observe a significant effect of training condition (decentering vs. control) on post-intervention decentering with a medium effect size of $f = 0.3$ (alpha = .05) after adjusting for baseline EQ-score. A medium effect size is plausible given that previous research reports a medium–large effect of extant psychological intervention on EQ scores. An attrition rate of around 20% is anticipated based on our previous research. Therefore, with 57 participants in

For peer review only - http://bmjopen.bmj.com/site/about/guidelines.xhtml

each group, the current study is adequately powered to observe a medium to large effect of decentering training on our candidate training outcome measures and to determine the nature of this interaction. Regarding our secondary mental health outcomes (i.e. anxiety, depression and psychological wellbeing), a sample size of 57 per group is consistent with our previous research to evaluate the impact of novel psychological interventions. That is, such group sizes can provide a reasonable range of point estimates of the effect on mental health outcome measures that are sufficient to guide later research; for example, fifty-seven participants per group is powered at 88% to observe a significant ($p < .05$) main effect of group (decentering versus active control) on baseline adjusted mental health outcomes with an effect size $f = 0.26$.

Intervention

Decentering Training.

A five week psychological decentering training programme was developed by MB, RK and TD, based on our previous protocol (Travers-Hill et al., 2017). This involves audio-recorded scripts and an accompanying work book that guides participants through four types of decentering technique; this structure is partly based on a recent taxonomy of self-distancing (a construct closely related to decentering)[29]. The decentering techniques include: (Week 1) *spatial distancing* wherein individuals are taught to re-imagine negative memories from a physically distant perspective (e.g. 'replay the memory but as if you're a fly on the wall'); (Week 2) *verbal distancing/cognitive defusion* wherein individuals are taught to re-phrase negative self-relevant statements in a way that challenges its literal value and influence over affective behaviour (e.g. replacing first person pronouns with one's name); (Week 3) *temporal distancing* wherein individuals are taught to re-consider specific worries from a temporally distant future (e.g. 'how would this seem in 5 years?'); and (Week 4)

For peer review only - http://bmjopen.bmj.com/site/about/guidelines.xhtml

objective distancing wherein individuals are taught to adopt a third-person perspective towards negative memories (e.g. 'what is the effect of reliving a difficult memory from the perspective of an objective observer?). Week 5 is a revision week, during which participants will be encouraged to practice a different technique from weeks 1 to 4 each day. These techniques were selected since they are directly targeted at how adolescents relate to and observe day-to-day psychological stressors. Specifically, the goal is to teach adolescents concrete ways to generate an objective (or distanced) self-perspective in response to everyday feelings, thoughts and memories that are unpleasant. We assume this training will develop participants' decentering ability above their baseline levels.

Each week will involve 5 audio-recorded exercises (10-15 minutes) that will be made available using common streaming services. One exercise will be posted each weekday, Monday to Friday. The first two exercises (Monday and Tuesday) are brief mindfulness grounding exercises designed to promote open monitoring of psychological experiences. The next three exercises (Wednesday to Friday) are decentering training exercises as described above. Adherence to the programme will be encouraged by directly contacting the participants in week 1 and week 3 to discuss their experiences so far and allow troubleshooting. Participant engagement will be monitored by reviewing the number of completed workbook exercise at the end of the five weeks. Participants will also complete daily diaries. These will include five questions about the intervention and its application, such as "Did you complete any of the programme exercises today?" or "During the day, were you able to apply the skills you've learned from the exercises?" (see Appendix B). A participant's engagement in the trial will be discontinued if they elect to withdraw their participation or if they

For peer review only

experience serious physical/mental health difficulties that necessitate medical or psychological intervention.

Physical and Cognitive Exercise.

A five week active control programme was developed (MB, RK, and TD). This contains two elements that roughly match the decentering training for time and cognitive engagement. First, guided physical movement routines will be completed in lieu of mindfulness grounding exercises (Monday-Tuesday). These movements are intended to emulate the physiological nature of grounding exercises but without an internal focus. Participants will watch short videos in which one member of the research team (RK) illustrates a basic series of body stretches. Accompanying audio provides additional direction and this was recorded using the same voices from the decentering training programme. Each video comprises 15 stretches, with each stretch held for 30 seconds and a 10 second break between stretches. The physical movements were selected for their ease and accessibility. Care was also taken to select movements that are safe and cater to a range of physical abilities.

Second, gamified versions of standard cognitive tasks will be completed in lieu of the decentering training (Wednesday-Friday). These games are intended to emulate the cognitive effort associated with decentering exercises. Each participant will have a link that allows them to select one of three games, which can be completed on a personal smart phone (for game outlines, see https://osf.io/aw6c5). Games include: (1) a Multi-Target Visual Search Task wherein participants search and respond to specific targets within a broader stimulus array; (2) a Go/NoGo Task wherein a speeded response is made in response to a 'go' signal but inhibited in response to a 'no go' signal: and (3) a Digit Recall Task wherein participants must recall a sequence on a number pad (i.e. a digit-span task). Adherence to the programme will be

For peer review only

encouraged by contacting the participants in week 1 and week 3 to discuss their experiences. Engagement will be monitored by reviewing task completion rates and performance measures (response times and accuracy) at the end of the five weeks.

Outcomes

There are two types of outcome measures in this study (see Figure 1 for Timeline). These include measures of (1) decentering and (2) mental health. We will also examine if there are any far-transfer effects of decentering training by including measures of (3) cognitive performance. Figure 1 provides a schematic overview of the assessment timeline.

Decentering outcome measures.

The primary training outcome measure is self-rated decentering as measured using the experiences questionnaire (EQ). A secondary decentering outcome measure is (2) the self-rated use of psychological decentering in response to psychological stressors as measured using ESM. These measures have been included to address research questions 1a and 1b, respectively.

Experiences questionnaire. This widely-used self-report assessment features 11 items that explore an individual's tendency to psychologically decenter from difficult subjective experiences in day-to-day life (e.g. 'I can observe unpleasant feelings without being drawn into them')[8]. Items are answered using a 5-point Likert scale from 1 = 'Never' to 5 = 'All the time'). Psychometric properties were found to be satisfactory (Cronbach's α=.893; convergent validity r>.46)[33]. Scores on the EQ are positively associated with other decentering-related constructs like experiential avoidance (a tendency to attempt to evade difficult thoughts and feelings)[30-34] and cognitive reappraisal (an ability to reconceptualise situations to modify emotional impact)[27,35]. Finally, EQ scores improve over the course of behavioural and cognitive therapies; the

For peer review only - http://bmjopen.bmj.com/site/about/guidelines.xhtml

magnitude of such change is associated with key outcomes like symptom severity and quality-of-life[23,26,27,36,37]. The EQ will be administered at baseline, mid-intervention and post-intervention. We expect mean EQ scores will increase in the decentering training group relative to the active control condition.

ESM decentering items. All ESM items are described in Table 1 (see overleaf). Some items relate primarily to the use of decentering skills. Other ESM items relate to secondary mental health outcomes and are described in the next section. However, all ESM items will be administered during each sample point. Two ESM items have been developed to estimate the momentary use of decentering in response to psychological stressors like difficult feelings, memories or thoughts. These are "*Since the last beep, I was able to distance myself from unpleasant feelings*" and "*Since the last beep, I was able to distance myself from unpleasant things on my mind*". Items are answered using a 7-point Likert scale ranging from 1 = 'Not at all' to 7 = 'Very much'). ESM items will be delivered four times daily across a five day ESM baseline period and the five weeks of training (see Figure 1). All ESM items are described in Table 1 (see overleaf). We expect momentary reports of decentering to increase over time in the decentering training group relative to the active control condition.

For peer review only

Table 1. Experience Sampling items

Item	Question	Rating	Purpose	Conditional
1	Right now, I feel…	-5 = Very Negative, 5 = Very Positive	Affect	Always Shown
2	I Feel:		Feelings/Mood	Always Shown
i	*Happy*	1 = Not at all, 7 = Very Much	Feelings/Mood	Always Shown
ii	*Relaxed*	1 = Not at all, 7 = Very Much	Feelings/Mood	Always Shown
iii	*Satisfied*	1 = Not at all, 7 = Very Much	Feelings/Mood	Always Shown
iv	*Enthusiastic*	1 = Not at all, 7 = Very Much	Feelings/Mood	Always Shown
v	*Nervous*	1 = Not at all, 7 = Very Much	Feelings/Mood	Always Shown
vi	*Sad*	1 = Not at all, 7 = Very Much	Feelings/Mood	Always Shown
vii	*Irritated*	1 = Not at all, 7 = Very Much	Feelings/Mood	Always Shown
viii	*Stressed*	1 = Not at all, 7 = Very Much	Feelings/Mood	Always Shown
3	Since the last beep, I felt sucked in by negative feelings?	1 = Not at all, 7 = Very Much	ER | Immersion negative feelings	Always Shown
4	Since the last beep, I was able to distance myself from negative feelings?	1 = Not at all, 7 = Very Much	ER | Distance negative feelings	Always Shown
5	Since the last beep, I tried to distract myself from negative feelings?	1 = Not at all, 7 = Very Much	ER | Distraction	Always Shown
6	Since the last beep, I noticed an unpleasant thought or memory?	1 = Not at all, 7 = Very Much	Thoughts/memories onset	Always Shown
7	Since the last beep, I was upset by an unpleasant thought or memory?	1 = Not at all, 7 = Very Much	Thoughts/memories onset	Always Shown
8	The unpleasant thing on my mind was about …	Myself, Others, A combination of these, None of these	Content- self/other	Contingent | (if Q6 != 1)
9	The unpleasant thing on my mind was about …	Social things, Professional things, A combination of these, None of these	Content- social/non-social	Contingent | (if Q6 != 1)
10	The unpleasant thing on my mind was about …	The past, The present, The future, A combination, None of these	Content- past/future	Contingent | (if Q6 != 1)
12	Since the last beep, I felt sucked in by unpleasant things on my mind.	1 = Not at all, 7 = Very Much	ER | Immersion in thoughts/memories	Always Shown
13	Since the last beep, I able to distance myself from unpleasant things on my mind.	1 = Not at all, 7 = Very Much	ER | Distance from thoughts/memories	Always Shown
14	Since the last beep, I tried to think differently about things so to feel better.	1 = Not at all, 7 = Very Much	ER | Re-appraisal	Always Shown
15	Since the last beep, I tried to distract myself from unpleasant things in my mind.	1 = Not at all, 7 = Very Much	ER | Distract	Always Shown
16	Since the last beep, I feel I benefited from the daily programme.	1 = Not at all, 7 = Very Much	Usefulness	Always Shown
Branch 1	Right now, I am …	Indoors, Outdoors	Branch	Contingent | (if Q6 == 1)
Branch 2	Right now, I am …	Alone, With others	Branch	Contingent | (if Q6 == 1)
Branch 3	Since the last beep, I felt social.	1 = Not at all, 7 = Very Much	Branch	Contingent | (if Q6 == 1)

Secondary mental health outcomes.

Our secondary mental health outcomes are (1) self-rated anxiety, depression and psychological well-being and (2) self-rated emotional reactivity following a negative mental experience (e.g. unpleasant thought, feeling or memory) as measured using ESM. These measures have been included to address research questions 2a and 2b.

Mental health symptoms. This study will explore the potential impact of decentering training on mental health outcomes at baseline, mid-intervention and post-intervention. Previous findings suggest that decentering is negatively associated with anxiety and depression [14-16] as well as feelings of anger in adolescents[38,39]. We will therefore include measures of each of these difficulties. Anxiety symptoms will be assessed using the Revised Child Anxiety and Depression Scale-Short Version (RCADS-15)[40]. The RCADS-SV is a 15 item scale measuring the reported frequency of various symptoms of anxiety and low mood. Internal consistency has been reported as good (Cronbach's α = .7-.96), as were test-retest coefficients and convergent validity. Depression symptoms will be assessed using the Centre for Epidemiology Depression Scale (CES-D)[41]*.* The CES-D is a 20 item inventory measuring depressive symptoms experienced in the past week. Internal consistency is good, with Cronbach's α = .85-.90. Concurrent and construct validity have been demonstrated. Anger will be measured using the State-Trait Anger Expression Inventory-2 Child and Adolescent (STAXI-2)[42], which is a 35 item self-report measuring anger expression and control in adolescents. The STAXI exhibits good reliability, with Cronbach's alpha coefficients ranging from .81 to .93, with good convergent validity.

Two measures will also be included to estimate the impact of decentering training on psychosocial strengths/difficulties and general wellbeing. The Strengths

For peer review only

and Difficulties Questionnaire (SDQ) is a screening tool with 5 subscales (Emotional Symptoms, Conduct Problems, Hyperactivity/Inattention, Peer Relationships, and Prosocial Behaviour) as well as an impact supplement to estimate psycho-social functioning. This tool was included to capture behavioural symptoms such as conduct difficulties and hyperactivity. The SDQ exhibits strong internal consistency, moderate test-retest reliability and good concurrent validity. Psychological wellbeing will be measured using the Warwick-Edinburgh Mental Wellbeing Scale (WEMWBS)[49]. The WEMWBS is a 14 item measure of positive mental health including positive affect, functioning and inter-personal relationships. The WEMWBS shows high internal consistency, and good convergent and construct validity. Measures will be administered at baseline, mid-intervention and post-intervention.

ESM emotional reactivity. This study will examine if the lagged effect of the decentering from difficult thoughts, feelings or memories (time = n) on later affect (time = $n + 1$) changes across training. One ESM items will capture general affect: 'Right now I feel…', answered on a 11 point Likert scale ranging from -5 = Very negative to +5 =Very positive. Two separate ESM items will capture the self-initiated use of decentering skills in response to negative mental experiences (Table **1**. These are: 'Since the last message, I was able to distance myself from [a] a negative feeling *or* [b] unpleasant things on my mind'. Additional ESM items will check for the occurrence of negative mental experiences. These include checks of (a) negative feelings by asking '*I feel Nervous/Sad/Irritated/Stressed*' and (b) unpleasant cognitive events by asking '*Since the last beep, I was upset by a thought or memory*'. This will be scored on a 7 point Likert scale from 1 = Not at all to 7 = Very Much.

One item will assess the occurrence of unpleasant thoughts: 'since the last bee, I noticed an unpleasant thought or memory'. This will allow us to control for the

For peer review only

presence of unpleasant cognitive events from which participants can decenter. If the participant's response is anything other than 'not at all', they will then complete three items to the content of these thought (Table 1, items 8-10). If the participant's response is 'not at all', they will complete three unrelated 'branching' items (Table 1, branch items 1-3). This means the same number of item will always be administered.

Additional self-rated assessments. Participants will complete several additional self-rated assessments for the purpose of exploratory analysis. Participants will also complete a brief end of day questionnaire that will include a measure of mood, assessing presence and duration of different mood states. Nine moods will be investigated– happy, lively, content, satisfied, depressed, bored, anxious, irritable and tense. These items will allow for exploratory analysis of how individuals' moods may relate to the two experimental conditions. The end of day questionnaire will also include items investigating whether participants have completed any exercises from their programme (decentering or control), how difficult these exercises were to complete, and whether these skills were relevant to their everyday life. This section is included both as a measure of adherence to the programme, and as a broad measure of participant feedback.

Our team is currently developing an adolescent friendly self-rated decentering inventory. A provisional version of this scale will be included at pre- and post-intervention as part of its on-going validation. We are also investigating the relationship between decentering and other emotion regulation skills. We will therefore include: the Cognitive Emotion Regulation Questionnaire (CERQ)[43], which is a 36 item questionnaire cataloguing a range of emotion regulation strategies (Cronbach's α = .62-.85); the Difficulties in Emotion Regulation Scale (DERS)[44], which is a 36-item questionnaire investigating problems with emotion regulation (Cronbach's α = .94),

For peer review only

including 6 subscales: non-acceptance of emotional responses; difficulty in engaged in goal-directed behaviour; impulse control difficulties; lack of emotional awareness; limited access to emotion regulation strategies; and lack of emotional clarity: and the Child and Adolescent Mindfulness Measure (CAMM)[50], which is a 10 item measure exploring trait mindfulness in children and adolescents (Cronbach's α = .88). Participants will complete the Coronavirus Anxiety Scale[45] scales at baseline and post-intervention. This may help us address any unexpected impact of the COVID-19 pandemic on the intervention. To control for impact of patient expectation on intervention efficacy, a single item patient expectation scale will also be administered at baseline.

Far transfer effects.

This study will include performance-based tasks to explore potential far-transfer effects of decentering training on cognition (research question 3). These include (1) affective cognitive control and (2) self-perspective processing, both of which have been posited as theoretical components of decentering[6 29].These measures will be administered at baseline and post-intervention.

Affective cognitive control. This study will explore if decentering training influences the ability to maintain cognitive performance within emotional contexts (i.e. affective cognitive control). This can be estimated via the Emotional Stop Signal Task (ESST; Figure S1)[46]. On each trial, a neutral or negative valence image is presented before a 'go-signal' that requires a speeded button-press (75% of trials). Participants must quickly make the appropriate button-press unless the 'go-signal' is followed by a 'stop-signal (25%). Participants must withhold their response on these trials. The period between the go and stop-signal (stop signal delay; SSD) varies over the task. Following a successful stop trial, the task is made more difficult by increasing the SSD

For peer review only - http://bmjopen.bmj.com/site/about/guidelines.xhtml

by 25 ms. Following an unsuccessful stop trial, the task is made easier by decreasing the SSD by 25 m. This tracking algorithm typically causes a failure to stop on ~50% of trials. Sustained attention in emotional and non-emotional contexts will be estimated by calculating (1) the reaction time (RT) mean on go-trials and (2) the inter-trial variability coefficient (i.e. the RT mean divided by the RT standard deviation). Inhibitory control in emotional and non-emotional contexts will estimated by calculating the *stop signal reaction time (i*.e. mean RT – mean SSD).

Self-referential processing. This study will explore if decentering training influences the ability to flexibly shift one's self-perspective. This can be indexed using the own-body transformation task (OBT; Figure S2)[47]. On each trial, a schematic human figure is presented that face either with their front to the participant or their back to the participant. This figure also holds a black glove in one hand (e.g. left). Participants are instructed to imagine themselves in the body position of the figure and then judge whether the glove is held by the figures left of right hand. Performance is compared to a within-subject control task wherein participants judge the position of the glove without altering their self-representation. The outcome variables are mean RT and the percentage error rate. These are found to increase as a function of the self-perspective manipulation; that is, they are higher for front-facing characters.

Procedure

Data quality.

Checks of data quality will be included ad different stages of data collection and analysis. First, 'catch questions' will be embedded in self-report assessments to assess whether participants actively read questions (e.g. '[describe one]'). Second, participants self-rate the quality of their data at the end of each data collection period, e.g. []. Piloting revealed this to be a useful strategy. Third, all the standard markers of

For peer review only

appropriate behavioural performance will be checked when analysing task data (e.g. reaction times >250 ms and <3000 ms; chance-level accuracy).

Assignment of interventions.

Allocation. Following baseline assessment, participants will be stratified according to sex and depression severity (using the CES-D). They will then be randomly assigned to either the decentering training programme or the active control condition. This will be managed by the trial statistician (PW) using a minimization procedure. Participant allocation will be then shared with the research coordinator responsible for providing appropriate access links for the online intervention (MB and RK).

Blinding. Research coordinators (MB and RK) will be blinded during assessment, data pre-processing and statistical analysis. Due to the nature of the study, it is not possible to remain blinded during participant allocation. This is because each participant needs to be given a secure link to the online training programme, either decentering training or the active control. All outcome assessments will be completed online; participants will access the same link to complete online assessments, regardless of group. Thus, there are no in-person data collectors with an awareness of participants' allocation. Initial data will be pre-processed (e.g. calculation of summary scores like means, percentage accuracy, sums etc.). Pre-processing will be completed using pre-existing scripts coded in R and MATLAB (MB). At this point, the dataset will be shared with the trial statistician (PW) who will conduct the statistical analysis and who will be blind to participant allocation. Once the analysis is complete, the trial coordinators will un-blind the dataset by indicating which participants were allocated to which group.

Consent and Data Collection.

For peer review only

Recruited volunteers will complete an eligibility check during an initial phone call. Volunteers who are eligible will be invited to participate in the study. Written informed consent will be sought after an initial briefing that explains the data collection protocol. This briefing will also include a tutorial on downloading and using the experience sampling app (PsyMate2; www.psymate.eu). Participants will also have the opportunity to ask any questions. To discourage possible contamination, participants will be asked to keep the content of the study confidential from others. Once consent is provided, participants will be given an anonymous trial identification code and added to an encrypted list for allocation. Participants will also receive a URL link to complete baseline measures. Figure 1 illustrates the full schedule for outcome measures. The two-week ESM baseline assessment will also begin. At the end of this period, participants will receive a tutorial on accessing the intervention materials.

Over five weeks, participants in both interventions will access and rehearse exercises (Monday-Friday). Decentering training exercises are based on audio files to be delivered via their preferred music/post-streaming services (e.g. Spotify/Podcast Addict). The active control exercises are based on YouTube videos posted on a Private Channel. Each day, the PsyMate2 App will prompt participants to complete their daily exercise as well as complete any required ESM items. These will be sent four times per day. A brief survey will also be sent through the PsyMate2 App each evening to ask whether participants completed their daily exercises, and if so, to ask about their experiences as well as questions on their mood states (Supplemental Materials). At the end of Week 5, participants will be asked to complete the online assessment battery. Participants will then complete a debriefing session. During the debriefing session they will be given the opportunity to provide feedback on their experience of the study, and to ask any questions they may have. They will also be

For peer review only

given a list of relevant mental health resources should they feel the need to access them.

Participant Retention & Follow Up. Retention will be promoted in a number of ways. Firstly, participants will not be paid until receipt of their completed post-intervention assessment battery. Secondly, participants will be made aware that they can receive a bonus payment of £50 if they complete all of their assessments and daily diaries. Thirdly, telephone contact will be made with participants during week 1 and 3 to allow any troubleshooting and ensure that participants are filling in diaries and experience sampling appropriately. Telephone contact will also be made with a participant should they fail to complete mid-intervention assessments (Week 3).

Data Management and Statistical Analysis

Data Confidentiality and Management.

All participants will be given an anonymous trial identification code to use when submitting their completed assessments. A file linking the participant's name to their identification code will be saved in an encrypted and password protected file and stored on a secure, protected server within the MRC-Cognition and Brain Sciences Unit. This file will be deleted once the final round of data collection is complete. Outcome data will be gathered remotely using online platforms (e.g. Qualtrics; www.qualtrics.com) and an ESM data collection app. During periods of data collection, these data will be transferred to in-house secure servers within the MRC-Cognition and Brain Sciences Unit, University of Cambridge. The source data will be then deleted. Access to these data will be restricted to primary research team members (MB, RK, TD). Data management will be overseen by these team members. There will be no formal Data Management Committee (DMC) because it is a small scale trial.

Statistical Analysis.

For peer review only

Self-rated and performance-based measures. The statistical analysis will be conducted by the trial statistician (PW) who will be blind to training condition. The analysis will be conducted on an intention-to-treat basis and a similar initial analysis is planned for (i) the primary training outcome (i.e. self-rated EQ scores) and (ii) secondary mental health outcomes (i.e. CES-D, RCADS, STAXI-2, SDQ and WEMWBS). Analysis of covariance (ANCOVA) will be calculated to estimate the effect of training condition on outcome measures after adjusting for baseline performance and grouping stratification variables. This model will be calculated for our primary end-point, i.e. post-intervention (i.e. post-intervention outcome adjusted for baseline score). This model will additionally be calculated for our mid-intervention time point (i.e. mid-intervention outcome adjusted for baseline score) and post-intervention. There are no planned interim-analysis. If a subset of items of a measure ($\leq$ 20% within-measure missing data), a total score will be calculated using the mean score across the non-missing items; an approach we have reported elsewhere (www.osf.io/d6y9q). Otherwise, the score for that measure will be noted as missing. We will assume that data will be missing at random and address this using standard imputation approaches in R (R Core Team, 2013).

ESM analysis. Only participants who completed at least 33% of the ESM questionnaires will be included in the analyses, in line with ESM guidelines[48]. Statistical analyses will be performed using hierarchical linear models. These models are especially suited to deal with dependency inherent in the data due their hierarchical structure: ESM observations (level 1) nested within individuals (level 2). Potential confounders will be included in the model as covariates. First, a linear growth model will be estimated to assess the effect of condition (decentering training vs. active control) on decentering skills over time (i.e., the duration of the intervention). Second, time-lagged analyses will be performed to evaluate if higher self-rated decentering skills when confronted with a momentary psychological stressor (time = n) predict significantly lower negative affect ratings at a later time point (time = n + 1). We will also control for momentary negative affect ratings (at time = n). Random intercepts and random slopes models will be estimated. In hierarchical linear models, the fixed effects reflect the overall association between a predictor and the outcome of interest, whereas the random effects reflect individual differences in this association.

Ethics and Dissemination

Ethical Approval and Protocol Amendments.

This study was approved by the Cambridge Psychology Research Ethics Committee, University of Cambridge (PRE.2019.109). Approval for any protocol amendments will be sought by this committee. This includes methodological and/or trial management amendments. The online registration of the trial will also be updated in this situation and any amendments will be outlined in the trial manuscripts. This committee will also be contacted should we encounter any unexpected ethical concerns.

Dissemination.

For peer review only

The findings of this study will be disseminated through typical academic routes including poster/paper presentations at (inter)-national conferences, academic institutes and through publication in peer-reviewed journals. Given that some research questions are less exploratory than others, two key manuscripts are planned for the trial data. The first will describe the impact of training on self-rated decentering and mental health outcomes. The second will describe the exploratory research around the impact on cognitive performance measures. Findings will also be disseminated to the broader public through seminars and workshops with relevant stakeholders as well as online blogs and podcasts. These manuscripts will be completed and submitted for review irrespective of the nature of the observed effects. Following the publication of the first manuscript, a fully anonymized dataset will be made available online on open-access databases whose servers are located within the UK or Europe. Data processing scripts and programming codes will also be made available online.

Patient and Public Involvement

A youth advisory panel was formed from May to December 2020. This panel advised on the accessibility of self-rated measures of decentering. They also provided qualitative feedback on decentering as an emotional regulation stratagem and the potential for decentering training. This took place across several informal focus groups (see, Bennett et al., 2021 for more information). During study development, patients and the public were not involved in the choice of outcome measures, or recruitment for the study.

Participants of the study will be given opportunity to give feedback in person or anonymously at debrief, so as to improve the protocol for future trials.

Discussion and Future Research

For peer review only

Decentering is a helpful strategy to notice and interact with unpleasant inner experiences. Evidence suggests that the ability to use this strategy is continuously distributed in the population, with those at the higher end of the continuum reporting less anxiety and depression. Decentering also appears to be a malleable skill that is reinforced across a range of different psychological interventions. Selectively training this skill during adolescence might therefore delimit the negative impact of everyday psychological stressors. This has the potential to improve emotional wellbeing in the short term and mitigate anxiety and depression symptoms in the long-term. The current trial is an initial investigation to establish whether decentering skills can be selectively trained during adolescence and to characterise its impact on mental health outcomes. These findings may not only reveal some of the best ways to teach this skill but may provide estimates of effect sizes that can inform future research.

For peer review only

References

1 Kessler, R. C. *et al.* Age of onset of mental disorders: a review of recent literature. *Current opinion in psychiatry* 20, 359 (2007).

2 Wilde, S. *et al.* Mindfulness training in UK secondary schools: a multiple case study approach to identification of cornerstones of implementation. *Mindfulness* 10, 376-389 (2019).

3 Vostanis, P., Humphrey, N., Fitzgerald, N., Deighton, J. & Wolpert, M. How do schools promote emotional well-being among their pupils? Findings from a national scoping survey of mental health provision in E nglish schools. *Child and Adolescent Mental Health* 18, 151-157 (2013).

4 Weare, K. & Nind, M. Mental health promotion and problem prevention in schools: what does the evidence say? *Health promotion international* 26, i29-i69 (2011).

5 Teasdale, J. D. *et al.* Metacognitive awareness and prevention of relapse in depression: empirical evidence. *Journal of consulting and clinical psychology* 70, 275 (2002).

6 Bernstein, A. *et al.* Decentering and related constructs: A critical review and metacognitive processes model. *Perspectives on Psychological Science* 10, 599-617 (2015).

7 Bennett, M. P. *et al.* Decentering as a core component in the psychological treatment and prevention of youth anxiety and depression: A narrative review and insight report. *Translational Psychiatry* (2021).

8 Fresco, D. M. *et al.* Initial psychometric properties of the experiences questionnaire: validation of a self-report measure of decentering. *Behavior therapy* 38, 234-246 (2007).

For peer review only

9 Ayduk, Ö. & Kross, E. From a distance: implications of spontaneous self-distancing for adaptive self-reflection. *Journal of personality and social psychology* 98, 809 (2010).

10 White, R. E., Kross, E. & Duckworth, A. L. Spontaneous self-distancing and adaptive self-reflection across adolescence. *Child development* 86, 1272-1281 (2015).

11 Masuda, A., Hayes, S. C., Sackett, C. F. & Twohig, M. P. Cognitive defusion and self-relevant negative thoughts: Examining the impact of a ninety year old technique. *Behaviour research and therapy* 42, 477-485 (2004).

12 Masuda, A. *et al.* A parametric study of cognitive defusion and the believability and discomfort of negative self-relevant thoughts. *Behavior modification* 33, 250-262 (2009).

13 Nook, E. C., Vidal Bustamante, C. M., Cho, H. Y. & Somerville, L. H. Use of linguistic distancing and cognitive reappraisal strategies during emotion regulation in children, adolescents, and young adults. *Emotion* 20, 525 (2020).

14 Greco, L. A., Lambert, W. & Baer, R. A. Psychological inflexibility in childhood and adolescence: development and evaluation of the Avoidance and Fusion Questionnaire for Youth. *Psychological assessment* 20, 93 (2008).

15 Livheim, F. *et al.* Psychometric properties of the Avoidance and Fusion Questionnaire for Youth: A psychological measure of psychological inflexibility in youth. *Journal of Contextual Behavioral Science* 5, 103-110 (2016).

16 Muris, P. *et al.* Inflexible youngsters: Psychological and Psychopathological Correlates of the Avoidance and Fusion Questionnaire for youths in nonclinical Dutch adolescents. *Mindfulness* 8, 1381-1392 (2017).

For peer review only

17 Valdivia-Salas, S., Martín-Albo, J., Zaldivar, P., Lombas, A. S. & Jiménez, T. I. Spanish validation of the Avoidance and Fusion Questionnaire for Youth (AFQ-Y). *Assessment* 24, 919-931 (2017).

18 Teasdale, J. D., Segal, Z. & Williams, J. M. G. How does cognitive therapy prevent depressive relapse and why should attentional control (mindfulness) training help? *Behaviour Research and therapy* 33, 25-39 (1995).

19 Zettle, R. D., Rains, J. C. & Hayes, S. C. Processes of change in acceptance and commitment therapy and cognitive therapy for depression: A mediation reanalysis of Zettle and Rains. *Behavior modification* 35, 265-283 (2011).

20 Eustis, E. H., Hayes-Skelton, S. A., Orsillo, S. M. & Roemer, L. Surviving and thriving during stress: a randomized clinical trial comparing a brief web-based therapist-assisted acceptance-based behavioral intervention versus waitlist control for college students. *Behavior therapy* 49, 889-903 (2018).

21 Arch, J. J., Wolitzky-Taylor, K. B., Eifert, G. H. & Craske, M. G. Longitudinal treatment mediation of traditional cognitive behavioral therapy and acceptance and commitment therapy for anxiety disorders. *Behaviour research and therapy* 50, 469-478 (2012).

22 Twohig, M. P. *et al.* A randomized clinical trial of acceptance and commitment therapy versus progressive relaxation training for obsessive-compulsive disorder. *Journal of consulting and clinical psychology* 78, 705 (2010).

23 Bieling, P. J. *et al.* Treatment-specific changes in decentering following mindfulness-based cognitive therapy versus antidepressant medication or placebo for prevention of depressive relapse. *Journal of consulting and clinical psychology* 80, 365 (2012).

For peer review only

24 Josefsson, T., Lindwall, M. & Broberg, A. G. The effects of a short-term mindfulness based intervention on self-reported mindfulness, decentering, executive attention, psychological health, and coping style: examining unique mindfulness effects and mediators. *Mindfulness* 5, 18-35 (2014).

25 Orzech, K. M., Shapiro, S. L., Brown, K. W. & McKay, M. Intensive mindfulness training-related changes in cognitive and emotional experience. *The Journal of Positive Psychology* 4, 212-222 (2009).

26 Hayes-Skelton, S. A., Calloway, A., Roemer, L. & Orsillo, S. M. Decentering as a potential common mechanism across two therapies for generalized anxiety disorder. *Journal of consulting and clinical psychology* 83, 395 (2015).

27 Hayes-Skelton, S. A. & Graham, J. R. Decentering as a common link among mindfulness, cognitive reappraisal, and social anxiety. *Behavioural and cognitive psychotherapy* 41, 317 (2013).

28 Travers-Hill, E., Dunn, B. D., Hoppitt, L., Hitchcock, C. & Dalgleish, T. Beneficial effects of training in self-distancing and perspective broadening for people with a history of recurrent depression. *Behaviour research and therapy* 95, 19-28 (2017).

29 Powers, J. P. & LaBar, K. S. Regulating emotion through distancing: A taxonomy, neurocognitive model, and supporting meta-analysis. *Neuroscience & Biobehavioral Reviews* 96, 155-173 (2019).

30 Gregório, S., Pinto-Gouveia, J., Duarte, C. & Simões, L. Expanding research on decentering as measured by the Portuguese version of the experiences questionnaire. *The Spanish journal of psychology* 18, E23 (2015).

31 Naragon-Gainey, K. & DeMarree, K. G. Structure and validity of measures of decentering and defusion. *Psychological Assessment* 29, 935 (2017).

For peer review only

32 Lucena-Santos, P., Carvalho, S., Pinto-Gouveia, J., Gillanders, D. & Oliveira, M. S. Cognitive Fusion Questionnaire: Exploring measurement invariance across three groups of Brazilian women and the role of cognitive fusion as a mediator in the relationship between rumination and depression. *Journal of Contextual Behavioral Science* 6, 53-62 (2017).

33 Soler, J. *et al.* Assessing decentering: validation, psychometric properties, and clinical usefulness of the experiences questionnaire in a Spanish sample. *Behavior therapy* 45, 863-871 (2014).

34 Forman, E. M. *et al.* The Drexel defusion scale: A new measure of experiential distancing. *Journal of Contextual Behavioral Science* 1, 55-65 (2012).

35 Kobayashi, R., Shigematsu, J., Miyatani, M. & Nakao, T. Cognitive reappraisal facilitates decentering: A longitudinal cross-lagged analysis study. *Frontiers in psychology* 11, 103 (2020).

36 Hayes-Skelton, S. A. & Marando-Blanck, S. Examining the interrelation among change processes: Decentering and anticipatory processing across cognitive behavioral therapy for social anxiety disorder. *Behavior therapy* 50, 1075-1086 (2019).

37 Hayes-Skelton, S. A. & Lee, C. S. Changes in decentering across cognitive behavioral group therapy for social anxiety disorder. *Behavior therapy* 49, 809-822 (2018).

38 Mischkowski, D., Kross, E. & Bushman, B. J. Flies on the wall are less aggressive: Self-distancing "in the heat of the moment" reduces aggressive thoughts, angry feelings and aggressive behavior. *Journal of Experimental Social Psychology* 48, 1187-1191 (2012).

For peer review only

39 Kross, E., Duckworth, A., Ayduk, O., Tsukayama, E. & Mischel, W. The effect of self-distancing on adaptive versus maladaptive self-reflection in children. *Emotion* 11, 1032 (2011).

40 Ebesutani, C. *et al.* The Revised Child Anxiety and Depression Scale-Short Version: Scale reduction via exploratory bifactor modeling of the broad anxiety factor. *Psychological assessment* 24, 833 (2012).

41 Radloff, L. S. The CES-D scale: A self-report depression scale for research in the general population. *Applied psychological measurement* 1, 385-401 (1977).

42 Brunner, T. M. & Spielberger, C. D. *STAXI-2 C/A: State-Trait Anger Expression Inventory-2, Child and Adolescent: Professional Manual*. (Psychological Assessment Resources, 2009).

43 Garnefski, N. & Kraaij, V. The cognitive emotion regulation questionnaire. *European Journal of Psychological Assessment* 23, 141-149 (2007).

44 Gratz, K. L. & Roemer, L. Multidimensional assessment of emotion regulation and dysregulation: Development, factor structure, and initial validation of the difficulties in emotion regulation scale. *Journal of psychopathology and behavioral assessment* 26, 41-54 (2004).

45 Silva, W. A. D., de Sampaio Brito, T. R. & Pereira, C. R. COVID-19 anxiety scale (CAS): Development and psychometric properties. *Current Psychology*, 1-10 (2020).

46 Verbruggen, F. & De Houwer, J. Do emotional stimuli interfere with response inhibition? Evidence from the stop signal paradigm. *Cognition and emotion* 21, 391-403 (2007).

For peer review only

47 Blanke, O. *et al.* Linking out-of-body experience and self processing to mental own-body imagery at the temporoparietal junction. *Journal of Neuroscience* 25, 550-557 (2005).

48 Delespaul, P. A. Assessing schizophrenia in daily life: The experience sampling method. (1995).

49 Tennant, R., Hiller, L., Fishwick, R. et al. The Warwick-Edinburgh Mental Well-being Scale (WEMWBS): development and UK validation. Health Qual Life Outcomes 5, 63 (2007).

50 Greco, L. A., Baer, R. A., & Smith, G. T. (2011). Assessing mindfulness in children and adolescents: development and validation of the Child and Adolescent Mindfulness Measure (CAMM). Psychological Assessment, 23(3), 606–614.

For peer review only

Author Statement

- MB & RK – Substantial contributions to the conception, design of work, data acquisition, data analysis and interpretation, and manuscript preparation.
- TD, TF, WK, SJB, MW, GW – Substantial contributions to the conception and design of work.
- AAB, TS, DD, ED, PW, MV, BL, HC – Substantial contributions to data acquisition & analysis.

For peer review only

Figure Legends

- Figure 1 – A Schematic Overview of the Intervention Timeline
- Figure S1 – Affective Cognitive Control Task
- Figure S2 – Self Referential Processing Task

For peer review only

	STUDY PERIOD																																									
	IN-TAKE						INTERVENTION																																			POST-INTERVENTION
							WEEK 1							WEEK 2							WEEK 3							WEEK 4							WEEK 5							
	days	-5	-4	-3	-2	-1	1	2	3	4	5	6	7	8	9	10	11	12	13	14	15	16	17	18	19	20	21	22	23	24	25	26	27	28	29	30	31	32	33	34	35	
**Enrolment**																																										
Eligbility screening	•																																									
Alloaction	•																																									
Briefing & Consent	•																																									
ESM Tutorial	•																																									
**Intervention**																																										
Decentering training & Active control							•	•	•	•	•	•	•	•	•	•	•	•	•	•	•	•	•	•	•	•	•	•	•	•	•	•	•	•	•	•	•	•	•	•	•	
**Self-report assessments**																																										
ESM		•	•	•	•	•	•	•	•	•	•	•	•	•	•	•	•	•	•	•	•	•	•	•	•	•	•	•	•	•	•	•	•	•	•	•	•	•	•	•	•	
EQ (Decentering)		•																						•																		•
CES-D (Depression)		•																						•																		•
RCADS (Anxiety)		•																						•																		•
WEMWBS (Well-being)		•																						•																		•
STAXI (Anger)		•																						•																		•
SDQ (General Well-being)		•																																								•
CERQ (Emotion Regulation)		•																																								•
DERS (Emotion Regulation)		•																																								•
CAMM (Mindfulness)		•																																								•
ADI (Adoelscent Decentering)		•																																								•
CAS (COVID anxiety)		•																																								
Patient Expectation Item		•																																								
**Performance-based tasks**																																										
ESST (Attention and inhibitory control)		•																																								•
OBT (Self-perspective processing)		•																																								•

584x272mm (72 x 72 DPI)

Appendix 6 – Affective Cognitive Control Task

For example, an emotional Stop Signal Task.

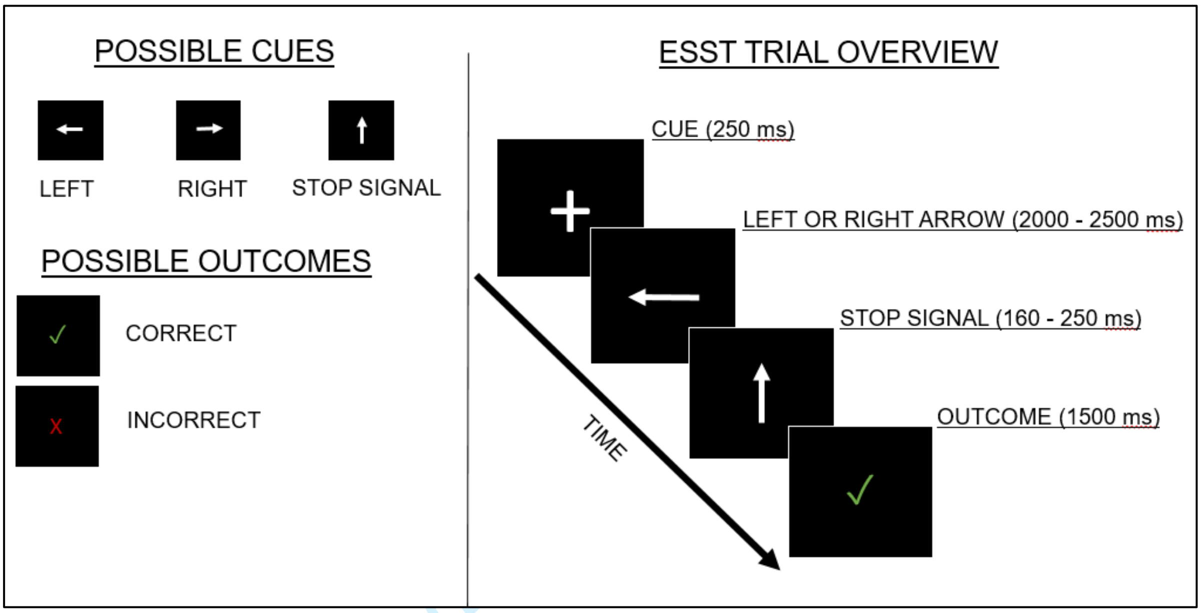

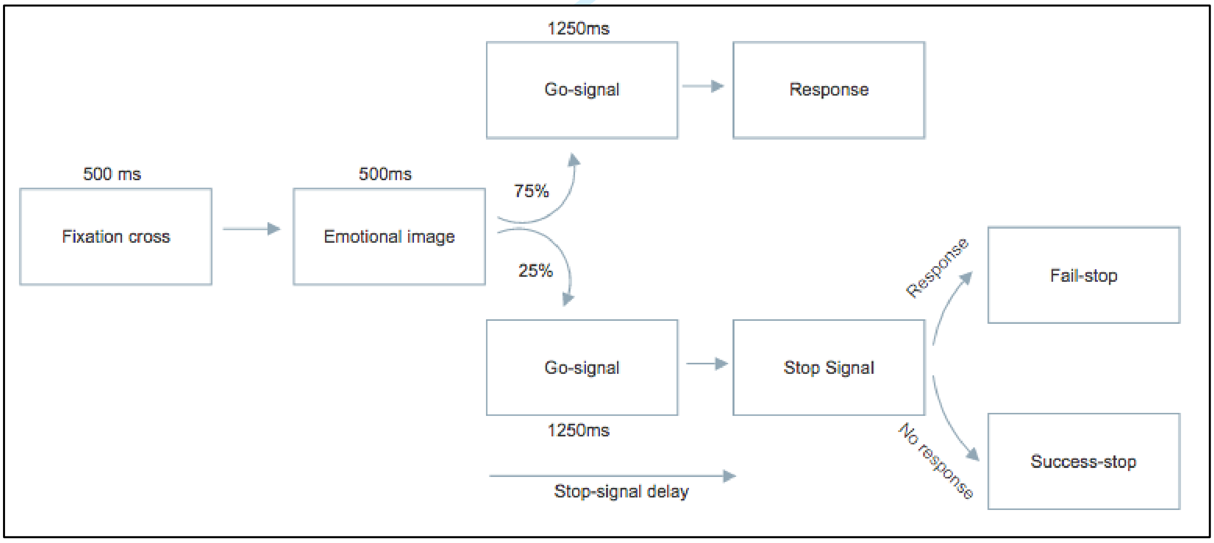

Across multiple trials, participants respond to a go-signal (e.g. press-left to a left arrow) unless it is followed by a stop-signal (an upwards arrow); these stop-signals follow 20% of go-signals. In the emotional stop-signal task (eSST) go-signals are preceded by a neutral or negative image from the IAPS (IAPS; Lang et al., 1992). We will measure accuracy and neurophysiological activities during response inhibition.

Appendix 2 – Self Referential Processing Task

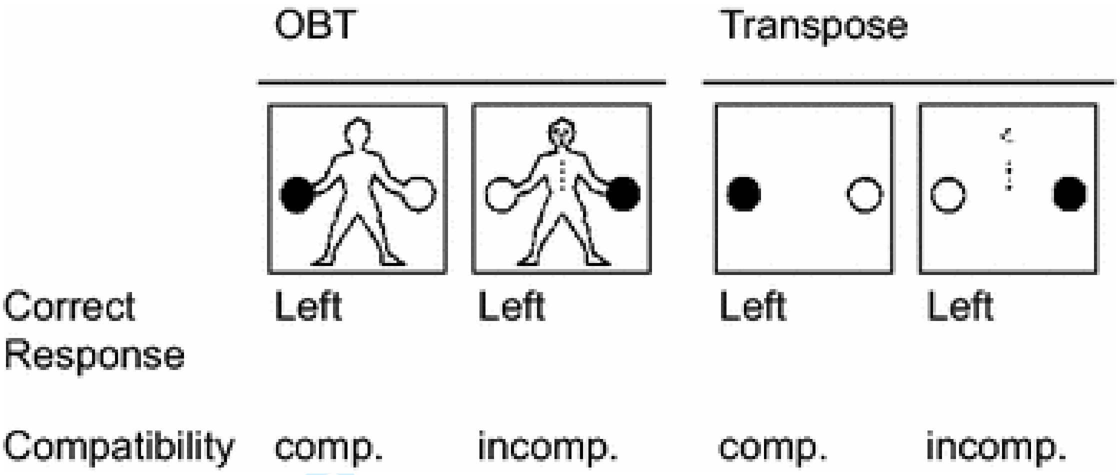

For example, the Own Body Task requires speeded spatial judgments made from the perspective of a schematic figure. The stimulus is a figure holding a black ball in one hand and a white ball in the other. The black ball was either in the figure's left or right hand and the figure could be facing either toward or away from the participant, but always in an upright orientation. Participants were instructed to imagine themselves in the body position of the figure, in order to judge whether the black ball was being held by the figure's left or right hand by pressing the corresponding response keys. We will measure how quickly participants are able to judge which hand the black ball is in, and whether they can correctly judge this.

Appendix A

MRC Cognition and Brain Sciences Unit
UNIVERSITY OF CAMBRIDGE

Tim Dalgleish
Principal Investigator
MRC Cognition and Brain Sciences Unit
University of Cambridge
15 Chaucer Road,
Cambridge, CB2 7EF
Phone: 01223 766166
myriad@mrc-cbu.cam.ac.uk

Participant Consent Form

Study title: Investigating the neuro-cognitive dynamics of decentring training and its impact on emotional reactivity: A Longitudinal and multimodal assessment.

We are interested in how new therapeutic techniques like mindfulness and decentring affect your thoughts and feelings.

This study has been reviewed by the Cambridge Psychology Research Ethics Committee [PRE.2019.036]

Please initial at the end of each line and then sign below to indicate that you agree to take part in the study:

• Read and understood the information sheet for this study

....

• Had the opportunity to consider the information and ask questions via the email address or phone number given at the top of this sheet

....

• Had your questions answered satisfactorily

....

• Understood that this project will require you to have an app installed on your smartphone for the duration of the project

....

• Understood that your participation is voluntary and that he or she can withdraw at any time without giving a reason

....

For peer review only

• Understood that your data and personal details will be stored on secure servers. At the end of the study, the data will be transferred and held at The MRC Cognition and Brain Sciences Unit in a secure database and will be accessed by the academic community

………………

• The MRC Cognition and Brain Sciences Unit will take all reasonable steps to protect your privacy. Your data will be coded and will not be associated with your name or information that could reveal your identity. This anonymised data will be made freely available to the wider academic community ……………

• Understood that all information will be treated as strictly confidential and handled in accordance with the provisions of the Data Protection Act 2018

……………

• Understood that in all instances where the results are disseminated they will remain anonymous

……………

PLEASE USE BLOCK CAPITAL LETTERS WHEN FILLING IN THIS FORM

Your name……
…………

Your school/college….………
…..................

Gender ……………………….. Year group………………………… Date of birth……………………………………….

Other information. Does you have any condition that may affect your performance on the tasks? If so please state: ………………………………………………………………….

Would you like to be contacted in the future about participating in other studies (please circle)?
YES NO

Your email………

Please sign below to indicate that you agree to take part in the study:

Your signature: …….. Date: ....

For peer review only

Daily_Diaries_Intervention

Start of Block: Intro

Q69 Please Enter Your ID Number

Remember, this is the last three letters of your phone number, then the last three characters of your postcode, then the last letter of your surname.

____

Q68 Thanks for your participation in our study so far. We have some questions about your day today that we'd like you to answer.

End of Block: Intro

Start of Block: Block 1

Q50 Thinking back over the day, please rate to what extent you have felt this mood (**happy**) today.

	1 - not at all (1)	2 (2)	3 (3)	4 (4)	5 (5)	6 (6)	7 - very much (7)
1 (1)	o	o	o	o	o	o	o

Q51 How much time in the day do you feel you have spent in this mood (**happy**).

	1 - very little (1)	2 (2)	3 (3)	4 (4)	5 (5)	6 (6)	7 - most of the day (7)
1 (1)	o	o	o	o	o	o	o

For peer review only - http://bmjopen.bmj.com/site/about/guidelines.xhtml

Page Break

For peer review only

For peer review only - http://bmjopen.bmj.com/site/about/guidelines.xhtml

Q52 Thinking back over the day, please rate to what extent you have felt this mood (**lively**) today.

	1 - not at all (1)	2 (2)	3 (3)	4 (4)	5 (5)	6 (6)	7 - very much (7)
1 (1)	○	○	○	○	○	○	○

Q53 How much time in the day do you feel you have spent in this mood (**lively**).

	1 - very little (1)	2 (2)	3 (3)	4 (4)	5 (5)	6 (6)	7 - most of the day (7)
1 (1)	○	○	○	○	○	○	○

Page Break

For peer review only

Q54 Thinking back over the day, please rate to what extent you have felt this mood (**content**) today.

	1 - not at all (1)	2 (2)	3 (3)	4 (4)	5 (5)	6 (6)	7 - very much (7)
1 (1)	○	○	○	○	○	○	○

Q55 How much time in the day do you feel you have spent in this mood (**content**).

	1 - very little (1)	2 (2)	3 (3)	4 (4)	5 (5)	6 (6)	7 - most of the day (7)
1 (1)	○	○	○	○	○	○	○

Page Break

For peer review only

For peer review only - http://bmjopen.bmj.com/site/about/guidelines.xhtml

Q56 Thinking back over the day, please rate to what extent you have felt this mood (**satisfied**) today.

	1 - not at all (1)	2 (2)	3 (3)	4 (4)	5 (5)	6 (6)	7 - very much (7)
1 (1)	○	○	○	○	○	○	○

Q57 How much time in the day do you feel you have spent in this mood (**satisfied**).

	1 - very little (1)	2 (2)	3 (3)	4 (4)	5 (5)	6 (6)	7 - most of the day (7)
1 (1)	○	○	○	○	○	○	○

Page Break

For peer review only

For peer review only - http://bmjopen.bmj.com/site/about/guidelines.xhtml

Q58 Thinking back over the day, please rate to what extent you have felt this mood (**depressed**) today.

	1 - not at all (1)	2 (2)	3 (3)	4 (4)	5 (5)	6 (6)	7 - very much (7)
1 (1)	○	○	○	○	○	○	○

Q59 How much time in the day do you feel you have spent in this mood (**depressed**).

	1 - very little (1)	2 (2)	3 (3)	4 (4)	5 (5)	6 (6)	7 - most of the day (7)
1 (1)	○	○	○	○	○	○	○

Page Break

For peer review only

For peer review only - http://bmjopen.bmj.com/site/about/guidelines.xhtml

Q60 Thinking back over the day, please rate to what extent you have felt this mood (**bored**) today.

	1 - not at all (1)	2 (2)	3 (3)	4 (4)	5 (5)	6 (6)	7 - very much (7)
1 (1)	○	○	○	○	○	○	○

Q61 How much time in the day do you feel you have spent in this mood (**bored**).

	1 - very little (1)	2 (2)	3 (3)	4 (4)	5 (5)	6 (6)	7 - most of the day (7)
1 (1)	○	○	○	○	○	○	○

Page Break

For peer review only

Q62 Thinking back over the day, please rate to what extent you have felt this mood (**anxious**) today.

	1 - not at all (1)	2 (2)	3 (3)	4 (4)	5 (5)	6 (6)	7 - very much (7)
1 (1)	○	○	○	○	○	○	○

Q63 How much time in the day do you feel you have spent in this mood (**anxious**).

	1 - very little (1)	2 (2)	3 (3)	4 (4)	5 (5)	6 (6)	7 - most of the day (7)
1 (1)	○	○	○	○	○	○	○

Page Break

For peer review only

For peer review only - http://bmjopen.bmj.com/site/about/guidelines.xhtml

Q64 Thinking back over the day, please rate to what extent you have felt this mood (**irritable**) today.

	1 - not at all (1)	2 (2)	3 (3)	4 (4)	5 (5)	6 (6)	7 - very much (7)
1 (1)	○	○	○	○	○	○	○

Q65 How much time in the day do you feel you have spent in this mood (**irritable**).

	1 - very little (1)	2 (2)	3 (3)	4 (4)	5 (5)	6 (6)	7 - most of the day (7)
1 (1)	○	○	○	○	○	○	○

Page Break

For peer review only

For peer review only - http://bmjopen.bmj.com/site/about/guidelines.xhtml

Q66 Thinking back over the day, please rate to what extent you have felt this mood (**tense**) today.

	1 - not at all (1)	2 (2)	3 (3)	4 (4)	5 (5)	6 (6)	7 - very much (7)
1 (1)	○	○	○	○	○	○	○

Q67 How much time in the day do you feel you have spent in this mood (**tense**).

	1 - very little (1)	2 (2)	3 (3)	4 (4)	5 (5)	6 (6)	7 - most of the day (7)
1 (1)	○	○	○	○	○	○	○

End of Block: Block 1

Start of Block: Block 2

Q41 Now, we just have some final questions...

Q42 Did you complete any of the exercises from this programme today?

○ Yes (1)

○ No (2)

Display This Question:

If Did you complete any of the exercises from this programme today? = Yes

For peer review only

Q43 How difficult did you find these exercises?

- 1 - Not at all difficult (2)
- 2 (3)
- 3 (4)
- 4 - Somewhat difficult (5)
- 5 (6)
- 6 (7)
- 7 - Extremely Difficult (8)

Display This Question:

If Did you complete any of the exercises from this programme today? = Yes

Q44
Did you find the skills you've been learning to be relevant at any stage during the day?

- 1 - Not At All Relevant (1)
- 2 (2)
- 3 (3)
- 4 - Somewhat (4)
- 5 (5)
- 6 (6)
- 7 - Extremely (7)

For peer review only

For peer review only - http://bmjopen.bmj.com/site/about/guidelines.xhtml

Display This Question:

If Did you complete any of the exercises from this programme today? = Yes

Q45 During the day, were you able to apply the skills you've learned during the exercises?

- ◯ 1 - Not At All (1)
- ◯ 2 (2)
- ◯ 3 (3)
- ◯ 4 - Somewhat (4)
- ◯ 5 (5)
- ◯ 6 (6)
- ◯ 7 - Absolutely (7)

Display This Question:

If Did you complete any of the exercises from this programme today? = Yes

Q49
During the day, how effortful was it to apply the skills you have learned ?

- ◯ 1- Not at all (1)
- ◯ 2 (2)
- ◯ 3 (3)
- ◯ 4 - Somewhat (4)
- ◯ 5 (5)
- ◯ 6 (6)
- ◯ 7 - Extremely effortful (7)

End of Block: Block 2

For peer review only

For peer review only

For peer review only - http://bmjopen.bmj.com/site/about/guidelines.xhtml

Reporting checklist for protocol of a clinical trial.

Based on the SPIRIT guidelines.

Instructions to authors

Complete this checklist by entering the page numbers from your manuscript where readers will find each of the items listed below.

Your article may not currently address all the items on the checklist. Please modify your text to include the missing information. If you are certain that an item does not apply, please write "n/a" and provide a short explanation.

Upload your completed checklist as an extra file when you submit to a journal.

In your methods section, say that you used the SPIRITreporting guidelines, and cite them as:

Chan A-W, Tetzlaff JM, Gøtzsche PC, Altman DG, Mann H, Berlin J, Dickersin K, Hróbjartsson A, Schulz KF, Parulekar WR, Krleža-Jerić K, Laupacis A, Moher D. SPIRIT 2013 Explanation and Elaboration: Guidance for protocols of clinical trials. BMJ. 2013;346:e7586

		Reporting Item	Page Number
**Administrative information**			
Title	#1	Descriptive title identifying the study design, population, interventions, and, if applicable, trial acronym	1
Trial registration	#2a	Trial identifier and registry name. If not yet registered, name of intended registry	1
Trial registration: data set	#2b	All items from the World Health Organization Trial Registration Data Set	1
Protocol version	#3	Date and version identifier	1
Funding	#4	Sources and types of financial, material, and other support	1
Roles and responsibilities: contributorship	#5a	Names, affiliations, and roles of protocol contributors	32

For peer review only

Roles and responsibilities: sponsor contact information	#5b	Name and contact information for the trial sponsor	1
Roles and responsibilities: sponsor and funder	#5c	Role of study sponsor and funders, if any, in study design; collection, management, analysis, and interpretation of data; writing of the report; and the decision to submit the report for publication, including whether they will have ultimate authority over any of these activities	N/A - Study Sponsor and Funders had no role
Roles and responsibilities: committees	#5d	Composition, roles, and responsibilities of the coordinating centre, steering committee, endpoint adjudication committee, data management team, and other individuals or groups overseeing the trial, if applicable (see Item 21a for data monitoring committee)	N/A - No Data Monitoring Committee was used
**Introduction**			
Background and rationale	#6a	Description of research question and justification for undertaking the trial, including summary of relevant studies (published and unpublished) examining benefits and harms for each intervention	1
Background and rationale: choice of comparators	#6b	Explanation for choice of comparators	12
Objectives	#7	Specific objectives or hypotheses	6
Trial design	#8	Description of trial design including type of trial (eg, parallel group, crossover, factorial, single group), allocation ratio, and framework (eg, superiority, equivalence, non-inferiority, exploratory)	7
**Methods: Participants, interventions, and outcomes**			

For peer review only

Study setting	#9	Description of study settings (eg, community clinic, academic hospital) and list of countries where data will be collected. Reference to where list of study sites can be obtained	8
Eligibility criteria	#10	Inclusion and exclusion criteria for participants. If applicable, eligibility criteria for study centres and individuals who will perform the interventions (eg, surgeons, psychotherapists)	8
Interventions: description	#11a	Interventions for each group with sufficient detail to allow replication, including how and when they will be administered	10
Interventions: modifications	#11b	Criteria for discontinuing or modifying allocated interventions for a given trial participant (eg, drug dose change in response to harms, participant request, or improving / worsening disease)	11
Interventions: adherance	#11c	Strategies to improve adherence to intervention protocols, and any procedures for monitoring adherence (eg, drug tablet return; laboratory tests)	11
Interventions: concomitant care	#11d	Relevant concomitant care and interventions that are permitted or prohibited during the trial	8
Outcomes	#12	Primary, secondary, and other outcomes, including the specific measurement variable (eg, systolic blood pressure), analysis metric (eg, change from baseline, final value, time to event), method of aggregation (eg, median, proportion), and time point for each outcome. Explanation of the clinical relevance of chosen efficacy and harm outcomes is strongly recommended	13
Participant timeline	#13	Time schedule of enrolment, interventions (including any run-ins and washouts), assessments, and visits for participants. A schematic diagram is highly recommended (see Figure)	18
Sample size	#14	Estimated number of participants needed to achieve study objectives and how it was	9

For peer review only

		determined, including clinical and statistical assumptions supporting any sample size calculations	
Recruitment	#15	Strategies for achieving adequate participant enrolment to reach target sample size	9
**Methods: Assignment of interventions (for controlled trials)**			
Allocation: sequence generation	#16a	Method of generating the allocation sequence (eg, computer-generated random numbers), and list of any factors for stratification. To reduce predictability of a random sequence, details of any planned restriction (eg, blocking) should be provided in a separate document that is unavailable to those who enrol participants or assign interventions	18
Allocation concealment mechanism	#16b	Mechanism of implementing the allocation sequence (eg, central telephone; sequentially numbered, opaque, sealed envelopes), describing any steps to conceal the sequence until interventions are assigned	18
Allocation: implementation	#16c	Who will generate the allocation sequence, who will enrol participants, and who will assign participants to interventions	18
Blinding (masking)	#17a	Who will be blinded after assignment to interventions (eg, trial participants, care providers, outcome assessors, data analysts), and how	19
Blinding (masking): emergency unblinding	#17b	If blinded, circumstances under which unblinding is permissible, and procedure for revealing a participant's allocated intervention during the trial	19
**Methods: Data collection, management, and analysis**			

For peer review only

Data collection plan	#18a	Plans for assessment and collection of outcome, baseline, and other trial data, including any related processes to promote data quality (eg, duplicate measurements, training of assessors) and a description of study instruments (eg, questionnaires, laboratory tests) along with their reliability and validity, if known. Reference to where data collection forms can be found, if not in the protocol	13
Data collection plan: retention	#18b	Plans to promote participant retention and complete follow-up, including list of any outcome data to be collected for participants who discontinue or deviate from intervention protocols	20
Data management	#19	Plans for data entry, coding, security, and storage, including any related processes to promote data quality (eg, double data entry; range checks for data values). Reference to where details of data management procedures can be found, if not in the protocol	21
Statistics: outcomes	#20a	Statistical methods for analysing primary and secondary outcomes. Reference to where other details of the statistical analysis plan can be found, if not in the protocol	21
Statistics: additional analyses	#20b	Methods for any additional analyses (eg, subgroup and adjusted analyses)	21
Statistics: analysis population and missing data	#20c	Definition of analysis population relating to protocol non-adherence (eg, as randomised analysis), and any statistical methods to handle missing data (eg, multiple imputation)	21
**Methods: Monitoring**			
Data monitoring: formal committee	#21a	Composition of data monitoring committee (DMC); summary of its role and reporting structure; statement of whether it is independent from the sponsor and competing interests; and reference to	21

For peer review only

		where further details about its charter can be found, if not in the protocol. Alternatively, an explanation of why a DMC is not needed	
Data monitoring: interim analysis	#21b	Description of any interim analyses and stopping guidelines, including who will have access to these interim results and make the final decision to terminate the trial	21
Harms	#22	Plans for collecting, assessing, reporting, and managing solicited and spontaneously reported adverse events and other unintended effects of trial interventions or trial conduct	22
Auditing	#23	Frequency and procedures for auditing trial conduct, if any, and whether the process will be independent from investigators and the sponsor	N/A - Small scale trial so data will not be audited
**Ethics and dissemination**			
Research ethics approval	#24	Plans for seeking research ethics committee / institutional review board (REC / IRB) approval	22
Protocol amendments	#25	Plans for communicating important protocol modifications (eg, changes to eligibility criteria, outcomes, analyses) to relevant parties (eg, investigators, REC / IRBs, trial participants, trial registries, journals, regulators)	22
Consent or assent	#26a	Who will obtain informed consent or assent from potential trial participants or authorised surrogates, and how (see Item 32)	19
Consent or assent: ancillary studies	#26b	Additional consent provisions for collection and use of participant data and biological specimens in ancillary studies, if applicable	33
Confidentiality	#27	How personal information about potential and enrolled participants will be collected, shared, and maintained in order to protect confidentiality before, during, and after the trial	20

For peer review only

Declaration of interests	#28	Financial and other competing interests for principal investigators for the overall trial and each study site	1
Data access	#29	Statement of who will have access to the final trial dataset, and disclosure of contractual agreements that limit such access for investigators	1
Ancillary and post trial care	#30	Provisions, if any, for ancillary and post-trial care, and for compensation to those who suffer harm from trial participation	20
Dissemination policy: trial results	#31a	Plans for investigators and sponsor to communicate trial results to participants, healthcare professionals, the public, and other relevant groups (eg, via publication, reporting in results databases, or other data sharing arrangements), including any publication restrictions	23
Dissemination policy: authorship	#31b	Authorship eligibility guidelines and any intended use of professional writers	23
Dissemination policy: reproducible research	#31c	Plans, if any, for granting public access to the full protocol, participant-level dataset, and statistical code	23
**Appendices**			
Informed consent materials	#32	Model consent form and other related documentation given to participants and authorised surrogates	33
Biological specimens	#33	Plans for collection, laboratory evaluation, and storage of biological specimens for genetic or molecular analysis in the current trial and for future use in ancillary studies, if applicable	N/A - None collected

Notes:

- 5c: N/A - Study Sponsor and Funders had no role
- 5d: N/A - No Data Monitoring Committee was used
- 23: N/A - Small scale trial so data will not be audited

For peer review only

- 33: N/A - None collected The SPIRIT Explanation and Elaboration paper is distributed under the terms of the Creative Commons Attribution License CC-BY-NC. This checklist was completed on 08. July 2021 using https://www.goodreports.org/, a tool made by the EQUATOR Network in collaboration with Penelope.ai

For peer review only

BMJ Open

Protocol for a randomized controlled trial investigating an intervention to boost decentering in response to distressing mental experiences during adolescence: The Decentering in Adolescence Study (DECADES)

Journal:	*BMJ Open*
Manuscript ID	bmjopen-2021-056864.R2
Article Type:	Protocol
Date Submitted by the Author:	23-Feb-2022

Complete List of Authors:	Bennett, Marc; MRC Cognition and Brain Sciences Unit<br>Knight, Rachel; MRC Cognition and Brain Sciences Unit,<br>Dunning, Darren; University of Cambridge,<br>Archer-Boyd, Alan; MRC Cognition and Brain Sciences Unit<br>Blakemore, Sarah-Jayne; University of Cambridge,<br>Dalmaijer, Edwin; MRC Cognition and Brain Sciences Unit<br>Ford, Tamsin; University of Cambridge, Psychiatry<br>Williams, J Mark; University of Oxford, Psychiatry<br>Clegg, Hannah; MRC Cognition and Brain Sciences Unit<br>Kuyken, Willem; University of Oxford, Psychiatry<br>So, Tierney; University of Cambridge<br>Wright, Gem; MRC Cognition and Brain Sciences Unit<br>Lenaert, Bert; Maastricht University, Faculty of Healh, Medicine and Life Sciences<br>Vainre, Maris; Cambridge University, MRC Cognition and Brain Sciences Unit<br>Watson, Peter; University of Cambridge, MRC Cognition and Brain Sciences Unit<br>Dalgleish, Tim; Medical Research Council Cognition and Brain Sciences Unit,

**Primary Subject Heading**:	Mental health
Secondary Subject Heading:	Evidence based practice
Keywords:	Child & adolescent psychiatry < PSYCHIATRY, Anxiety disorders < PSYCHIATRY, Depression & mood disorders < PSYCHIATRY, Clinical trials < THERAPEUTICS, PREVENTIVE MEDICINE

SCHOLARONE™
Manuscripts

Protocol for a randomized controlled trial investigating an intervention to boost decentering in response to distressing mental experiences during adolescence: The Decentering in Adolescence Study (DECADES)

Bennett, M.P.[1]*, Knight, R. [1]*, Dunning, D.[1], Archer-Boyd, A.[1], Blakemore, S-J., Dalmaijer, E.[1], Ford, T.[2], Williams, J. M. G.[3], Clegg, H.[1], Kuyken, W.[3], So, T.[1], Wright, G.[1], Lenaert, B.[4,5,6], Vainre, M. [1], Watson, P.,[1] MYRIAD team, & Dalgleish, T. [1,7]

1- Medical Research Council- Cognition and Brain Sciences Unit, University of Cambridge, UK.

2- Department of Psychiatry, University of Cambridge, UK.

3- Department of Psychiatry, University of Oxford, UK.

4- Faculty of Health, Medicine and Life Sciences, Maastricht University, Maastricht, NL.

5- Limburg Brain Injury Centre, Limburg, NL.

6- Faculty of Psychology, Open University, Heerlen, the Netherlands

7- Cambridgeshire and Peterborough NHS Foundation Trust

*co-first authors

Trial number: ISRCTN14329613

Version: 4.3

Correspondence for this article should be addressed to Miss Rachel Knight or Dr. Marc Bennett, Medical Research Council- Cognition and Brain Sciences Unit, 15 Chaucer

For peer review only

Road, Cambridge, CB2 7EF. E-mail: Rachel.knight@mrc-cbu.cam.ac.uk, marc.bennett@mrc-cbu.cam.ac.uk

Data Statement

The datasets generated during and/or analysed during the current study will be stored in a publically available repository. Following the publication of the first manuscript, a fully anonymized dataset will be made available online on open-access databases whose servers are located within the UK or Europe. Data processing scripts and programming codes will also be made available online.

Conflict of Interest Statement

The authors declare no competing interests.

Funding

This project is funded by a Wellcome Strategic Award (Wellcome Trust, ref 104908/Z/14/z; awarded to TD, SJB, FT, WK, MW). The contribution of MB was partially supported by a Wellcome Trust Active Ingredients in Mental Heatlh Commission. RK is funded by an Economic and Social Research Council Doctoral Fellowship (ref SUAI/067).

Keywords

Decentering, adolescence, psychological distress, intervention, ecological momentary assessment

For peer review only

The **MYRIAD** Team comprises Saz Ahmed, PhD, of University College London, Susan Ball, MSc, of University of Exeter, Nicola Dalrymple, MSc, of University of Oxford, Katie Fletcher, HSD, of University of Oxford, Lucy Foulkes, PhD, of University College London, Poushali Ganguli, MSc, of Kings College London, Cait Griffin, MSc, Kirsty Griffiths, MSc, of University of Cambridge, Konstantina Komninidou, BEd, of University of Oxford, Suzannah Laws, BSc, of University of Oxford, Jovita Leung, MSc, of University College London, Jenna Parker, MSc, of University of East Anglia, Blanca Piera Pi-Sunyer, MSc, of University College London, J. Ashok Sakhardande, BSc Hons, Jem Shackleford, MA, MSc, Kate Tudor, PhD, of University of Oxford, and Brian Wainman, BEng, of Plymouth University. These individuals have worked across the MYRIAD strategic award "Promoting Mental Health and Building Resilience in Adolescence: Investigating Mindfulness and Attentional Control"; they are acknowledged as group authors in this article for their substantial contributions to the project development, in accordance with the MYRIAD Dissemination Protocol

For peer review only

Abstract

Introduction. Decentering describes the ability to voluntarily adopt an objective self-perspective from which to notice internal, typically distressing, stressors (e.g. difficult thoughts, memories, and feelings). The reinforcement of this skill may be an active ingredient through which different psychological interventions accrue reductions in anxiety and/or depression. However, it is unclear if decentering can be selectively trained at a young age and if this might reduce psychological distress. The aim of the current trial is to address this research gap. **Methods and analysis.** Adolescents, recruited from schools in the UK and the EU (n = 57 per group, age range = 16-19 years), will be randomised to complete five-weeks of decentering training, or an active control group that will take part in a combination of light physical exercise and cognitive training. The co-primary training outcomes include a self-reported decentering inventory (i.e. the Experiences Questionnaire) and the momentary use of decentering in response to psychological stressors, using experience sampling. The secondary mental health outcomes will include self-reported inventories of depression and anxiety symptoms, as well as psychological wellbeing. Initial statistical analysis will use between-group analysis of covariance to estimate the effect of training condition on self-rated inventories, adjusted for baseline scores. Additionally, experience sampling data will be examined using hierarchical linear models. **Ethics and dissemination.** This study was approved by the Cambridge Psychology Research Ethics Committee, University of Cambridge (PRE.2019.109). Findings will be disseminated through typical academic routes including poster/paper presentations at (inter)-national conferences, academic institutes and through publication in peer-reviewed journals.

Trial registration number: ISRCTN14329613

For peer review only

Article Summary

Strengths and limitations of this study

Strengths.

- We aim to strengthen an active and pan-therapeutic skill that features across different psychological interventions, and may improve well-being in adolescents at high risk of mental health difficulty.

- The intervention will be highly accessible by capitalising on extant social media and streaming services.

- Ecological momentary assessment will facilitate a temporally sensitive investigation into the effects of decentering training in everyday life.

Limitations.

- A lack of face-to-face interaction between experimenters and participants may undermine adherence.

- The study may be demanding as it requires daily, albeit brief, engagement over a several weeks.

For peer review only

Introduction

Background

Anxiety and depression are a considerable public health challenge. Since these difficulties often begin before the age of 24, responding to this challenge requires a special focus on adolescence[1]. It has been suggested that an effective way to promote early mental health is through universal approaches[2-4]; these are interventions that can be offered across a broad community of young people, irrespective of symptom severity. The goal of such universal approaches is to not only reduce distress in young people experiencing symptoms, but also to prevent future anxiety and depression onset in those who may be asymptomatic. However, their effectiveness depends on how well an intervention focuses on psychological experiences and mechanisms that are relevant across the entire spectrum of mental health, from risk to resilience, and through to flourishing. One strategy is therefore to target widely relevant therapeutic skills that can help manage distressing inner experiences (e.g. unpleasant feelings, thoughts, memories), which can otherwise trigger short-term distress and long-term risk of anxiety and depression onset. Psychological decentering, or decentering for short, may be one such skill.

Psychological decentering is a key concept within psychological therapy and science[5]. It is characterised as an adaptive self-observation style wherein one can attend to, not just the mental content of distressing inner experiences, but also its underlying cognitive nature[6,7]. Thus, decentering relates to how a person interacts with the difficult inner events prompted by experiences in daily life – it involves the ability to generate an objective self-perspective wherein one can notice mental experiences as imperfect models of the real-world rather than precise reflections. For example, a person might notice "I am thinking I am depressed right now" instead of only noticing

For peer review only

and then believing the thought "I am depressed"[8]. Decentering resultantly restricts the disproportionate influence psychological stressors may have on affect, behaviour and sense-of-self. Indeed, brief therapeutic exercises that stimulate a decentred self-perspective have been shown to reduce negative emotional reactivity towards stressors like unpleasant memories and negative self-relevant statements[9-13]. Evidence also indicates self-reported decentering traits in adolescence are associated with fewer overall symptoms of anxiety and depression[14-17].

Decentering is a malleable skill that can be refined through psychological intervention[7]. Medium to large changes in self-reported decentering are reported in adults experiencing anxiety and depression following a range of psychological interventions. This includes cognitive behavioural therapy (CBT)[18,19], acceptance based approaches[20-22] and mindfulness training[23-25]. In fact, emerging evidence suggests that intervention-related increases in decentering are a common pathway through which different psychological interventions deliver reductions in anxiety and depression[21,26,27]. This has encouraged some investigations into whether or not decentering can be trained selectively so to improve mental health. For example, Travers-Hill et al. trained adults with a diagnosis of recurrent depression currently in remission but experiencing residual depression symptoms to practice self-distancing techniques in response to difficult autobiographical memories[28]. This resulted in a significant increase in self-reported measures of decentering relative to an active comparison group who learned strategies to manage maladaptive avoidance tendencies. Additionally, those who learned to apply this decentering-related technique reported less distress towards negative memories at post-intervention even in the absence of instructions to self-distance.

For peer review only

Therapeutic exercises that target decentering skills are already nested within different psychological interventions[7]. Emerging evidence also indicates that these components can be delivered in isolation so to selectively boost decentering and reduce risk of relapse in formerly depressed adults[28]. However, a remaining question is 'can decentering skills be selectively reinforced during adolescents, and if so, through what therapeutic techniques?' This is the primary research question guiding the current study. It is also unclear if boosting adolescent decentering skills might impact on emotion and mental health. The current study will therefore evaluate, as a secondary question, how decentering training impacts anxiety, depression and emotional reactivity in adolescents. This information will inform future research trials and ultimately help in the investigation of effective universal approaches in youth mental health. A straightforward way to address these questions might be to examine the impact of decentering training on trait-level measure of decentering and symptom severity at critical time points, e.g. pre and post-intervention design. However, this approach lacks the sensitivity required to detect subtle changes in decentering in response to momentary psychological stressors and their impact on emotion. A design that also includes ecological momentary assessment or experience sampling methods (ESM) could therefore facilitate a more ecological investigation of the use of decentering in everyday life as well as its impact on transient psychological stressors and emotional states.

Current study objectives

The primary aim of the current study is to investigate if decentering can be improved during adolescence via an intensive decentering training programme. This will be investigated (1a) at a trait level by examining changes in self-reported experience of decentering (via a standard decentering inventory) and (1b) at a

For peer review only

situational level by examining self-reported use of decentering skills in response to momentary psychological stressors (via ESM assessments). Here, repeated measures of participants' negative emotional reactivity towards momentary inner experiences (e.g. feelings, thoughts and memories) will be recorded over a five day baseline period and across the duration of the intervention. A secondary aim is to explore potential mental health outcomes associated with reinforced decentering skills. This will be investigated (2a) at the symptom level by examining relative change in self-rated anxiety, depression and psychological well-being and (2b) at the situational level by recording emotional reactivity towards momentary psychological stressors using ESM. A final exploratory aim is to investigate some of the cognitive correlates of decentering and training. Thus, this study will answer the following research questions: (1a) Is decentering training during adolescence associated with increased decentering reports relative to an active control condition? (1b) Is decentering training during adolescence associated with increased decentering in response to negative mental events relative to an active control condition? (2a) What is the impact of decentering training on youth mental health relative to an active control condition? (2b) What is the impact of decentering training on emotional reactivity towards momentary negative mental events relative to an active control? (3) What are the cognitive correlates of decentering training during adolescence?

This study will address these research questions in a cohort of adolescents who are at increased risk of depression. This is a first step towards our long-term goal to develop a universal intervention that targets psychological experiences and mechanisms that are broadly relevant in young people. Adolescents experiencing elevated symptoms of depression will be recruited from both the UK (e.g. sixth form colleges) and EU (e.g. secondary level education high schools) and will be randomly

For peer review only

assigned to a five week decentering training programme or an active control. Both programmes will be delivered remotely via extant social media platforms; this means they can be delivered at low-cost, are easily accessible and are flexible regarding other commitments of our participants. The decentering intervention comprises 10-15 minute of audio exercises to be made available each weekday through music/podcast streaming services, e.g. *Spotify*, *iTunes*, *Deezer*, *Podcast Addict.* Participants will practice a specific decentering technique each week, which has been adapted from either the literature or an extant psychological therapy. Participants will also practice brief mindfulness-based grounding exercises each week to encourage the monitoring of inner experiences like emotional thoughts, memories and feelings. The overall goal of this programme is therefore to teach adolescents different ways to generate an objective self-perspective from which they can interact with negative psychological events encountered in daily life. Rather than rehearsing decentering techniques, the active control condition comprises 10-15 minutes of gamified cognitive tasks for completion on a personal smartphone. In addition, participants in this condition will be given short physical movement routines to complete in lieu of mindfulness-based grounding.

Methods

Study design

The design is a randomised controlled feasibility trial (comparing a psychological decentering training to an active control programme) with school aged adolescents (aged 16-19 years). The study will run from December 2021 until July 2022, split into several cohorts. Self-rated inventories of training and mental health outcomes will be assessed at three time points: within one week prior to the start of

For peer review only

programme (baseline); within the third week of training (mid-intervention); and one week after the programme has finished (post-intervention).

Participants and inclusion/exclusion criteria

Two groups of older adolescents (n = 57 per group; age range = 16–19 years) will be recruited from the UK and Ireland. Eligible participants must consent to completing (1) a five day ESM baseline assessment, (2) five weeks of the assigned training programme and (3) assessment measures at baseline, mid-intervention and post-intervention. In exchange for their time, participants will be compensated with shopping vouchers worth (1) £50 (for completing ESM measures), (2) £100 pounds (for completing five weeks of training) and (3) £50 (as a bonus for completing all assessments). Eligible participants must also report a score of 16 or above on the Centre for Epidemiological Studies-Depression Scale (CES-D), based on standard CES-D cut-offs. Participants must also have access to a laptop/desktop computer and a personal smartphone device.

Participants will be excluded if they: (1) currently take part in a regular (once or more per week) yoga and/or mindfulness class/workshop, (2) have participated in prior formal meditation training or a mindfulness-based stress reduction course (3) are currently experiencing chronic illness (e.g. epilepsy, chronic pain, cancer), (4) lack fluency in English (5) have a recent diagnosis of, and are currently receiving medical/psychological treatment for, a mental health condition including (but not limited to) anxiety disorder, major depressive disorder or a traumatic stress disorder and (6) have a diagnosis of a neurodevelopmental condition such as Autism Spectrum Disorder or Attention Deficit/Hyperactivity Disorder. Both the decentering programme and active control programme require adolescents to be without severe hearing difficulties since both conditions require listening to audiotapes. As the control

For peer review only

condition involves physical movement routines it may not be appropriate for specific individuals, such as individuals with limited mobility. Any volunteer who meets the exclusion criteria will be accommodated as best as possible (e.g. assignment to the most appropriate condition in the case of a physical disability) and their data will not be included in the analysis.

Recruitment

Participants will be recruited from the general community through a combination of existing collaborations with schools in the UK and Ireland. We will also recruit UK and Irish volunteers via online research platforms (e.g. Prolific; www.prolific.co) as well as extant panels of adolescent research volunteers via the MRC-Cognition and Brain Sciences Unit (University of Cambridge). Finally, and if necessary, we will also conduct a targeted recruitment campaign to recruit young people and schools using posters, pamphlets and online adverts on social media.

Sample size

The primary training outcome is self-rated decentering and this will be assessed using both a popular inventory of decentering (Experiences Questionnaire; EQ[8]) and ESM items. There is no consensus on how to best conduct a statistical power analysis for ESM data. Power and sample calculations were therefore based on the effect of training condition on EQ scores. A power analysis calculated in G*Power indicated that a total sample size of 90 is powered at 80% to observe a significant effect of training condition (decentering vs. control) on post-intervention decentering with a medium effect size of f = 0.3 (alpha = .05) after adjusting for baseline EQ-score. A medium effect size is plausible given that previous research reports a medium–large effect of extant psychological intervention on EQ scores. An attrition rate of around 20% is anticipated based on our previous research. Therefore, with 57 participants in

For peer review only - http://bmjopen.bmj.com/site/about/guidelines.xhtml

each group, the current study is adequately powered to observe a medium to large effect of decentering training on our candidate training outcome measures and to determine the nature of this interaction. Regarding our secondary mental health outcomes (i.e. anxiety, depression and psychological wellbeing), a sample size of 57 per group is consistent with our previous research to evaluate the impact of novel psychological interventions. That is, such group sizes can provide a reasonable range of point estimates of the effect on mental health outcome measures that are sufficient to guide later research; for example, fifty-seven participants per group is powered at 88% to observe a significant ($p < .05$) main effect of group (decentering versus active control) on baseline adjusted mental health outcomes with an effect size $f = 0.26$.

Intervention

Decentering Training.

A five week psychological decentering training programme was developed by MB, RK and TD, based on our previous protocol (Travers-Hill et al., 2017). This involves audio-recorded scripts and an accompanying work book that guides participants through four types of decentering technique; this structure is partly based on a recent taxonomy of self-distancing (a construct closely related to decentering)[29]. The decentering techniques include: (Week 1) *spatial distancing* wherein individuals are taught to re-imagine negative memories from a physically distant perspective (e.g. 'replay the memory but as if you're a fly on the wall'); (Week 2) *verbal distancing/cognitive defusion* wherein individuals are taught to re-phrase negative self-relevant statements in a way that challenges its literal value and influence over affective behaviour (e.g. replacing first person pronouns with one's name); (Week 3) *temporal distancing* wherein individuals are taught to re-consider specific worries from a temporally distant future (e.g. 'how would this seem in 5 years?'); and (Week 4)

objective distancing wherein individuals are taught to adopt a third-person perspective towards negative memories (e.g. ‘what is the effect of reliving a difficult memory from the perspective of an objective observer?). Week 5 is a revision week, during which participants will be encouraged to practice a different technique from weeks 1 to 4 each day. These techniques were selected since they are directly targeted at how adolescents relate to and observe day-to-day psychological stressors. Specifically, the goal is to teach adolescents concrete ways to generate an objective (or distanced) self-perspective in response to everyday feelings, thoughts and memories that are unpleasant. We assume this training will develop participants’ decentering ability above their baseline levels.

Each week will involve 5 audio-recorded exercises (10-15 minutes) that will be made available using common streaming services. One exercise will be posted each weekday, Monday to Friday. The first two exercises (Monday and Tuesday) are brief mindfulness grounding exercises designed to promote open monitoring of psychological experiences. The next three exercises (Wednesday to Friday) are decentering training exercises as described above. Adherence to the programme will be encouraged by directly contacting the participants in week 1 and week 3 to discuss their experiences so far and allow troubleshooting. Participant engagement will be monitored by reviewing the number of completed workbook exercise at the end of the five weeks. Participants will also complete daily diaries. These will include five questions about the intervention and its application, such as “Did you complete any of the programme exercises today?” or “During the day, were you able to apply the skills you've learned from the exercises?” (see Appendix A). A participant’s engagement in the trial will be discontinued if they elect to withdraw their participation or if they

For peer review only - http://bmjopen.bmj.com/site/about/guidelines.xhtml

experience serious physical/mental health difficulties that necessitate medical or psychological intervention.

Physical and Cognitive Exercise.

A five week active control programme was developed (MB, RK, and TD). This contains two elements that roughly match the decentering training for time and cognitive engagement. First, guided physical movement routines will be completed in lieu of mindfulness grounding exercises (Monday-Tuesday). These movements are intended to emulate the physiological nature of grounding exercises but without an internal focus. Participants will watch short videos in which one member of the research team (RK) illustrates a basic series of body stretches. Accompanying audio provides additional direction and this was recorded using the same voices from the decentering training programme. Each video comprises 15 stretches, with each stretch held for 30 seconds and a 10 second break between stretches. The physical movements were selected for their ease and accessibility. Care was also taken to select movements that are safe and cater to a range of physical abilities.

Second, gamified versions of standard cognitive tasks will be completed in lieu of the decentering training (Wednesday-Friday). These games are intended to emulate the cognitive effort associated with decentering exercises. Each participant will have a link that allows them to select one of three games, which can be completed on a personal smart phone (for game outlines, see https://osf.io/aw6c5). Games include: (1) a Multi-Target Visual Search Task wherein participants search and respond to specific targets within a broader stimulus array; (2) a Go/NoGo Task wherein a speeded response is made in response to a ‘go’ signal but inhibited in response to a ’no go’ signal: and (3) a Digit Recall Task wherein participants must recall a sequence on a number pad (i.e. a digit-span task). Adherence to the programme will be

For peer review only - http://bmjopen.bmj.com/site/about/guidelines.xhtml

encouraged by contacting the participants in week 1 and week 3 to discuss their experiences. Engagement will be monitored by reviewing task completion rates and performance measures (response times and accuracy) at the end of the five weeks.

Outcomes

There are two types of outcome measures in this study (see Figure 1 for Timeline). These include measures of (1) decentering and (2) mental health. We will also examine if there are any far-transfer effects of decentering training by including measures of (3) cognitive performance. Figure 1 provides a schematic overview of the assessment timeline.

Decentering outcome measures.

The primary training outcome measure is self-rated decentering as measured using the experiences questionnaire (EQ). A secondary decentering outcome measure is (2) the self-rated use of psychological decentering in response to psychological stressors as measured using ESM. These measures have been included to address research questions 1a and 1b, respectively.

Experiences questionnaire. This widely-used self-report assessment features 11 items that explore an individual's tendency to psychologically decenter from difficult subjective experiences in day-to-day life (e.g. 'I can observe unpleasant feelings without being drawn into them')[8]. Items are answered using a 5-point Likert scale from 1 = 'Never' to 5 = 'All the time'). Psychometric properties were found to be satisfactory (Cronbach's α=.893; convergent validity r>.46)[33]. Scores on the EQ are positively associated with other decentering-related constructs like experiential avoidance (a tendency to attempt to evade difficult thoughts and feelings)[30-34] and cognitive reappraisal (an ability to reconceptualise situations to modify emotional impact)[27,35]. Finally, EQ scores improve over the course of behavioural and cognitive therapies; the

For peer review only

magnitude of such change is associated with key outcomes like symptom severity and quality-of-life[23,26,27,36,37]. The EQ will be administered at baseline, mid-intervention and post-intervention. We expect mean EQ scores will increase in the decentering training group relative to the active control condition.

ESM decentering items. All ESM items are described in Table 1 (see overleaf). Some items relate primarily to the use of decentering skills. Other ESM items relate to secondary mental health outcomes and are described in the next section. However, all ESM items will be administered during each sample point. Two ESM items have been developed to estimate the momentary use of decentering in response to psychological stressors like difficult feelings, memories or thoughts. These are "*Since the last beep, I was able to distance myself from unpleasant feelings*" and "*Since the last beep, I was able to distance myself from unpleasant things on my mind*". Items are answered using a 7-point Likert scale ranging from 1 = 'Not at all' to 7 = 'Very much'). ESM items will be delivered four times daily across a five day ESM baseline period and the five weeks of training (see Figure 1). All ESM items are described in Table 1 (see overleaf). We expect momentary reports of decentering to increase over time in the decentering training group relative to the active control condition.

For peer review only

Table 1. Experience Sampling items

Item	Question	Rating	Purpose	Conditional
1	Right now, I feel…	-5 = Very Negative, 5 = Very Positive	Affect	Always Shown
2	I Feel:		Feelings/Mood	Always Shown
i	*Happy*	1 = Not at all, 7 = Very Much	Feelings/Mood	Always Shown
ii	*Relaxed*	1 = Not at all, 7 = Very Much	Feelings/Mood	Always Shown
iii	*Satisfied*	1 = Not at all, 7 = Very Much	Feelings/Mood	Always Shown
iv	*Enthusiastic*	1 = Not at all, 7 = Very Much	Feelings/Mood	Always Shown
v	*Nervous*	1 = Not at all, 7 = Very Much	Feelings/Mood	Always Shown
vi	*Sad*	1 = Not at all, 7 = Very Much	Feelings/Mood	Always Shown
vii	*Irritated*	1 = Not at all, 7 = Very Much	Feelings/Mood	Always Shown
viii	*Stressed*	1 = Not at all, 7 = Very Much	Feelings/Mood	Always Shown
3	Since the last beep, I felt sucked in by negative feelings?	1 = Not at all, 7 = Very Much	ER | Immersion negative feelings	Always Shown
4	Since the last beep, I was able to distance myself from negative feelings?	1 = Not at all, 7 = Very Much	ER | Distance negative feelings	Always Shown
5	Since the last beep, I tried to distract myself from negative feelings?	1 = Not at all, 7 = Very Much	ER | Distraction	Always Shown
6	Since the last beep, I noticed an unpleasant thought or memory?	1 = Not at all, 7 = Very Much	Thoughts/memories onset	Always Shown
7	Since the last beep, I was upset by an unpleasant thought or memory?	1 = Not at all, 7 = Very Much	Thoughts/memories onset	Always Shown
8	The unpleasant thing on my mind was about …	Myself, Others, A combination of these, None of these	Content- self/other	Contingent | (if Q6 != 1)
9	The unpleasant thing on my mind was about …	Social things, Professional things, A combination of these, None of these	Content- social/non-social	Contingent | (if Q6 != 1)
10	The unpleasant thing on my mind was about …	The past, The present, The future, A combination, None of these	Content- past/future	Contingent | (if Q6 != 1)
12	Since the last beep, I felt sucked in by unpleasant things on my mind.	1 = Not at all, 7 = Very Much	ER | Immersion in thoughts/memories	Always Shown
13	Since the last beep, I able to distance myself from unpleasant things on my mind.	1 = Not at all, 7 = Very Much	ER | Distance from thoughts/memories	Always Shown
14	Since the last beep, I tried to think differently about things so to feel better.	1 = Not at all, 7 = Very Much	ER | Re-appraisal	Always Shown
15	Since the last beep, I tried to distract myself from unpleasant things in my mind.	1 = Not at all, 7 = Very Much	ER | Distract	Always Shown
16	Since the last beep, I feel I benefited from the daily programme.	1 = Not at all, 7 = Very Much	Usefulness	Always Shown
Branch 1	Right now, I am …	Indoors, Outdoors	Branch	Contingent | (if Q6 == 1)
Branch 2	Right now, I am …	Alone, With others	Branch	Contingent | (if Q6 == 1)
Branch 3	Since the last beep, I felt social.	1 = Not at all, 7 = Very Much	Branch	Contingent | (if Q6 == 1)

For peer review only - http://bmjopen.bmj.com/site/about/guidelines.xhtml

Secondary mental health outcomes.

Our secondary mental health outcomes are (1) self-rated anxiety, depression and psychological well-being and (2) self-rated emotional reactivity following a negative mental experience (e.g. unpleasant thought, feeling or memory) as measured using ESM. These measures have been included to address research questions 2a and 2b.

Mental health symptoms. This study will explore the potential impact of decentering training on mental health outcomes at baseline, mid-intervention and post-intervention. Previous findings suggest that decentering is negatively associated with anxiety and depression [14-16] as well as feelings of anger in adolescents[38,39]. We will therefore include measures of each of these difficulties. Anxiety symptoms will be assessed using the Revised Child Anxiety and Depression Scale-Short Version (RCADS-15)[40]. The RCADS-SV is a 15 item scale measuring the reported frequency of various symptoms of anxiety and low mood. Internal consistency has been reported as good (Cronbach's α = .7-.96), as were test-retest coefficients and convergent validity. Depression symptoms will be assessed using the Centre for Epidemiology Depression Scale (CES-D)[41]. The CES-D is a 20 item inventory measuring depressive symptoms experienced in the past week. Internal consistency is good, with Cronbach's α = .85-.90. Concurrent and construct validity have been demonstrated. Anger will be measured using the State-Trait Anger Expression Inventory-2 Child and Adolescent (STAXI-2)[42], which is a 35 item self-report measuring anger expression and control in adolescents. The STAXI exhibits good reliability, with Cronbach's alpha coefficients ranging from .81 to .93, with good convergent validity.

Two measures will also be included to estimate the impact of decentering training on psychosocial strengths/difficulties and general wellbeing. The Strengths

and Difficulties Questionnaire (SDQ) is a screening tool with 5 subscales (Emotional Symptoms, Conduct Problems, Hyperactivity/Inattention, Peer Relationships, and Prosocial Behaviour) as well as an impact supplement to estimate psycho-social functioning. This tool was included to capture behavioural symptoms such as conduct difficulties and hyperactivity. The SDQ exhibits strong internal consistency, moderate test-retest reliability and good concurrent validity. Psychological wellbeing will be measured using the Warwick-Edinburgh Mental Wellbeing Scale (WEMWBS)[43]. The WEMWBS is a 14 item measure of positive mental health including positive affect, functioning and inter-personal relationships. The WEMWBS shows high internal consistency, and good convergent and construct validity. Measures will be administered at baseline, mid-intervention and post-intervention.

ESM emotional reactivity. This study will examine if the lagged effect of the decentering from difficult thoughts, feelings or memories (time = n) on later affect (time = n + 1) changes across training. One ESM items will capture general affect: 'Right now I feel…', answered on a 11 point Likert scale ranging from -5 = Very negative to +5 =Very positive. Two separate ESM items will capture the self-initiated use of decentering skills in response to negative mental experiences (Table **1**. These are: 'Since the last message, I was able to distance myself from [a] a negative feeling *or* [b] unpleasant things on my mind'. Additional ESM items will check for the occurrence of negative mental experiences. These include checks of (a) negative feelings by asking '*I feel Nervous/Sad/Irritated/Stressed*' and (b) unpleasant cognitive events by asking '*Since the last beep, I was upset by a thought or memory*'. This will be scored on a 7 point Likert scale from 1 = Not at all to 7 = Very Much.

One item will assess the occurrence of unpleasant thoughts: 'since the last bee, I noticed an unpleasant thought or memory'. This will allow us to control for the

For peer review only - http://bmjopen.bmj.com/site/about/guidelines.xhtml

presence of unpleasant cognitive events from which participants can decenter. If the participant's response is anything other than 'not at all', they will then complete three items to the content of these thought (Table 1, items 8-10). If the participant's response is 'not at all', they will complete three unrelated 'branching' items (Table 1, branch items 1-3). This means the same number of item will always be administered.

Additional self-rated assessments. Participants will complete several additional self-rated assessments for the purpose of exploratory analysis. Participants will also complete a brief end of day questionnaire that will include a measure of mood, assessing presence and duration of different mood states. Nine moods will be investigated– happy, lively, content, satisfied, depressed, bored, anxious, irritable and tense. These items will allow for exploratory analysis of how individuals' moods may relate to the two experimental conditions. The end of day questionnaire will also include items investigating whether participants have completed any exercises from their programme (decentering or control), how difficult these exercises were to complete, and whether these skills were relevant to their everyday life. This section is included both as a measure of adherence to the programme, and as a broad measure of participant feedback.

Our team is currently developing an adolescent friendly self-rated decentering inventory. A provisional version of this scale will be included at pre- and post-intervention as part of its on-going validation. We are also investigating the relationship between decentering and other emotion regulation skills. We will therefore include: the Cognitive Emotion Regulation Questionnaire (CERQ)[44], which is a 36 item questionnaire cataloguing a range of emotion regulation strategies (Cronbach's α = .62-.85); the Difficulties in Emotion Regulation Scale (DERS)[45], which is a 36-item questionnaire investigating problems with emotion regulation (Cronbach's α = .94),

For peer review only

including 6 subscales: non-acceptance of emotional responses; difficulty in engaged in goal-directed behaviour; impulse control difficulties; lack of emotional awareness; limited access to emotion regulation strategies; and lack of emotional clarity: and the Child and Adolescent Mindfulness Measure (CAMM)[46], which is a 10 item measure exploring trait mindfulness in children and adolescents (Cronbach's α = .88). Participants will complete the Coronavirus Anxiety Scale[47] scales at baseline and post-intervention. This may help us address any unexpected impact of the COVID-19 pandemic on the intervention. To control for impact of patient expectation on intervention efficacy, a single item patient expectation scale will also be administered at baseline.

Far transfer effects.

This study will include performance-based tasks to explore potential far-transfer effects of decentering training on cognition (research question 3). These include (1) affective cognitive control and (2) self-perspective processing, both of which have been posited as theoretical components of decentering[6 29].These measures will be administered at baseline and post-intervention.

Affective cognitive control. This study will explore if decentering training influences the ability to maintain cognitive performance within emotional contexts (i.e. affective cognitive control). This can be estimated via the Emotional Stop Signal Task (ESST; Figure S1)[48]. On each trial, a neutral or negative valence image is presented before a 'go-signal' that requires a speeded button-press (75% of trials). Participants must quickly make the appropriate button-press unless the 'go-signal' is followed by a 'stop-signal (25%). Participants must withhold their response on these trials. The period between the go and stop-signal (stop signal delay; SSD) varies over the task. Following a successful stop trial, the task is made more difficult by increasing the SSD

For peer review only

by 25 ms. Following an unsuccessful stop trial, the task is made easier by decreasing the SSD by 25 m. This tracking algorithm typically causes a failure to stop on ~50% of trials. Sustained attention in emotional and non-emotional contexts will be estimated by calculating (1) the reaction time (RT) mean on go-trials and (2) the inter-trial variability coefficient (i.e. the RT mean divided by the RT standard deviation). Inhibitory control in emotional and non-emotional contexts will estimated by calculating the *stop signal reaction time* (*i*.e. mean RT – mean SSD).

Self-referential processing. This study will explore if decentering training influences the ability to flexibly shift one's self-perspective. This can be indexed using the own-body transformation task (OBT; Figure S2)[49]. On each trial, a schematic human figure is presented that face either with their front to the participant or their back to the participant. This figure also holds a black glove in one hand (e.g. left). Participants are instructed to imagine themselves in the body position of the figure and then judge whether the glove is held by the figures left of right hand. Performance is compared to a within-subject control task wherein participants judge the position of the glove without altering their self-representation. The outcome variables are mean RT and the percentage error rate. These are found to increase as a function of the self-perspective manipulation; that is, they are higher for front-facing characters.

Procedure

Data quality.

Checks of data quality will be included ad different stages of data collection and analysis. First, 'catch questions' will be embedded in self-report assessments to assess whether participants actively read questions (e.g. '[describe one]'). Second, participants self-rate the quality of their data at the end of each data collection period, e.g. []. Piloting revealed this to be a useful strategy. Third, all the standard markers of

For peer review only

appropriate behavioural performance will be checked when analysing task data (e.g. reaction times >250 ms and <3000 ms; chance-level accuracy).

Assignment of interventions.

Allocation. Following baseline assessment, participants will be stratified according to sex and depression severity (using the CES-D). They will then be randomly assigned to either the decentering training programme or the active control condition. This will be managed by the trial statistician (PW) using a minimization procedure. Participant allocation will be then shared with the research coordinator responsible for providing appropriate access links for the online intervention (MB and RK).

Blinding. Research coordinators (MB and RK) will be blinded during assessment, data pre-processing and statistical analysis. Due to the nature of the study, it is not possible to remain blinded during participant allocation. This is because each participant needs to be given a secure link to the online training programme, either decentering training or the active control. All outcome assessments will be completed online; participants will access the same link to complete online assessments, regardless of group. Thus, there are no in-person data collectors with an awareness of participants' allocation. Initial data will be pre-processed (e.g. calculation of summary scores like means, percentage accuracy, sums etc.). Pre-processing will be completed using pre-existing scripts coded in R and MATLAB (MB). At this point, the dataset will be shared with the trial statistician (PW) who will conduct the statistical analysis and who will be blind to participant allocation. Once the analysis is complete, the trial coordinators will un-blind the dataset by indicating which participants were allocated to which group.

Consent and Data Collection.

For peer review only

Recruited volunteers will complete an eligibility check during an initial phone call. Volunteers who are eligible will be invited to participate in the study. Written informed consent will be sought after an initial briefing that explains the data collection protocol (see Appendix B for consent form). This briefing will also include a tutorial on downloading and using the experience sampling app (PsyMate2; www.psymate.eu). Participants will also have the opportunity to ask any questions. To discourage possible contamination, participants will be asked to keep the content of the study confidential from others. Once consent is provided, participants will be given an anonymous trial identification code and added to an encrypted list for allocation. Participants will also receive a URL link to complete baseline measures. Figure 1 illustrates the full schedule for outcome measures. The two-week ESM baseline assessment will also begin. At the end of this period, participants will receive a tutorial on accessing the intervention materials.

Over five weeks, participants in both interventions will access and rehearse exercises (Monday-Friday). Decentering training exercises are based on audio files to be delivered via their preferred music/post-streaming services (e.g. Spotify/Podcast Addict). The active control exercises are based on YouTube videos posted on a Private Channel. Each day, the PsyMate2 App will prompt participants to complete their daily exercise as well as complete any required ESM items. These will be sent four times per day. A brief survey will also be sent through the PsyMate2 App each evening to ask whether participants completed their daily exercises, and if so, to ask about their experiences as well as questions on their mood states (Supplemental Materials). At the end of Week 5, participants will be asked to complete the online assessment battery. Participants will then complete a debriefing session. During the debriefing session they will be given the opportunity to provide feedback on their

For peer review only

experience of the study, and to ask any questions they may have. They will also be given a list of relevant mental health resources should they feel the need to access them.

Participant Retention & Follow Up. Retention will be promoted in a number of ways. Firstly, participants will not be paid until receipt of their completed post-intervention assessment battery. Secondly, participants will be made aware that they can receive a bonus payment of £50 if they complete all of their assessments and daily diaries. Thirdly, telephone contact will be made with participants during week 1 and 3 to allow any troubleshooting and ensure that participants are filling in diaries and experience sampling appropriately. Telephone contact will also be made with a participant should they fail to complete mid-intervention assessments (Week 3).

Data Management and Statistical Analysis

Data Confidentiality and Management.

All participants will be given an anonymous trial identification code to use when submitting their completed assessments. A file linking the participant's name to their identification code will be saved in an encrypted and password protected file and stored on a secure, protected server within the MRC-Cognition and Brain Sciences Unit. This file will be deleted once the final round of data collection is complete. Outcome data will be gathered remotely using online platforms (e.g. Qualtrics; www.qualtrics.com) and an ESM data collection app. During periods of data collection, these data will be transferred to in-house secure servers within the MRC-Cognition and Brain Sciences Unit, University of Cambridge. The source data will be then deleted. Access to these data will be restricted to primary research team members (MB, RK, TD). Data management will be overseen by these team members. There will be no formal Data Management Committee (DMC) because it is a small scale trial.

For peer review only

Statistical Analysis.

Self-rated and performance-based measures. The statistical analysis will be conducted by the trial statistician (PW) who will be blind to training condition. The analysis will be conducted on an intention-to-treat basis and a similar initial analysis is planned for (i) the primary training outcome (i.e. self-rated EQ scores) and (ii) secondary mental health outcomes (i.e. CES-D, RCADS, STAXI-2, SDQ and WEMWBS). Analysis of covariance (ANCOVA) will be calculated to estimate the effect of training condition on outcome measures after adjusting for baseline performance and grouping stratification variables. This model will be calculated for our primary end-point, i.e. post-intervention (i.e. post-intervention outcome adjusted for baseline score). This model will additionally be calculated for our mid-intervention time point (i.e. mid-intervention outcome adjusted for baseline score) and post-intervention. There are no planned interim-analysis. If a subset of items of a measure (≤ 20% within-measure missing data), a total score will be calculated using the mean score across the non-missing items; an approach we have reported elsewhere (www.osf.io/d6y9q). Otherwise, the score for that measure will be noted as missing. We will assume that

data will be missing at random and address this using standard imputation approaches in R (R Core Team, 2013).

ESM analysis. Only participants who completed at least 33% of the ESM questionnaires will be included in the analyses, in line with ESM guidelines[50]. Statistical analyses will be performed using hierarchical linear models. These models are especially suited to deal with dependency inherent in the data due their hierarchical structure: ESM observations (level 1) nested within individuals (level 2). Potential confounders will be included in the model as covariates. First, a linear growth model will be estimated to assess the effect of condition (decentering training vs. active control) on decentering skills over time (i.e., the duration of the intervention). Second, time-lagged analyses will be performed to evaluate if higher self-rated decentering skills when confronted with a momentary psychological stressor (time = n) predict significantly lower negative affect ratings at a later time point (time = n + 1). We will also control for momentary negative affect ratings (at time = n). Random intercepts and random slopes models will be estimated. In hierarchical linear models, the fixed effects reflect the overall association between a predictor and the outcome of interest, whereas the random effects reflect individual differences in this association.

Ethics and Dissemination

Ethical Approval and Protocol Amendments.

This study was approved by the Cambridge Psychology Research Ethics Committee, University of Cambridge (PRE.2019.109). Approval for any protocol amendments will be sought by this committee. This includes methodological and/or trial management amendments. The online registration of the trial will also be updated in this situation and any amendments will be outlined in the trial manuscripts. This

For peer review only

committee will also be contacted should we encounter any unexpected ethical concerns.

Dissemination.

The findings of this study will be disseminated through typical academic routes including poster/paper presentations at (inter)-national conferences, academic institutes and through publication in peer-reviewed journals. Given that some research questions are less exploratory than others, two key manuscripts are planned for the trial data. The first will describe the impact of training on self-rated decentering and mental health outcomes. The second will describe the exploratory research around the impact on cognitive performance measures. Findings will also be disseminated to the broader public through seminars and workshops with relevant stakeholders as well as online blogs and podcasts. These manuscripts will be completed and submitted for review irrespective of the nature of the observed effects. Following the publication of the first manuscript, a fully anonymized dataset will be made available online on open-access databases whose servers are located within the UK or Europe. Data processing scripts and programming codes will also be made available online.

Patient and Public Involvement

A youth advisory panel was formed from May to December 2020. This panel advised on the accessibility of self-rated measures of decentering. They also provided qualitative feedback on decentering as an emotional regulation stratagem and the potential for decentering training. This took place across several informal focus groups (see, Bennett et al., 2021 for more information). During study development, patients and the public were not involved in the choice of outcome measures, or recruitment for the study.

For peer review only - http://bmjopen.bmj.com/site/about/guidelines.xhtml

Participants of the study will be given opportunity to give feedback in person or anonymously at debrief, so as to improve the protocol for future trials.

Discussion and Future Research

Decentering is a helpful strategy to notice and interact with unpleasant inner experiences. Evidence suggests that the ability to use this strategy is continuously distributed in the population, with those at the higher end of the continuum reporting less anxiety and depression. Decentering also appears to be a malleable skill that is reinforced across a range of different psychological interventions. Selectively training this skill during adolescence might therefore delimit the negative impact of everyday psychological stressors. This has the potential to improve emotional wellbeing in the short term and mitigate anxiety and depression symptoms in the long-term. The current trial is an initial investigation to establish whether decentering skills can be selectively trained during adolescence and to characterise its impact on mental health outcomes. These findings may not only reveal some of the best ways to teach this skill but may provide estimates of effect sizes that can inform future research.

For peer review only

References

1 Kessler, R. C. *et al.* Age of onset of mental disorders: a review of recent literature. *Current opinion in psychiatry* 20, 359 (2007).

2 Wilde, S. *et al.* Mindfulness training in UK secondary schools: a multiple case study approach to identification of cornerstones of implementation. *Mindfulness* 10, 376-389 (2019).

3 Vostanis, P., Humphrey, N., Fitzgerald, N., Deighton, J. & Wolpert, M. How do schools promote emotional well-being among their pupils? Findings from a national scoping survey of mental health provision in E nglish schools. *Child and Adolescent Mental Health* 18, 151-157 (2013).

4 Weare, K. & Nind, M. Mental health promotion and problem prevention in schools: what does the evidence say? *Health promotion international* 26, i29-i69 (2011).

5 Teasdale, J. D. *et al.* Metacognitive awareness and prevention of relapse in depression: empirical evidence. *Journal of consulting and clinical psychology* 70, 275 (2002).

6 Bernstein, A. *et al.* Decentering and related constructs: A critical review and metacognitive processes model. *Perspectives on Psychological Science* 10, 599-617 (2015).

7 Bennett, M. P. *et al.* Decentering as a core component in the psychological treatment and prevention of youth anxiety and depression: A narrative review and insight report. *Translational Psychiatry* (2021).

8 Fresco, D. M. *et al.* Initial psychometric properties of the experiences questionnaire: validation of a self-report measure of decentering. *Behavior therapy* 38, 234-246 (2007).

For peer review only

9 Ayduk, Ö. & Kross, E. From a distance: implications of spontaneous self-distancing for adaptive self-reflection. *Journal of personality and social psychology* 98, 809 (2010).

10 White, R. E., Kross, E. & Duckworth, A. L. Spontaneous self-distancing and adaptive self-reflection across adolescence. *Child development* 86, 1272-1281 (2015).

11 Masuda, A., Hayes, S. C., Sackett, C. F. & Twohig, M. P. Cognitive defusion and self-relevant negative thoughts: Examining the impact of a ninety year old technique. *Behaviour research and therapy* 42, 477-485 (2004).

12 Masuda, A. *et al.* A parametric study of cognitive defusion and the believability and discomfort of negative self-relevant thoughts. *Behavior modification* 33, 250-262 (2009).

13 Nook, E. C., Vidal Bustamante, C. M., Cho, H. Y. & Somerville, L. H. Use of linguistic distancing and cognitive reappraisal strategies during emotion regulation in children, adolescents, and young adults. *Emotion* 20, 525 (2020).

14 Greco, L. A., Lambert, W. & Baer, R. A. Psychological inflexibility in childhood and adolescence: development and evaluation of the Avoidance and Fusion Questionnaire for Youth. *Psychological assessment* 20, 93 (2008).

15 Livheim, F. *et al.* Psychometric properties of the Avoidance and Fusion Questionnaire for Youth: A psychological measure of psychological inflexibility in youth. *Journal of Contextual Behavioral Science* 5, 103-110 (2016).

16 Muris, P. *et al.* Inflexible youngsters: Psychological and Psychopathological Correlates of the Avoidance and Fusion Questionnaire for youths in nonclinical Dutch adolescents. *Mindfulness* 8, 1381-1392 (2017).

For peer review only

17 Valdivia-Salas, S., Martín-Albo, J., Zaldivar, P., Lombas, A. S. & Jiménez, T. I. Spanish validation of the Avoidance and Fusion Questionnaire for Youth (AFQ-Y). *Assessment* 24, 919-931 (2017).

18 Teasdale, J. D., Segal, Z. & Williams, J. M. G. How does cognitive therapy prevent depressive relapse and why should attentional control (mindfulness) training help? *Behaviour Research and therapy* 33, 25-39 (1995).

19 Zettle, R. D., Rains, J. C. & Hayes, S. C. Processes of change in acceptance and commitment therapy and cognitive therapy for depression: A mediation reanalysis of Zettle and Rains. *Behavior modification* 35, 265-283 (2011).

20 Eustis, E. H., Hayes-Skelton, S. A., Orsillo, S. M. & Roemer, L. Surviving and thriving during stress: a randomized clinical trial comparing a brief web-based therapist-assisted acceptance-based behavioral intervention versus waitlist control for college students. *Behavior therapy* 49, 889-903 (2018).

21 Arch, J. J., Wolitzky-Taylor, K. B., Eifert, G. H. & Craske, M. G. Longitudinal treatment mediation of traditional cognitive behavioral therapy and acceptance and commitment therapy for anxiety disorders. *Behaviour research and therapy* 50, 469-478 (2012).

22 Twohig, M. P. *et al.* A randomized clinical trial of acceptance and commitment therapy versus progressive relaxation training for obsessive-compulsive disorder. *Journal of consulting and clinical psychology* 78, 705 (2010).

23 Bieling, P. J. *et al.* Treatment-specific changes in decentering following mindfulness-based cognitive therapy versus antidepressant medication or placebo for prevention of depressive relapse. *Journal of consulting and clinical psychology* 80, 365 (2012).

For peer review only

24 Josefsson, T., Lindwall, M. & Broberg, A. G. The effects of a short-term mindfulness based intervention on self-reported mindfulness, decentering, executive attention, psychological health, and coping style: examining unique mindfulness effects and mediators. *Mindfulness* 5, 18-35 (2014).

25 Orzech, K. M., Shapiro, S. L., Brown, K. W. & McKay, M. Intensive mindfulness training-related changes in cognitive and emotional experience. *The Journal of Positive Psychology* 4, 212-222 (2009).

26 Hayes-Skelton, S. A., Calloway, A., Roemer, L. & Orsillo, S. M. Decentering as a potential common mechanism across two therapies for generalized anxiety disorder. *Journal of consulting and clinical psychology* 83, 395 (2015).

27 Hayes-Skelton, S. A. & Graham, J. R. Decentering as a common link among mindfulness, cognitive reappraisal, and social anxiety. *Behavioural and cognitive psychotherapy* 41, 317 (2013).

28 Travers-Hill, E., Dunn, B. D., Hoppitt, L., Hitchcock, C. & Dalgleish, T. Beneficial effects of training in self-distancing and perspective broadening for people with a history of recurrent depression. *Behaviour research and therapy* 95, 19-28 (2017).

29 Powers, J. P. & LaBar, K. S. Regulating emotion through distancing: A taxonomy, neurocognitive model, and supporting meta-analysis. *Neuroscience & Biobehavioral Reviews* 96, 155-173 (2019).

30 Gregório, S., Pinto-Gouveia, J., Duarte, C. & Simões, L. Expanding research on decentering as measured by the Portuguese version of the experiences questionnaire. *The Spanish journal of psychology* 18, E23 (2015).

31 Naragon-Gainey, K. & DeMarree, K. G. Structure and validity of measures of decentering and defusion. *Psychological Assessment* 29, 935 (2017).

For peer review only

32 Lucena-Santos, P., Carvalho, S., Pinto-Gouveia, J., Gillanders, D. & Oliveira, M. S. Cognitive Fusion Questionnaire: Exploring measurement invariance across three groups of Brazilian women and the role of cognitive fusion as a mediator in the relationship between rumination and depression. *Journal of Contextual Behavioral Science* 6, 53-62 (2017).

33 Soler, J. *et al.* Assessing decentering: validation, psychometric properties, and clinical usefulness of the experiences questionnaire in a Spanish sample. *Behavior therapy* 45, 863-871 (2014).

34 Forman, E. M. *et al.* The Drexel defusion scale: A new measure of experiential distancing. *Journal of Contextual Behavioral Science* 1, 55-65 (2012).

35 Kobayashi, R., Shigematsu, J., Miyatani, M. & Nakao, T. Cognitive reappraisal facilitates decentering: A longitudinal cross-lagged analysis study. *Frontiers in psychology* 11, 103 (2020).

36 Hayes-Skelton, S. A. & Marando-Blanck, S. Examining the interrelation among change processes: Decentering and anticipatory processing across cognitive behavioral therapy for social anxiety disorder. *Behavior therapy* 50, 1075-1086 (2019).

37 Hayes-Skelton, S. A. & Lee, C. S. Changes in decentering across cognitive behavioral group therapy for social anxiety disorder. *Behavior therapy* 49, 809-822 (2018).

38 Mischkowski, D., Kross, E. & Bushman, B. J. Flies on the wall are less aggressive: Self-distancing "in the heat of the moment" reduces aggressive thoughts, angry feelings and aggressive behavior. *Journal of Experimental Social Psychology* 48, 1187-1191 (2012).

For peer review only

39 Kross, E., Duckworth, A., Ayduk, O., Tsukayama, E. & Mischel, W. The effect of self-distancing on adaptive versus maladaptive self-reflection in children. *Emotion* 11, 1032 (2011).

40 Ebesutani, C. *et al.* The Revised Child Anxiety and Depression Scale-Short Version: Scale reduction via exploratory bifactor modeling of the broad anxiety factor. *Psychological assessment* 24, 833 (2012).

41 Radloff, L. S. The CES-D scale: A self-report depression scale for research in the general population. *Applied psychological measurement* 1, 385-401 (1977).

42 Brunner, T. M. & Spielberger, C. D. *STAXI-2 C/A: State-Trait Anger Expression Inventory-2, Child and Adolescent: Professional Manual*. (Psychological Assessment Resources, 2009).

43 Tennant, R., Hiller, L., Fishwick, R. et al. The Warwick-Edinburgh Mental Well-being Scale (WEMWBS): development and UK validation. Health Qual Life Outcomes 5, 63 (2007).

44 Garnefski, N. & Kraaij, V. The cognitive emotion regulation questionnaire. *European Journal of Psychological Assessment* 23, 141-149 (2007).

45 Gratz, K. L. & Roemer, L. Multidimensional assessment of emotion regulation and dysregulation: Development, factor structure, and initial validation of the difficulties in emotion regulation scale. *Journal of psychopathology and behavioral assessment* 26, 41-54 (2004).

46 Greco, L. A., Baer, R. A., & Smith, G. T. (2011). Assessing mindfulness in children and adolescents: development and validation of the Child and Adolescent Mindfulness Measure (CAMM). Psychological Assessment, 23(3), 606–614.

For peer review only - http://bmjopen.bmj.com/site/about/guidelines.xhtml

47 Silva, W. A. D., de Sampaio Brito, T. R. & Pereira, C. R. COVID-19 anxiety scale (CAS): Development and psychometric properties. *Current Psychology*, 1-10 (2020).

48 Verbruggen, F. & De Houwer, J. Do emotional stimuli interfere with response inhibition? Evidence from the stop signal paradigm. *Cognition and emotion* 21, 391-403 (2007).

49 Blanke, O. *et al.* Linking out-of-body experience and self processing to mental own-body imagery at the temporoparietal junction. *Journal of Neuroscience* 25, 550-557 (2005).

50 Delespaul, P. A. Assessing schizophrenia in daily life: The experience sampling method. (1995).

For peer review only

Author Statement

- MB & RK – Substantial contributions to the conception, design of work, data acquisition, data analysis and interpretation, and manuscript preparation.
- TD, TF, WK, SJB, MW, GW – Substantial contributions to the conception and design of work.
- AAB, TS, DD, ED, PW, MV, BL, HC – Substantial contributions to data acquisition & analysis.

For peer review only

Figure Legends

- Figure 1 – A Schematic Overview of the Intervention Timeline
- Figure S1 – Affective Cognitive Control Task
- Figure S2 – Self Referential Processing Task

For peer review only

	STUDY PERIOD																																									
	IN-TAKE						INTERVENTION																																			POST-INTERVENTION
							WEEK 1							WEEK 2							WEEK 3							WEEK 4							WEEK 5							
	days	-5	-4	-3	-2	-1	1	2	3	4	5	6	7	8	9	10	11	12	13	14	15	16	17	18	19	20	21	22	23	24	25	26	27	28	29	30	31	32	33	34	35	
**Enrolment**																																										
Eligbility screening	•																																									
Alloaction	•																																									
Briefing & Consent	•																																									
ESM Tutorial	•																																									
**Intervention**																																										
Decentering training & Active control							•	•	•	•	•	•	•	•	•	•	•	•	•	•	•	•	•	•	•	•	•	•	•	•	•	•	•	•	•	•	•	•	•	•	•	
**Self-report assessments**																																										
ESM		•	•	•	•	•	•	•	•	•	•	•	•	•	•	•	•	•	•	•	•	•	•	•	•	•	•	•	•	•	•	•	•	•	•	•	•	•	•	•	•	
EQ (Decentering)		•																						•																		•
CES-D (Depression)		•																						•																		•
RCADS (Anxiety)		•																						•																		•
WEMWBS (Well-being)		•																						•																		•
STAXI (Anger)		•																						•																		•
SDQ (General Well-being)		•																																								•
CERQ (Emotion Regulation)		•																																								•
DERS (Emotion Regulation)		•																																								•
CAMM (Mindfulness)		•																																								•
ADI (Adoelscent Decentering)		•																																								•
CAS (COVID anxiety)		•																																								
Patient Expectation Item		•																																								
**Performance-based tasks**																																										
ESST (Attention and inhibitory control)		•																																								•
OBT (Self-perspective processing)		•																																								•

105x49mm (300 x 300 DPI)

Daily_Diaries_Intervention

Start of Block: Intro

Q69 Please Enter Your ID Number

Remember, this is the last three letters of your phone number, then the last three characters of your postcode, then the last letter of your surname.

____

Q68 Thanks for your participation in our study so far. We have some questions about your day today that we'd like you to answer.

End of Block: Intro

Start of Block: Block 1

Q50 Thinking back over the day, please rate to what extent you have felt this mood (**happy**) today.

	1 - not at all (1)	2 (2)	3 (3)	4 (4)	5 (5)	6 (6)	7 - very much (7)
1 (1)	○	○	○	○	○	○	○

Q51 How much time in the day do you feel you have spent in this mood (**happy**).

	1 - very little (1)	2 (2)	3 (3)	4 (4)	5 (5)	6 (6)	7 - most of the day (7)
1 (1)	○	○	○	○	○	○	○

For peer review only - http://bmjopen.bmj.com/site/about/guidelines.xhtml

Page Break

For peer review only

For peer review only - http://bmjopen.bmj.com/site/about/guidelines.xhtml

Q52 Thinking back over the day, please rate to what extent you have felt this mood (**lively**) today.

	1 - not at all (1)	2 (2)	3 (3)	4 (4)	5 (5)	6 (6)	7 - very much (7)
1 (1)	○	○	○	○	○	○	○

Q53 How much time in the day do you feel you have spent in this mood (**lively**).

	1 - very little (1)	2 (2)	3 (3)	4 (4)	5 (5)	6 (6)	7 - most of the day (7)
1 (1)	○	○	○	○	○	○	○

Page Break

For peer review only

For peer review only - http://bmjopen.bmj.com/site/about/guidelines.xhtml

Q54 Thinking back over the day, please rate to what extent you have felt this mood (**content**) today.

	1 - not at all (1)	2 (2)	3 (3)	4 (4)	5 (5)	6 (6)	7 - very much (7)
1 (1)	○	○	○	○	○	○	○

Q55 How much time in the day do you feel you have spent in this mood (**content**).

	1 - very little (1)	2 (2)	3 (3)	4 (4)	5 (5)	6 (6)	7 - most of the day (7)
1 (1)	○	○	○	○	○	○	○

Page Break

For peer review only

For peer review only - http://bmjopen.bmj.com/site/about/guidelines.xhtml

Q56 Thinking back over the day, please rate to what extent you have felt this mood (**satisfied**) today.

	1 - not at all (1)	2 (2)	3 (3)	4 (4)	5 (5)	6 (6)	7 - very much (7)
1 (1)	○	○	○	○	○	○	○

Q57 How much time in the day do you feel you have spent in this mood (**satisfied**).

	1 - very little (1)	2 (2)	3 (3)	4 (4)	5 (5)	6 (6)	7 - most of the day (7)
1 (1)	○	○	○	○	○	○	○

Page Break

For peer review only

Q58 Thinking back over the day, please rate to what extent you have felt this mood (**depressed**) today.

	1 - not at all (1)	2 (2)	3 (3)	4 (4)	5 (5)	6 (6)	7 - very much (7)
1 (1)	○	○	○	○	○	○	○

Q59 How much time in the day do you feel you have spent in this mood (**depressed**).

	1 - very little (1)	2 (2)	3 (3)	4 (4)	5 (5)	6 (6)	7 - most of the day (7)
1 (1)	○	○	○	○	○	○	○

Page Break

For peer review only

For peer review only - http://bmjopen.bmj.com/site/about/guidelines.xhtml

Q60 Thinking back over the day, please rate to what extent you have felt this mood (**bored**) today.

	1 - not at all (1)	2 (2)	3 (3)	4 (4)	5 (5)	6 (6)	7 - very much (7)
1 (1)	○	○	○	○	○	○	○

Q61 How much time in the day do you feel you have spent in this mood (**bored**).

	1 - very little (1)	2 (2)	3 (3)	4 (4)	5 (5)	6 (6)	7 - most of the day (7)
1 (1)	○	○	○	○	○	○	○

Page Break

For peer review only

For peer review only - http://bmjopen.bmj.com/site/about/guidelines.xhtml

Q62 Thinking back over the day, please rate to what extent you have felt this mood (**anxious**) today.

	1 - not at all (1)	2 (2)	3 (3)	4 (4)	5 (5)	6 (6)	7 - very much (7)
1 (1)	○	○	○	○	○	○	○

Q63 How much time in the day do you feel you have spent in this mood (**anxious**).

	1 - very little (1)	2 (2)	3 (3)	4 (4)	5 (5)	6 (6)	7 - most of the day (7)
1 (1)	○	○	○	○	○	○	○

Page Break

For peer review only

Q64 Thinking back over the day, please rate to what extent you have felt this mood (**irritable**) today.

	1 - not at all (1)	2 (2)	3 (3)	4 (4)	5 (5)	6 (6)	7 - very much (7)
1 (1)	○	○	○	○	○	○	○

Q65 How much time in the day do you feel you have spent in this mood (**irritable**).

	1 - very little (1)	2 (2)	3 (3)	4 (4)	5 (5)	6 (6)	7 - most of the day (7)
1 (1)	○	○	○	○	○	○	○

Page Break

For peer review only

For peer review only - http://bmjopen.bmj.com/site/about/guidelines.xhtml

Q66 Thinking back over the day, please rate to what extent you have felt this mood (**tense**) today.

	1 - not at all (1)	2 (2)	3 (3)	4 (4)	5 (5)	6 (6)	7 - very much (7)
1 (1)	○	○	○	○	○	○	○

Q67 How much time in the day do you feel you have spent in this mood (**tense**).

	1 - very little (1)	2 (2)	3 (3)	4 (4)	5 (5)	6 (6)	7 - most of the day (7)
1 (1)	○	○	○	○	○	○	○

End of Block: Block 1

Start of Block: Block 2

Q41 Now, we just have some final questions...

Q42 Did you complete any of the exercises from this programme today?

○ Yes (1)

○ No (2)

Display This Question:

If Did you complete any of the exercises from this programme today? = Yes

For peer review only - http://bmjopen.bmj.com/site/about/guidelines.xhtml

Q43 How difficult did you find these exercises?

- 1 - Not at all difficult (2)
- 2 (3)
- 3 (4)
- 4 - Somewhat difficult (5)
- 5 (6)
- 6 (7)
- 7 - Extremely Difficult (8)

Display This Question:

If Did you complete any of the exercises from this programme today? = Yes

Q44
Did you find the skills you've been learning to be relevant at any stage during the day?

- 1 - Not At All Relevant (1)
- 2 (2)
- 3 (3)
- 4 - Somewhat (4)
- 5 (5)
- 6 (6)
- 7 - Extremely (7)

For peer review only

Display This Question:

If Did you complete any of the exercises from this programme today? = Yes

Q45 During the day, were you able to apply the skills you've learned during the exercises?

- 1 - Not At All (1)
- 2 (2)
- 3 (3)
- 4 - Somewhat (4)
- 5 (5)
- 6 (6)
- 7 - Absolutely (7)

Display This Question:

If Did you complete any of the exercises from this programme today? = Yes

Q49
During the day, how effortful was it to apply the skills you have learned ?

- 1- Not at all (1)
- 2 (2)
- 3 (3)
- 4 - Somewhat (4)
- 5 (5)
- 6 (6)
- 7 - Extremely effortful (7)

End of Block: Block 2

For peer review only - http://bmjopen.bmj.com/site/about/guidelines.xhtml

For peer review only

For peer review only - http://bmjopen.bmj.com/site/about/guidelines.xhtml

Appendix A

Tim Dalgleish
Principal Investigator
MRC Cognition and Brain Sciences Unit
University of Cambridge
15 Chaucer Road,
Cambridge, CB2 7EF
Phone: 01223 766166
myriad@mrc-cbu.cam.ac.uk

Participant Consent Form

Study title: Investigating the neuro-cognitive dynamics of decentring training and its impact on emotional reactivity: A Longitudinal and multimodal assessment.

We are interested in how new therapeutic techniques like mindfulness and decentring affect your thoughts and feelings.

This study has been reviewed by the Cambridge Psychology Research Ethics Committee [PRE.2019.036]

Please initial at the end of each line and then sign below to indicate that you agree to take part in the study:

• Read and understood the information sheet for this study

……………

• Had the opportunity to consider the information and ask questions via the email address or phone number given at the top of this sheet

……………

• Had your questions answered satisfactorily

……………

• Understood that this project will require you to have an app installed on your smartphone for the duration of the project

……………

• Understood that your participation is voluntary and that he or she can withdraw at any time without giving a reason

……………

For peer review only

• Understood that your data and personal details will be stored on secure servers. At the end of the study, the data will be transferred and held at The MRC Cognition and Brain Sciences Unit in a secure database and will be accessed by the academic community

………………

• The MRC Cognition and Brain Sciences Unit will take all reasonable steps to protect your privacy. Your data will be coded and will not be associated with your name or information that could reveal your identity. This anonymised data will be made freely available to the wider academic community ……………

• Understood that all information will be treated as strictly confidential and handled in accordance with the provisions of the Data Protection Act 2018

……………

• Understood that in all instances where the results are disseminated they will remain anonymous

……………

PLEASE USE BLOCK CAPITAL LETTERS WHEN FILLING IN THIS FORM

Your name…… …………

Your school/college…..……… …..................

Gender ………………………. Year group………………………. Date of birth……………………………………….

Other information. Does you have any condition that may affect your performance on the tasks? If so please state: ………………………………………………………………..

Would you like to be contacted in the future about participating in other studies (please circle)?
YES NO

Your email………

Please sign below to indicate that you agree to take part in the study:

Your signature: ……... Date: ....

For peer review only

Figure S1 – Affective Cognitive Control Task

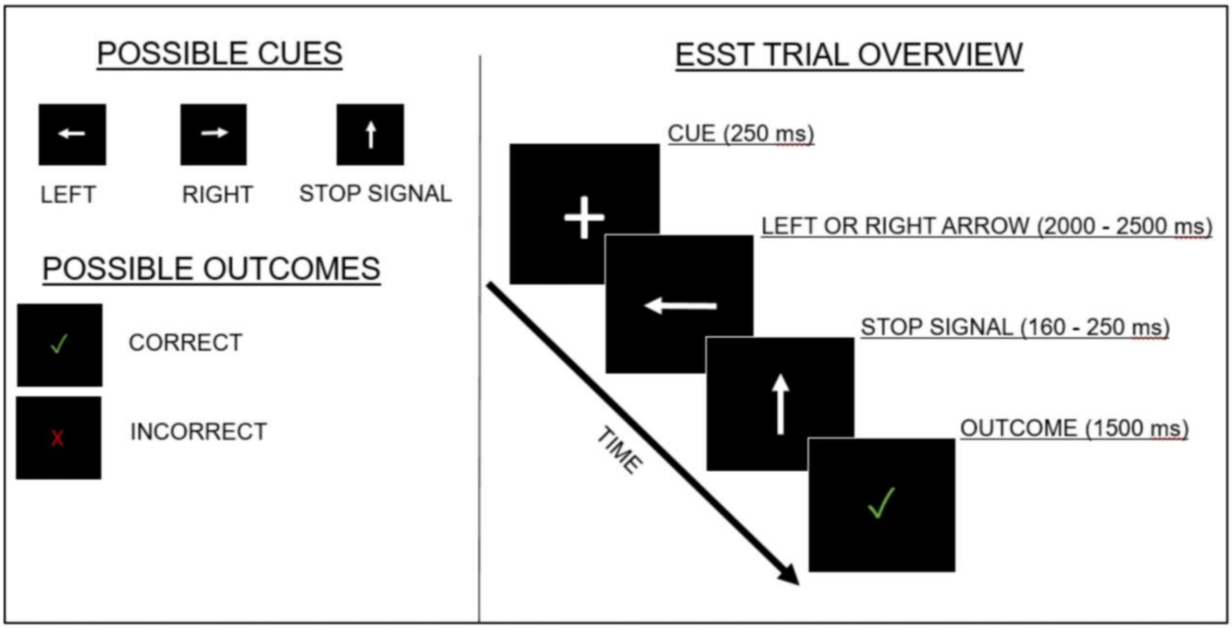

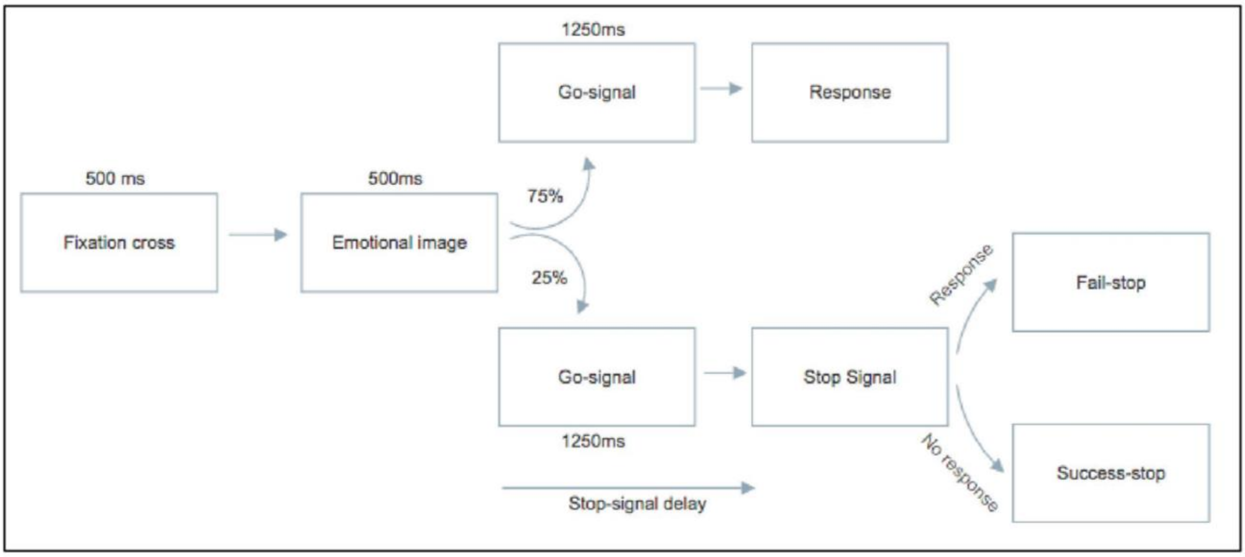

Across multiple trials, participants respond to a go-signal (e.g. press-left to a left arrow) unless it is followed by a stop-signal (an upwards arrow); these stop-signals follow 20% of go-signals. In the emotional stop-signal task (eSST) go-signals are preceded by a neutral or negative image from the IAPS (IAPS; Lang et al., 1992). We will measure accuracy and neurophysiological activities during response inhibition.

Figure S2 – Self Referential Processing Task

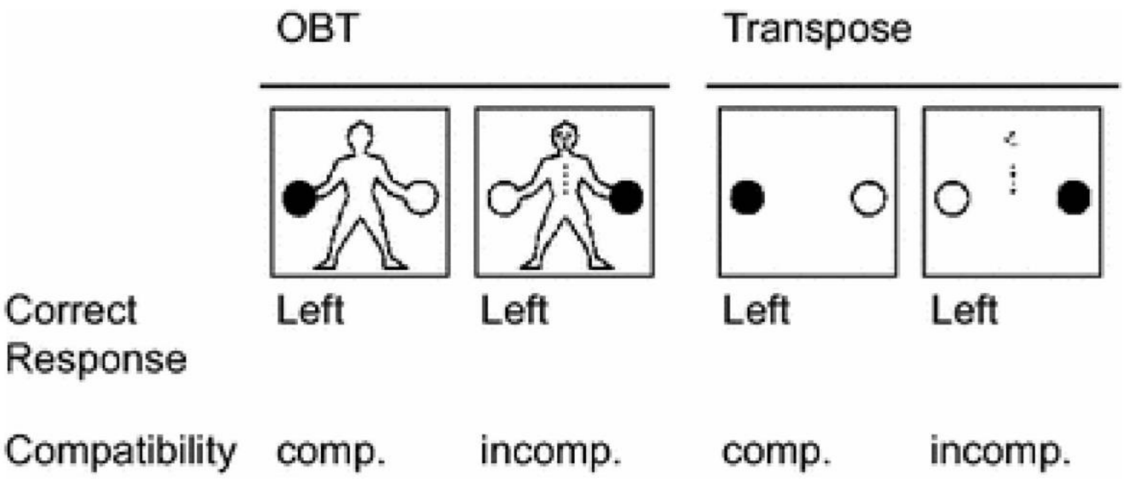

For example, the Own Body Task requires speeded spatial judgments made from the perspective of a schematic figure. The stimulus is a figure holding a black ball in one hand and a white ball in the other. The black ball was either in the figure's left or right hand and the figure could be facing either toward or away from the participant, but always in an upright orientation. Participants were instructed to imagine themselves in the body position of the figure, in order to judge whether the black ball was being held by the figure's left or right hand by pressing the corresponding response keys. We will measure how quickly participants are able to judge which hand the black ball is in, and whether they can correctly judge this.

Reporting checklist for protocol of a clinical trial.

Based on the SPIRIT guidelines.

Instructions to authors

Complete this checklist by entering the page numbers from your manuscript where readers will find each of the items listed below.

Your article may not currently address all the items on the checklist. Please modify your text to include the missing information. If you are certain that an item does not apply, please write "n/a" and provide a short explanation.

Upload your completed checklist as an extra file when you submit to a journal.

In your methods section, say that you used the SPIRITreporting guidelines, and cite them as:

Chan A-W, Tetzlaff JM, Gøtzsche PC, Altman DG, Mann H, Berlin J, Dickersin K, Hróbjartsson A, Schulz KF, Parulekar WR, Krleža-Jerić K, Laupacis A, Moher D. SPIRIT 2013 Explanation and Elaboration: Guidance for protocols of clinical trials. BMJ. 2013;346:e7586

		Reporting Item	Page Number
**Administrative information**			
Title	#1	Descriptive title identifying the study design, population, interventions, and, if applicable, trial acronym	1
Trial registration	#2a	Trial identifier and registry name. If not yet registered, name of intended registry	1
Trial registration: data set	#2b	All items from the World Health Organization Trial Registration Data Set	1
Protocol version	#3	Date and version identifier	1
Funding	#4	Sources and types of financial, material, and other support	1
Roles and responsibilities: contributorship	#5a	Names, affiliations, and roles of protocol contributors	32

For peer review only

Roles and responsibilities: sponsor contact information	#5b	Name and contact information for the trial sponsor	1
Roles and responsibilities: sponsor and funder	#5c	Role of study sponsor and funders, if any, in study design; collection, management, analysis, and interpretation of data; writing of the report; and the decision to submit the report for publication, including whether they will have ultimate authority over any of these activities	N/A - Study Sponsor and Funders had no role
Roles and responsibilities: committees	#5d	Composition, roles, and responsibilities of the coordinating centre, steering committee, endpoint adjudication committee, data management team, and other individuals or groups overseeing the trial, if applicable (see Item 21a for data monitoring committee)	N/A - No Data Monitoring Committee was used
**Introduction**			
Background and rationale	#6a	Description of research question and justification for undertaking the trial, including summary of relevant studies (published and unpublished) examining benefits and harms for each intervention	1
Background and rationale: choice of comparators	#6b	Explanation for choice of comparators	12
Objectives	#7	Specific objectives or hypotheses	6
Trial design	#8	Description of trial design including type of trial (eg, parallel group, crossover, factorial, single group), allocation ratio, and framework (eg, superiority, equivalence, non-inferiority, exploratory)	7
**Methods: Participants, interventions, and outcomes**			

For peer review only

Study setting	#9	Description of study settings (eg, community clinic, academic hospital) and list of countries where data will be collected. Reference to where list of study sites can be obtained	8
Eligibility criteria	#10	Inclusion and exclusion criteria for participants. If applicable, eligibility criteria for study centres and individuals who will perform the interventions (eg, surgeons, psychotherapists)	8
Interventions: description	#11a	Interventions for each group with sufficient detail to allow replication, including how and when they will be administered	10
Interventions: modifications	#11b	Criteria for discontinuing or modifying allocated interventions for a given trial participant (eg, drug dose change in response to harms, participant request, or improving / worsening disease)	11
Interventions: adherance	#11c	Strategies to improve adherence to intervention protocols, and any procedures for monitoring adherence (eg, drug tablet return; laboratory tests)	11
Interventions: concomitant care	#11d	Relevant concomitant care and interventions that are permitted or prohibited during the trial	8
Outcomes	#12	Primary, secondary, and other outcomes, including the specific measurement variable (eg, systolic blood pressure), analysis metric (eg, change from baseline, final value, time to event), method of aggregation (eg, median, proportion), and time point for each outcome. Explanation of the clinical relevance of chosen efficacy and harm outcomes is strongly recommended	13
Participant timeline	#13	Time schedule of enrolment, interventions (including any run-ins and washouts), assessments, and visits for participants. A schematic diagram is highly recommended (see Figure)	18
Sample size	#14	Estimated number of participants needed to achieve study objectives and how it was	9

For peer review only

		determined, including clinical and statistical assumptions supporting any sample size calculations	
Recruitment	#15	Strategies for achieving adequate participant enrolment to reach target sample size	9
**Methods: Assignment of interventions (for controlled trials)**			
Allocation: sequence generation	#16a	Method of generating the allocation sequence (eg, computer-generated random numbers), and list of any factors for stratification. To reduce predictability of a random sequence, details of any planned restriction (eg, blocking) should be provided in a separate document that is unavailable to those who enrol participants or assign interventions	18
Allocation concealment mechanism	#16b	Mechanism of implementing the allocation sequence (eg, central telephone; sequentially numbered, opaque, sealed envelopes), describing any steps to conceal the sequence until interventions are assigned	18
Allocation: implementation	#16c	Who will generate the allocation sequence, who will enrol participants, and who will assign participants to interventions	18
Blinding (masking)	#17a	Who will be blinded after assignment to interventions (eg, trial participants, care providers, outcome assessors, data analysts), and how	19
Blinding (masking): emergency unblinding	#17b	If blinded, circumstances under which unblinding is permissible, and procedure for revealing a participant's allocated intervention during the trial	19
**Methods: Data collection, management, and analysis**			

For peer review only

Data collection plan	#18a	Plans for assessment and collection of outcome, baseline, and other trial data, including any related processes to promote data quality (eg, duplicate measurements, training of assessors) and a description of study instruments (eg, questionnaires, laboratory tests) along with their reliability and validity, if known. Reference to where data collection forms can be found, if not in the protocol	13
Data collection plan: retention	#18b	Plans to promote participant retention and complete follow-up, including list of any outcome data to be collected for participants who discontinue or deviate from intervention protocols	20
Data management	#19	Plans for data entry, coding, security, and storage, including any related processes to promote data quality (eg, double data entry; range checks for data values). Reference to where details of data management procedures can be found, if not in the protocol	21
Statistics: outcomes	#20a	Statistical methods for analysing primary and secondary outcomes. Reference to where other details of the statistical analysis plan can be found, if not in the protocol	21
Statistics: additional analyses	#20b	Methods for any additional analyses (eg, subgroup and adjusted analyses)	21
Statistics: analysis population and missing data	#20c	Definition of analysis population relating to protocol non-adherence (eg, as randomised analysis), and any statistical methods to handle missing data (eg, multiple imputation)	21
**Methods: Monitoring**			
Data monitoring: formal committee	#21a	Composition of data monitoring committee (DMC); summary of its role and reporting structure; statement of whether it is independent from the sponsor and competing interests; and reference to	21

For peer review only

		where further details about its charter can be found, if not in the protocol. Alternatively, an explanation of why a DMC is not needed	
Data monitoring: interim analysis	#21b	Description of any interim analyses and stopping guidelines, including who will have access to these interim results and make the final decision to terminate the trial	21
Harms	#22	Plans for collecting, assessing, reporting, and managing solicited and spontaneously reported adverse events and other unintended effects of trial interventions or trial conduct	22
Auditing	#23	Frequency and procedures for auditing trial conduct, if any, and whether the process will be independent from investigators and the sponsor	N/A - Small scale trial so data will not be audited
**Ethics and dissemination**			
Research ethics approval	#24	Plans for seeking research ethics committee / institutional review board (REC / IRB) approval	22
Protocol amendments	#25	Plans for communicating important protocol modifications (eg, changes to eligibility criteria, outcomes, analyses) to relevant parties (eg, investigators, REC / IRBs, trial participants, trial registries, journals, regulators)	22
Consent or assent	#26a	Who will obtain informed consent or assent from potential trial participants or authorised surrogates, and how (see Item 32)	19
Consent or assent: ancillary studies	#26b	Additional consent provisions for collection and use of participant data and biological specimens in ancillary studies, if applicable	33
Confidentiality	#27	How personal information about potential and enrolled participants will be collected, shared, and maintained in order to protect confidentiality before, during, and after the trial	20

For peer review only

Declaration of interests	#28	Financial and other competing interests for principal investigators for the overall trial and each study site	1
Data access	#29	Statement of who will have access to the final trial dataset, and disclosure of contractual agreements that limit such access for investigators	1
Ancillary and post trial care	#30	Provisions, if any, for ancillary and post-trial care, and for compensation to those who suffer harm from trial participation	20
Dissemination policy: trial results	#31a	Plans for investigators and sponsor to communicate trial results to participants, healthcare professionals, the public, and other relevant groups (eg, via publication, reporting in results databases, or other data sharing arrangements), including any publication restrictions	23
Dissemination policy: authorship	#31b	Authorship eligibility guidelines and any intended use of professional writers	23
Dissemination policy: reproducible research	#31c	Plans, if any, for granting public access to the full protocol, participant-level dataset, and statistical code	23
**Appendices**			
Informed consent materials	#32	Model consent form and other related documentation given to participants and authorised surrogates	33
Biological specimens	#33	Plans for collection, laboratory evaluation, and storage of biological specimens for genetic or molecular analysis in the current trial and for future use in ancillary studies, if applicable	N/A - None collected

Notes:

- 5c: N/A - Study Sponsor and Funders had no role
- 5d: N/A - No Data Monitoring Committee was used
- 23: N/A - Small scale trial so data will not be audited

For peer review only

- 33: N/A - None collected The SPIRIT Explanation and Elaboration paper is distributed under the terms of the Creative Commons Attribution License CC-BY-NC. This checklist was completed on 08. July 2021 using https://www.goodreports.org/, a tool made by the EQUATOR Network in collaboration with Penelope.ai

For peer review only
